# Twelve 4-(4-meth­oxy­phen­yl)piperazin-1-ium salts containing organic anions: supra­molecular assembly in one, two and three dimensions

**DOI:** 10.1107/S2056989019012702

**Published:** 2019-09-20

**Authors:** Haruvegowda Kiran Kumar, Hemmige S. Yathirajan, Sabine Foro, Christopher Glidewell

**Affiliations:** a Department of Studies in Chemistry, University of Mysore, Manasagangotri, Mysuru-570 006, India; b Institute of Materials Science, Darmstadt University of Technology, Petersenstrasse 23, D-64287 Darmstadt, Germany; c School of Chemistry, University of St Andrews, St Andrews, Fife KY16 9ST, UK

**Keywords:** synthesis, piperazines, crystal structure, disorder, twinning, hydrogen bonding, supra­molecular assembly

## Abstract

Twelve 4-(4-meth­oxy­phen­yl)piperazin-1-ium salts containing organic anions have been prepared and structurally characterized. The monohydrated benzoate, 4-fluoro­benzoate, 4-chloro­benzoate and 4-bromo­benzoate salts, C_11_H_17_N_2_O^+^·C_7_H_5_O_2_^−^·H_2_O (I), C_11_H_17_N_2_O^+^·C_7_H_4_FO_2_^−^·H_2_O (II), C_11_H_17_N_2_O^+^·C_7_H_4_ClO_2_^−^·H_2_O (III), and C_11_H_17_N_2_O^+^·C_7_H_4_BrO_2_^−^·H_2_O (IV), respectively, are isomorphous and all exhibit disorder in the 4-meth­oxy­phenyl unit: the components are linked by N—H⋯O and O—H⋯O hydrogen bond to form chains of rings. The unsolvated 2-hy­droxy­benzoate, pyridine-3-carboxyl­ate and 2-hy­droxy-3,5-di­nitro­benzoate salts, C_11_H_17_N_2_O^+^·C_7_H_5_O_3_^−^ (V), C_11_H_17_N_2_O^+^·C_6_H_4_NO_2_^−^ (VI) and C_11_H_17_N_2_O^+^·C_7_H_3_N_2_O_7_^−^ (VII), respectively, are all fully ordered: the components of (V) are linked by multiple N—H⋯O hydrogen bonds to form a chain of rings; those of (VI) are linked into a three-dimensional framework by a combination of N—H⋯O, C—H⋯O and C—H⋯N hydrogen bonds and those of (VII), where the anion has a structure reminiscent of the picrate anion, are linked into a three-dimensional array by N—H⋯O and C—H⋯O hydrogen bonds. The hydrogensuccinate and hydrogenfumarate salts, C_11_H_17_N_2_O^+^·C_4_H_5_O_4_^−^ (VIII) and C_11_H_17_N_2_O^+^·C_4_H_3_O_3_^−^ (IX), respectively, are isomorphous, and both exhibit disorder in the anionic component: N—H⋯O and O—H⋯O hydrogen bonds link the ions into sheets, which are further linked by C—H⋯π(arene) inter­actions. The anion of the hydrogenmaleate salt, C_11_H_17_N_2_O^+^·C_4_H_3_O_3_^−^ (X), contains a very short and nearly symmetrical O⋯H⋯O hydrogen bond, and N—H⋯O hydrogen bonds link the anions into chains of rings. The ions in the tri­chloro­acetate salt, C_11_H_17_N_2_O^+^·C_2_Cl_3_O_2_^−^ (XI), are linked into simple chains by N—H⋯O hydrogen bonds. In the hydrated chloranilate salt, 2C_11_H_17_N_2_O^+^·C_6_Cl_2_O_4_^2−^·2H_2_O (XII), which crystallizes as a non-merohedral twin, the anion lies across a centre of inversion in space group *P*2_1_/*n*, and a combination of N—H⋯O and O—H⋯O hydrogen bonds generates complex sheets. Comparisons are made with the structures of some related compounds.

## Abstract 2

In twelve 4-(4-meth­oxy­phen­yl)piperazin-1-ium salts containing organic anions, the hydrogen-bonded supra­molecular assembly ranges from simple chains *via* chains of rings and sheets to three-dimensional framework structures.

## Chemical context   

**Figure 1:**
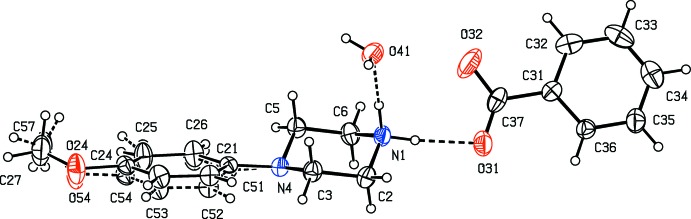
The independent components of compound (I), showing the atom-labelling scheme, the disorder of the 4-meth­oxy­phenyl group, and the hydrogen bonds within the selected asymmetric unit. The major disorder component is drawn using full lines and the minor disorder component is drawn using dashed lines. Displacement ellipsoids are drawn at the 30% probability level and, for the sake of clarity, a few of the atom labels have been omitted.

In recent years, *N*-(4-meth­oxy­phen­yl)piperazine (MeOPP) has emerged as a new addition to the range of designer recreational drugs, and considerable effort has been invested in the development of methods for the detection both of MeOPP itself and of its metabolites *N*-(4-hy­droxy­phen­yl)piperazine and 4-hy­droxy­aniline (Arbo *et al.*, 2012 ▸) in human fluids (Staack & Maurer, 2003 ▸; Staack *et al.*, 2004 ▸). MeOPP has euphoric stimulant properties and its action on human physiology is similar to that of amphetamines (Staack & Maurer, 2005 ▸; Wohlfarth *et al.*, 2010 ▸), but it has a significantly lower potential for abuse (Nagai *et al.*, 2007 ▸). However, no therapeutic applications of MeOPP have been reported to date. In view of the reported properties of MeOPP, coupled with the broad range of biological activities exhibited by piperazine derivatives (Asif, 2015 ▸; Brito *et al.*, 2019 ▸), we have recently initiated a programme of study centred on *N*-(4-meth­oxy­phen­yl)piperazine derivatives, and we have recently reported the synthesis and structures of a series of 1-aroyl-4-(4-meth­oxy­phen­yl)piperazines (Kiran Kumar *et al.*, 2019 ▸). In a continuation of that work, we have now prepared a series of 4-meth­oxy­phen­yl)piperazin-1-ium salts of simple organic acids, (I)–(XII), in order to study the various patterns of hydrogen-bonding inter­actions present in these salts, which may eventually be of value in pharmacological and pharmaceutical applications (Kavitha *et al.*, 2014 ▸; Kaur *et al.*, 2015 ▸; Shaibah, Yathirajan *et al.*, 2017 ▸; Shaibah, Sagar *et al.*, 2017 ▸; Shaibah *et al.*, 2019 ▸). Salts of this type are readily prepared by co-crystallizations of the piperazine and the acids in methanol solution and, in total, 28 different acids representing a wide range of chemical types were investigated (see Section 5): however, only twelve of these provided crystals suitable for single-crystal X-ray diffraction, and thus we report here the mol­ecular and supra­molecular structures of (I)–(XII) (Figs. 1 ▸–12 ▸ ▸ ▸ ▸ ▸ ▸ ▸ ▸ ▸ ▸ ▸).
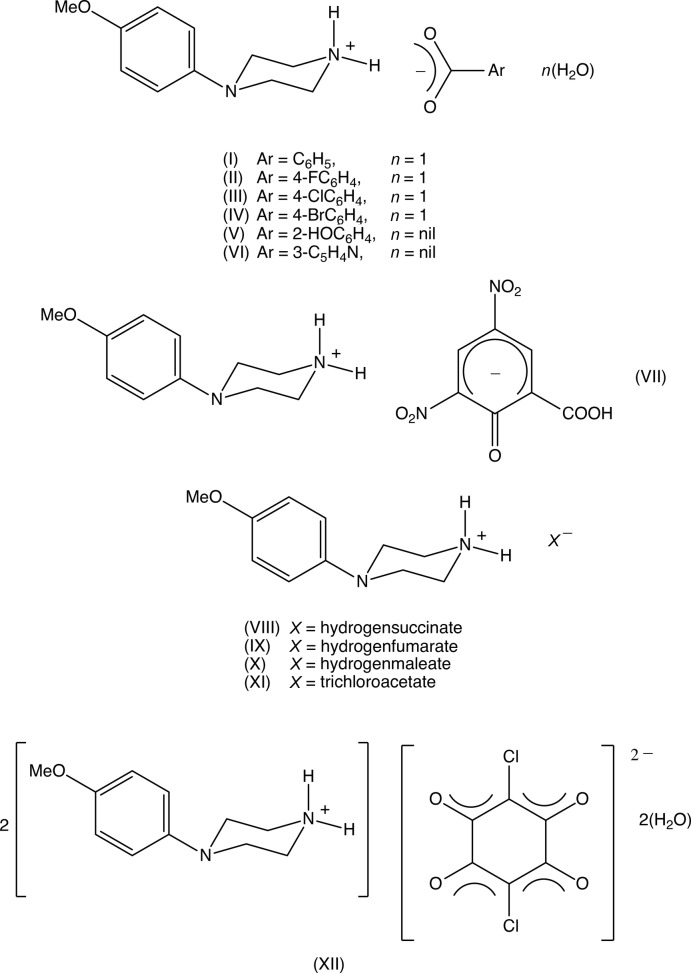


## Structural commentary   

Compounds (I)–(XI) are all 1:1 salts, but in (XII), where the dianion lies across a centre of inversion while the cation lies in a general position, the cation:anion ratio is 2:1. Compounds (I)–(IV) and (XII) all crystallize as hydrates, but compounds (V)–(XI) all crystallize in solvent-free form. Compounds (I)–(IV) are isomorphous (Table 2 ▸), in each of which the 4-meth­oxy­phenyl groups is disordered over two sets of atomic sites (Figs. 1 ▸–4 ▸ ▸ ▸), having occupancies 0.66 (2) and 0.34 (2) in (I), 0.81 (3) and 0.19 (3) in (II), 0.73 (2) and 0.27 (2) in (III) and 0.80 (2) and 0.20 (2) in (IV). Similarly, compounds (VIII) and (IX) are isomorphous, and in both of them the anion exhibits disorder, with occupancies of 0.660 (15) and 0.340 (15) in (VIII), and 0.906 (9) and 0.094 (9) in (IX) (Figs. 8 ▸ and 9 ▸). While compounds (I)–(IV) are isostructural, compounds (VIII) and (IX) are not, because of both the different configurations of their anions and the different degrees of disorder. Examples have been previously reported of compounds that are isomorphous but not strictly isostructural in terms of their inter­molecular inter­actions (Acosta *et al.*, 2009 ▸).

In the anion of compound (VII), the carboxyl group is unionized, with C—O distances of 1.220 (3) and 1.309 (3) Å and it is the phenolic H atom which has been lost (Fig. 7 ▸). The C32—O33 distance, 1.280 (3) Å, is closer to that normally found in ketones than to that typical of phenols or phenolates (Allen *et al.*, 1987 ▸): in addition, the C31—C32 and C32—C33 distances, 1.437 (4) and 1.430 (4) Å, respectively, are significantly larger than the other C—C distances in this ring, which lie in the rather narrow range 1.370 (3)–1.385 (4) Å, but the C—N and N—O distances are all typical of their types (Allen *et al.*, 1987 ▸). These observations indicate that the negative charge in this anion is delocalized over the five atoms C31, C33, C34, C35 and C36, but without any significant delocalization onto the nitro groups, as has been observed in tri­nitro­phenolate (picrate) anions (Kavitha *et al.*, 2006 ▸; Sagar *et al.*, 2017 ▸; Shaibah *et al.*, 2017*a* ▸,*b* ▸).

The anion of compound (X) contains an almost linear and very short (Emsley, 1980 ▸; Herschlag & Pinney, 2018 ▸) O⋯H⋯O hydrogen bond, in which the H atom is almost, but not exactly, centred between the two O atoms (Table 1 ▸). In the centrosymmetric anion of compound (XII) (Fig. 12 ▸), the two independent C—O distances are identical within experimental uncertainty, 1.244 (2) and 1.246 (2) Å, as are the distances C31—C32 and C32—C33, 1.398 (3) and 1.392 (2) Å. However, the remaining C—C distance in this ring, 1.539 (3) Å is typical of a single C—C bond (Allen *et al.*, 1987 ▸). These observations indicates the delocalization of a negative charge across each of the O–C–C–C–O units, and that these two units are effectively isolated from each other electronically. Despite the apparent simplicity of this dianion, with its high intrinsic symmetry, it is not possible adequately to describe its electronic structure in a single diagrammatic form, and four forms (*A*)–(*D*) (Fig. 13 ▸) are required.

## Supra­molecular features   

In each of the four isomorphous salts (I)–(IV), the ions are linked by a combination of N—H⋯O and O—H⋯O hydrogen bonds (Table 1 ▸) to form a chain of edge-fused centrosymmetric rings running parallel to the [100] direction, in which 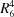
(12) (Etter, 1990 ▸; Etter *et al.*, 1990 ▸; Bernstein *et al.*, 1995 ▸) rings centred at (*n*, 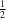, 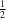) alternate with 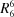
(16) rings centred at (*n* + 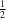, 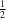, 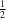) , where *n* represents an integer in each case (Fig. 14 ▸). In each of these four salts, a combination of C—H⋯O and C—H⋯π(arene) hydrogen bonds links the [100] chain into complex sheets lying parallel to (001).

**Figure 5:**
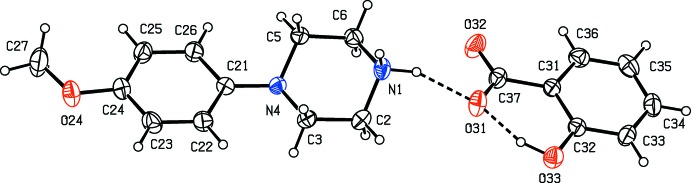
The independent components of compound (V), showing the atom-labelling scheme and the hydrogen bonds within the selected asymmetric unit. Displacement ellipsoids are drawn at the 30% probability level.

There is an inter­molecular O—H⋯O hydrogen bond in the anion of the unsolvated salt (V). The two anions in the selected asymmetric unit (Fig. 5 ▸) are linked by an asymmetric three-centre N—H⋯(O)_2_ hydrogen bond, and the resulting ion pairs, which are related by 2_1_ screw axis along (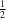, *y*, 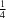), are linked by a two-centre N—H⋯O hydrogen bond to form chain of rings running parallel to the [010] direction (Fig. 15 ▸). Chains of this type are weakly linked into sheets lying parallel to (001) by a combination of C—H⋯O and C—H⋯π(arene) hydrogen bonds.

**Figure 6:**
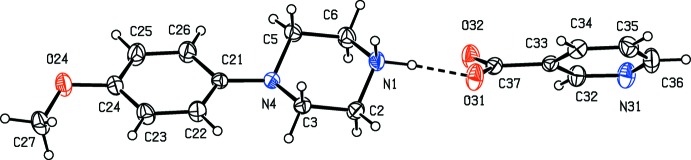
The independent components of compound (VI), showing the atom-labelling scheme and the hydrogen bonds within the selected asymmetric unit. Displacement ellipsoids are drawn at the 30% probability level.

The component ions in compound (VI) (Fig. 6 ▸) are linked by a two-centre N—H⋯O hydrogen bond and the resulting ion pairs are further linked by a combination of N—H⋯O, C—H⋯O and C—H⋯N hydrogen bonds to form a three-dimensional framework structure, whose formation can readily be analysed in terms of three simple sub-structures (Ferguson *et al.*, 1998*a* ▸,*b* ▸; Gregson *et al.*, 2000 ▸). Ion pairs which are related by the *b*-glide plane at *x* = 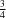 are linked by a second N—H⋯O hydrogen bond to form a 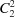
(6) chain running parallel to the [010] direction, and in the second sub-structure, ion pairs which are related by the *c*-glide plane at *y* = 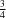 are linked by a C—H⋯O hydrogen bond (Table 1 ▸) to form a 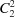
(17) chain running parallel to the [001] direction. The combination of these two simple chain motifs generates a sheet of 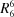
(40) rings lying parallel to (100) in the domain 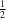 < *x* < 1.0 (Fig. 16 ▸). A second sheet of this type, related to the first by inversion lies in the domain 0 < *x* < 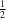, and adjacent sheets are linked by the third sub-structure in which inversion-related ion pairs are linked by C—H⋯N hydrogen bonds into a centrosymmetric 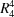
(18) ring (Fig. 17 ▸): the action of this inter­action is to link all of the (100) sheets into a continuous three-dimensional array.

**Figure 7:**
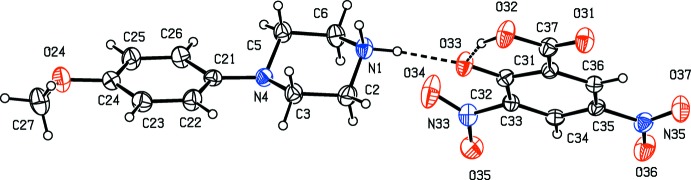
The independent components of compound (VII), showing the atom-labelling scheme and the hydrogen bond within the selected asymmetric unit. Displacement ellipsoids are drawn at the 30% probability level.

There is an inter­molecular O—H⋯O hydrogen bond in the anion of compound (VII) (Fig. 7 ▸), but the carboxyl H atom plays no part in the supra­molecular assembly. The ions are linked by a combination of N—H⋯O and C—H⋯O hydrogen bonds to form a chain of centrosymmetric rings running parallel to the [210] direction, in which 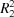
(10) rings centred at (2*n* − 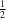, *n*, 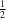) alternate with 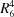
(16) rings centred at (2*n* + 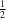, *n* + 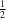, 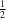), where *n* represents an integer in each case (Fig. 18 ▸). Two chains of this type, related to one another by the translational symmetry operations, pass through each unit cell, and a weak C—H⋯π(arene) hydrogen bond links the chains into a three-dimensional framework structure.

**Figure 8:**
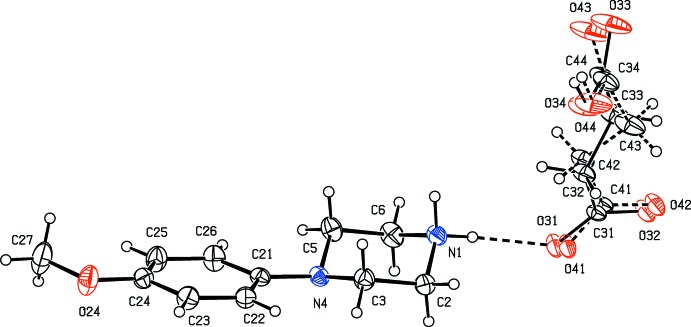
The independent components of compound (VIII), showing the atom-labelling scheme, the disorder of anion, and the hydrogen bonds within the selected asymmetric unit. The major disorder component is drawn using full lines and the minor disorder component is drawn using dashed lines. Displacement ellipsoids are drawn at the 30% probability level.

**Figure 12:**
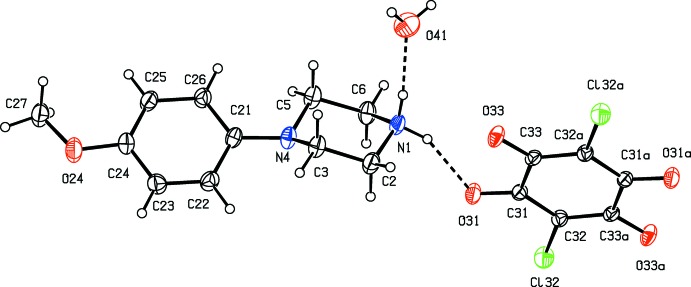
The independent components of compound (XII), showing the atom-labelling scheme and the hydrogen bonds within the selected asymmetric unit. Displacement ellipsoids are drawn at the 30% probability level, and the atoms marked with the suffix ‘a′ are at the symmetry position (1 − *x*, 1 − *y*, −*z*)

**Figure 13:**
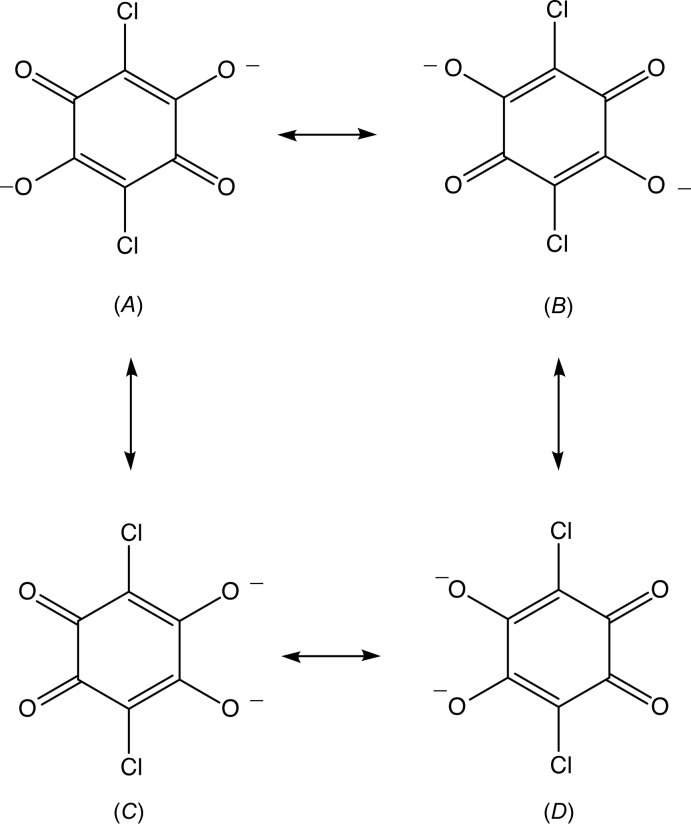
The canonical forms of the anion in compound (XII).

For the disordered structure of compound (VIII), the hydrogen bonds formed by the major and minor disorder components are very similar (Table 1 ▸) so that only the major disorder form need be considered in detail. Within the selected asymmetric unit (Fig. 8 ▸), the component ions are linked by a two-centre N—H⋯O hydrogen bond: the ion pairs are linked by a combination of N—H⋯O and O—H⋯O hydrogen bonds to form sheets, whose formation can readily be analysed in terms of two simple sub-structures. In the simpler of these, anions which are related by the *a*-glide plane at *y* = 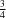 are linked by O—H⋯O hydrogen bonds into *C*(7) chains running parallel to the [10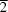] direction (Fig. 19 ▸); in the second sub-structure, ion pairs which are related by the same glide plane are linked by N—H⋯O hydrogen bonds to form a 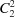
(6) chain running parallel to the [100] direction (Fig. 20 ▸). The combination of these two chain motifs generates a sheet lying parallel to (010), and a single C—H⋯π(arene) hydrogen bond links these sheets into a three-dimensional framework structure. The supra­molecular aggregation in the isomorphous compound (IX) is similar to that in (VIII). As noted in Section 2 above, the anion in compound (X) contains a very short and nearly symmetrical O⋯H⋯O hydrogen bond. Within the selected asymmetric unit, the component ions are linked by the three-centre N—H⋯(O)_2_ hydrogen bond and ion pairs which are related by translation are linked by a two-centre N—H⋯O hydrogen bond to form a *C*(9)*C*(9)[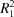
(4)] chain of rings running parallel to the [100] direction (Fig. 21 ▸). The C—H⋯O contact is at the margin of significance (Wood *et al.*, 2009 ▸), but it involves chains related by inversion.

The supra­molecular assembly of compound (XI) is extremely simple: two N—H⋯O hydrogen bonds link the ions into a 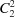
(6) chain running parallel to the [100] direction (Fig. 22 ▸). In compound (XII), a combination of N—H⋯O and O—H⋯O hydrogen bonds links all three components into a chain of 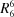
(18) rings running parallel to the [001] direction (Fig. 23 ▸), while a second O—H⋯O hydrogen bond links a combination of cations and water mol­ecules into a simple 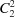
(12) chain running parallel to the [101] direction (Fig. 24 ▸) and the combination of these two chain motifs generates a complex sheet lying parallel to (010).

**Figure 14:**
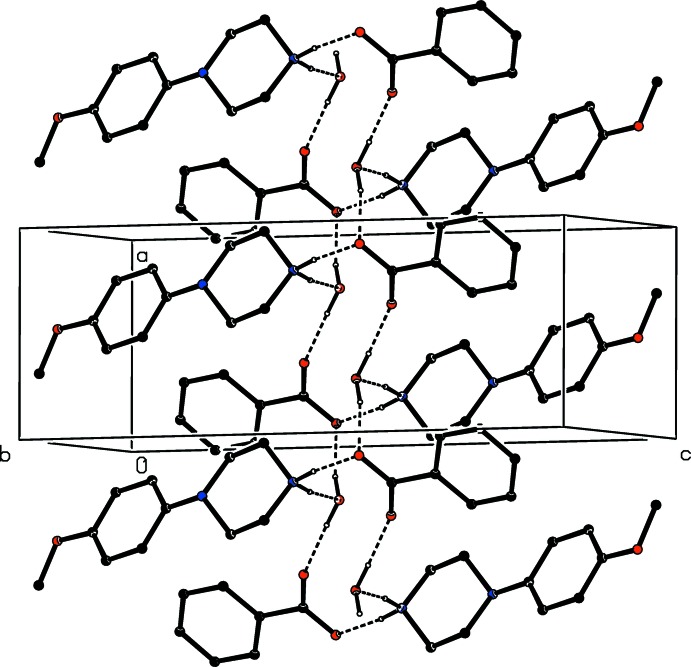
Part of the crystal structure of compound (I) showing the formation of a chain of rings parallel to the [100] direction. Hydrogen bonds are drawn as dashed lines and, for the sake of clarity, the minor disorder component and the H atoms bonded to C atoms have been omitted.

**Figure 15:**
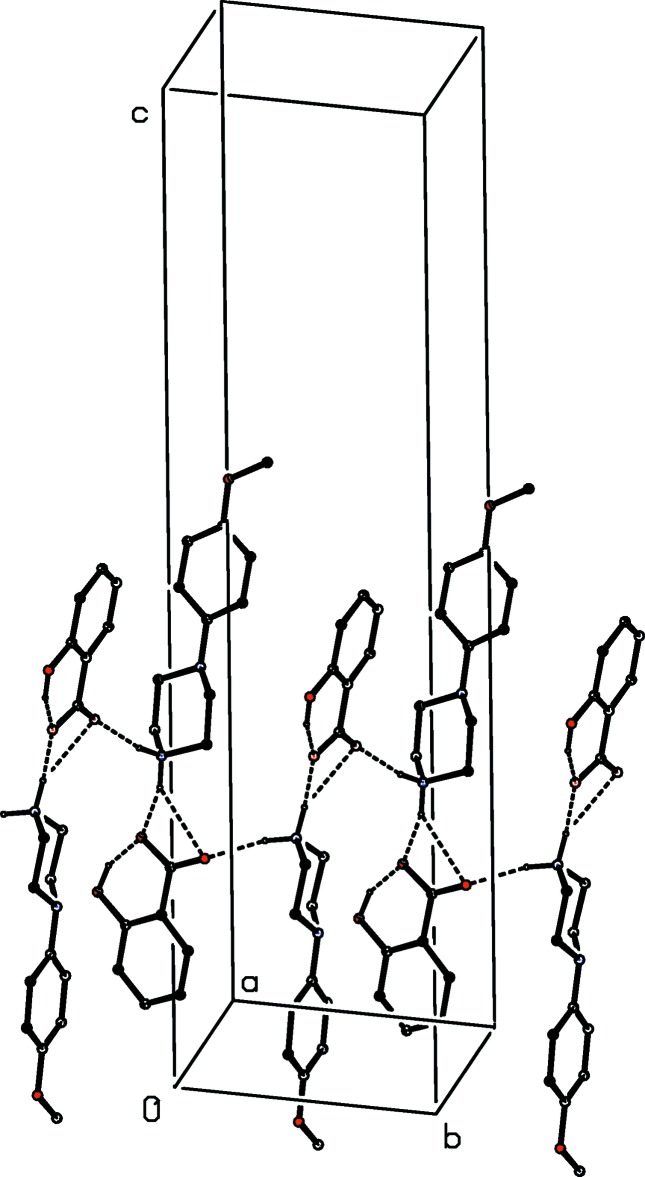
Part of the crystal structure of compound (V) showing the formation of a chain of rings parallel to the [010] direction. Hydrogen bonds are drawn as dashed lines and, for the sake of clarity, the H atoms bonded to C atoms have been omitted.

**Figure 16:**
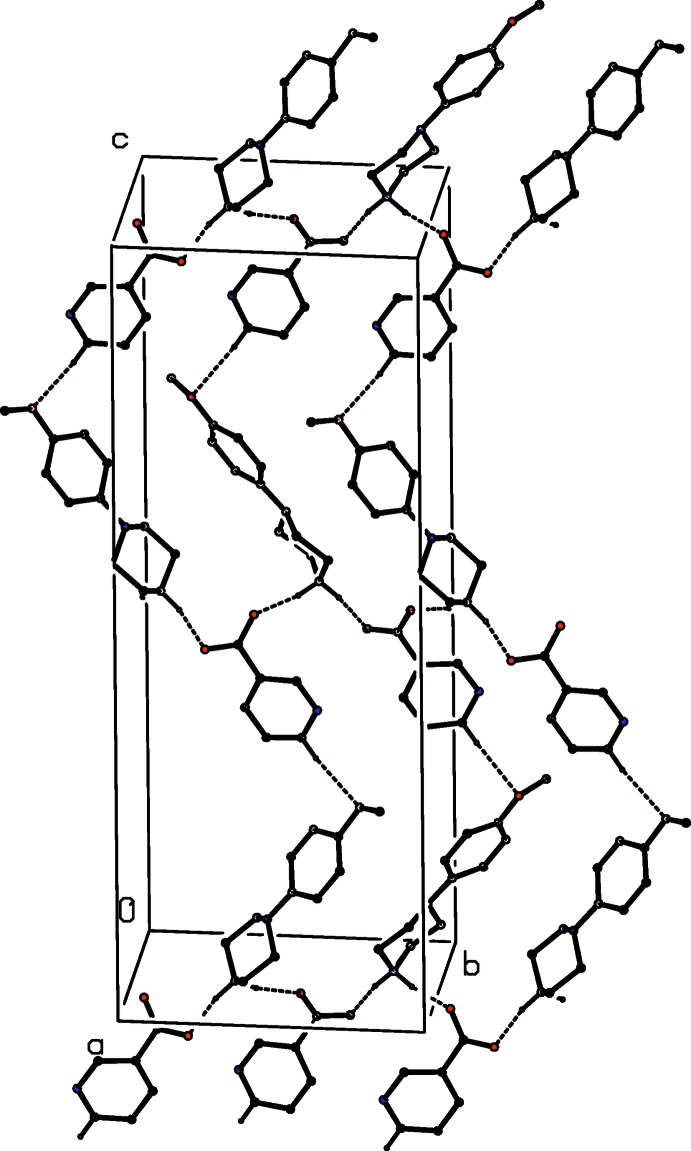
Part of the crystal structure of compound (VI) showing the formation of a sheet of 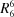
(40) rings lying parallel to (100). Hydrogen bonds are drawn as dashed lines and, for the sake of clarity, the H atoms not involved in the motifs shown have been omitted.

**Figure 17:**
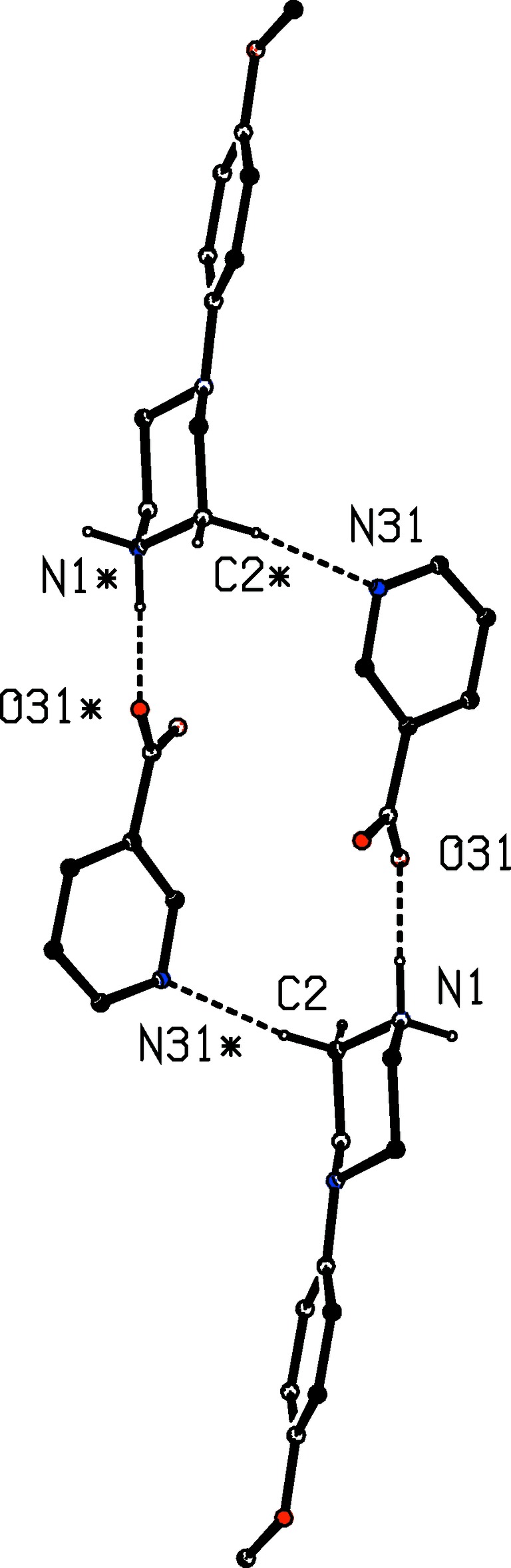
Part of the crystal structure of compound (VI) showing the formation of the 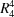
(18) ring which links the (100) sheets. Hydrogen bonds are drawn as dashed lines and, for the sake of clarity, the unit-cell outline and the H atoms which are bonded to the C atoms not involved in the motif shown have been omitted. The atoms marked with an asterisk (*) are at the symmetry position (1 − *x*, 2 − *y*, 1 − *z*).

**Figure 18:**
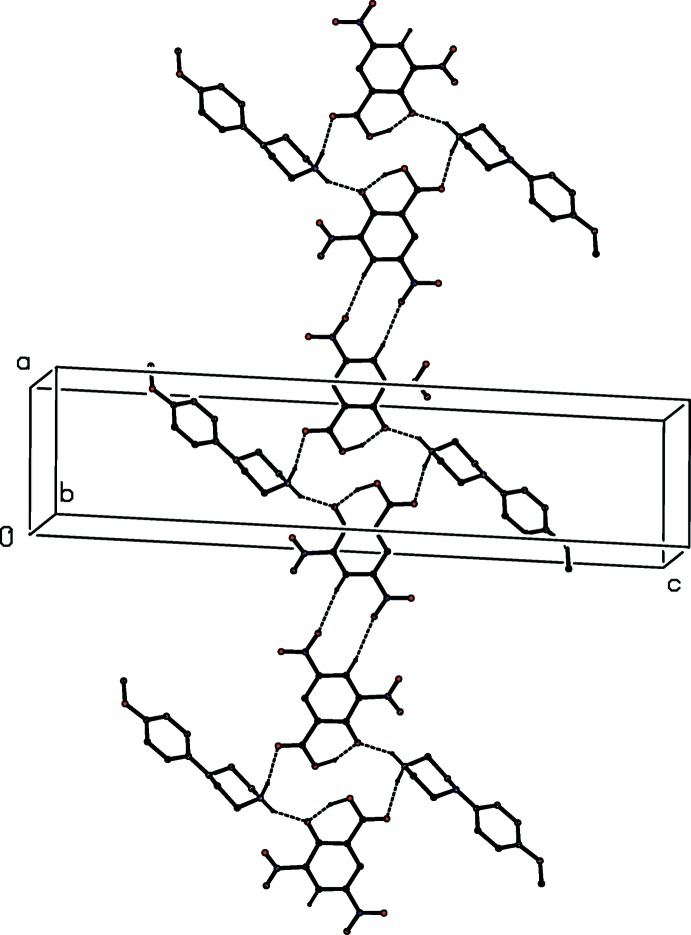
Part of the crystal structure of compound (VII) showing the formation of a chain of 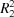
(10) and 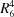
(16) rings parallel to the [210] direction. Hydrogen bonds are drawn as dashed lines and, for the sake of clarity, the H atoms which are bonded to the C atoms not involved in the motif shown have been omitted.

**Figure 19:**
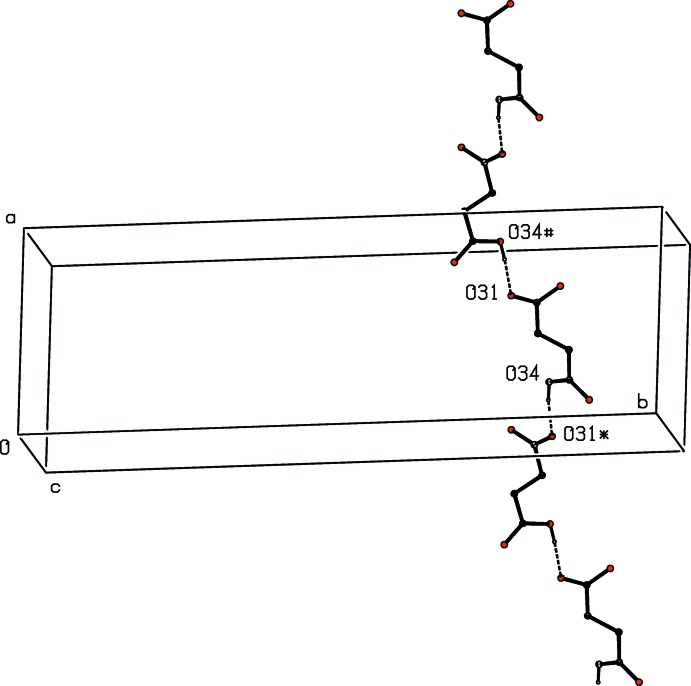
Part of the crystal structure of compound (VIII) showing the formation of a *C*(7) chain of anions, parallel to [10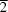]. Hydrogen bonds are drawn as dashed lines and, for the sake of clarity, the H atoms bonded to C atoms have been omitted. The atoms marked with an asterisk (*) or a hash (#) are at the symmetry positions (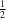 + *x*, 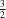 − *y*, −1 + *z*) and (−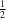 + *x*, 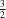 − *y*, 1 + *z*), respectively.

**Figure 20:**
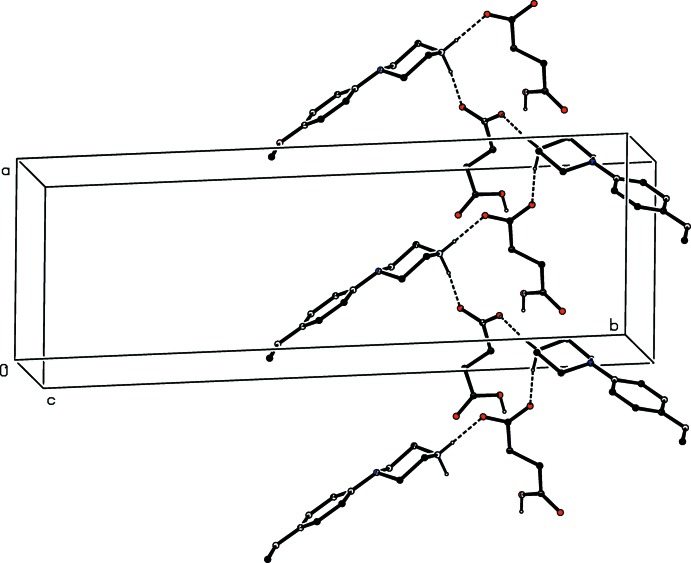
Part of the crystal structure of compound (VIII) showing the formation of a 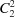
(6) chain parallel to [100]. Hydrogen bonds are drawn as dashed lines and, for the sake of clarity, the H atoms bonded to C atoms have been omitted.

**Figure 21:**
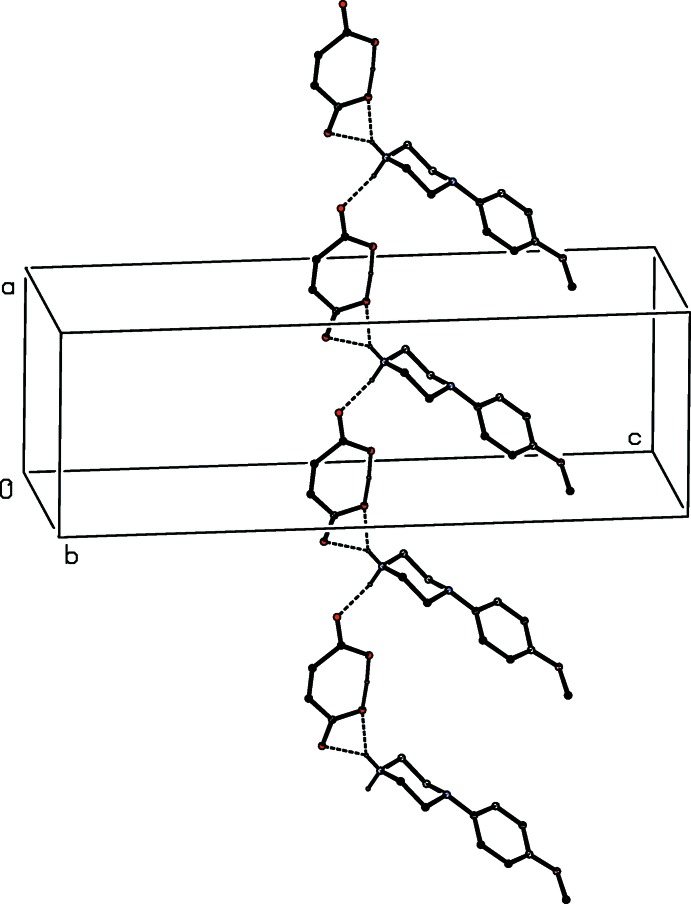
Part of the crystal structure of compound (X) showing the formation of a chain of rings parallel to [100]. Hydrogen bonds are drawn as dashed lines and, for the sake of clarity, the H atoms bonded to C atoms have been omitted.

**Figure 22:**
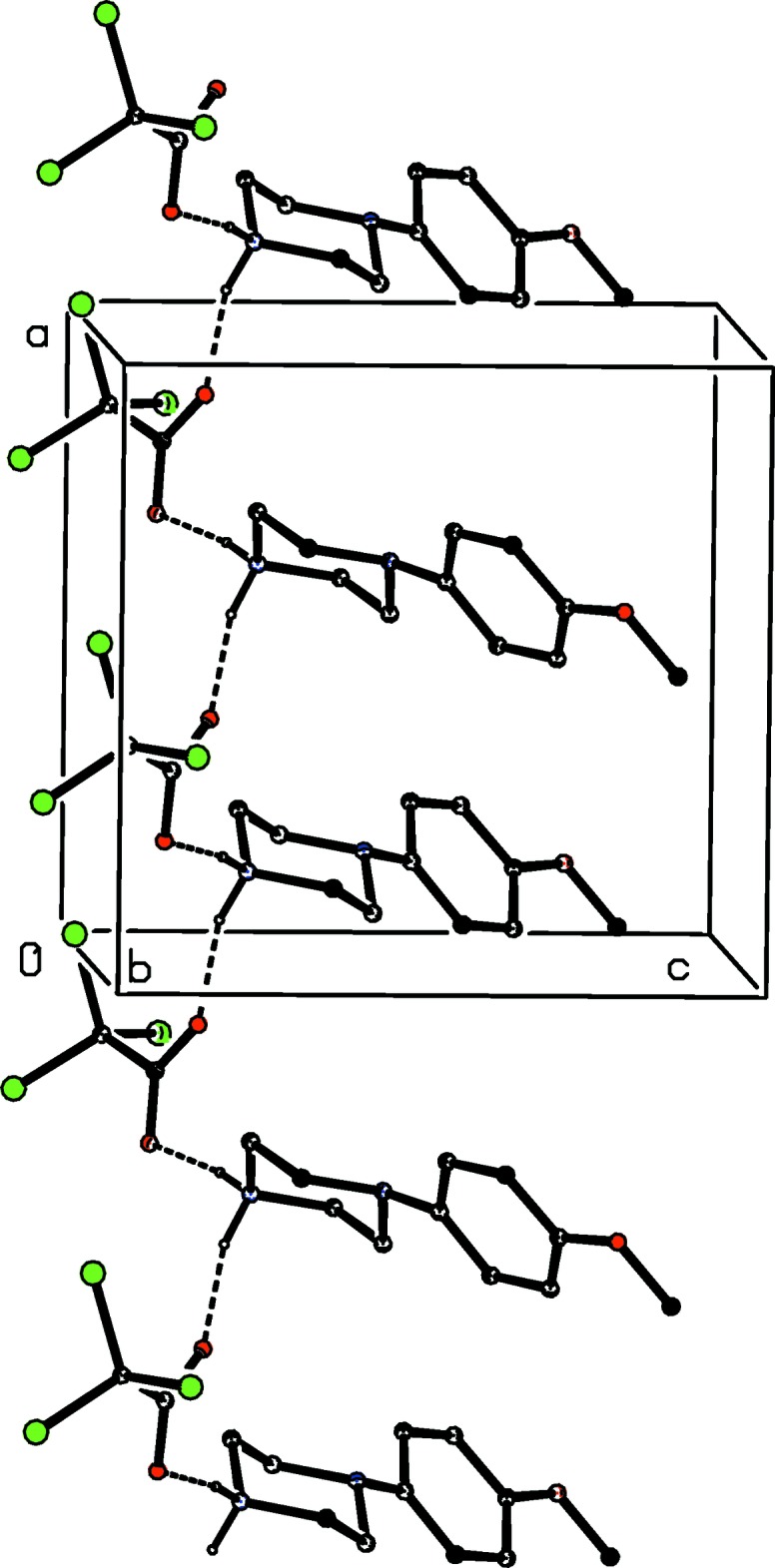
Part of the crystal structure of compound (XI) showing the formation of a 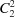
(6) chain parallel to [100]. Hydrogen bonds are drawn as dashed lines and, for the sake of clarity, the H atoms bonded to C atoms have been omitted.

**Figure 23:**
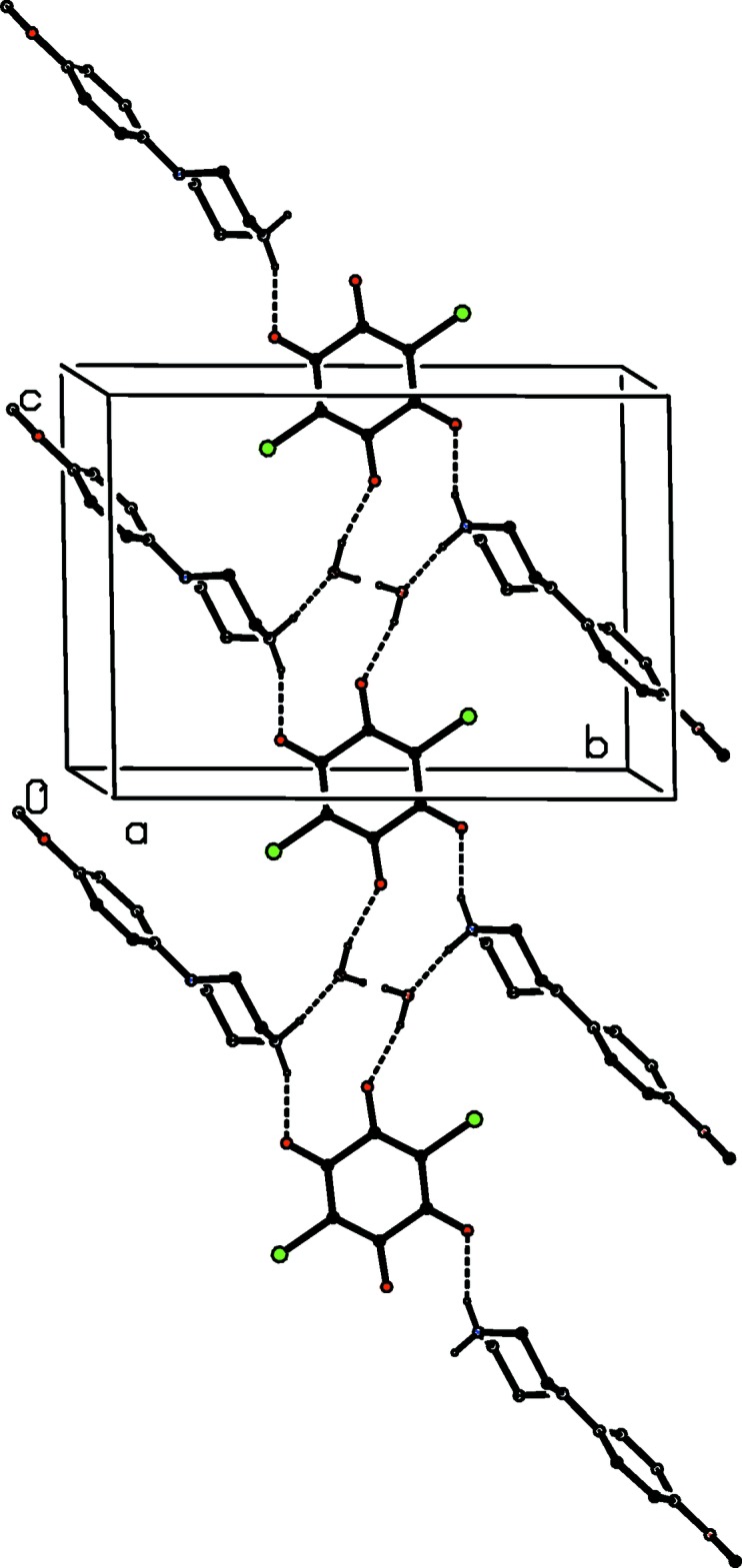
Part of the crystal structure of compound (XII) showing the formation of an 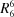
(18) chain of rings parallel to [001]. Hydrogen bonds are drawn as dashed lines and, for the sake of clarity, the H atoms bonded to C atoms have been omitted.

**Figure 24:**
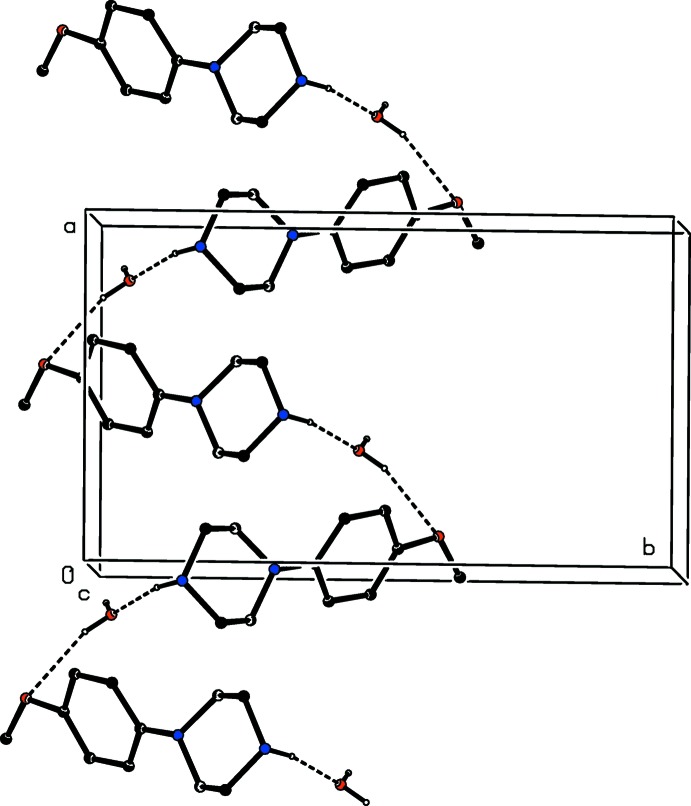
Part of the crystal structure of compound (XII) showing the formation of a 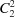
(12) chain of cations and water mol­ecules parallel to [101]. Hydrogen bonds are drawn as dashed lines and, for the sake of clarity, the H atoms bonded to C atoms have been omitted.

Overall, therefore, the hydrogen-bonded assembly is one-dimensional in each of compounds (X) and (XI), two-dimensional in compounds (I)–(V) and (XII), and three-dimensional in compounds (VI)–(IX). Sub-structures in the form of chains of rings can be identified in compounds (I)–(IV) and in (VII), although (I)–(IV) are all monohydrates, while (VII) is solvent free: within the chain of rings formed by (I)–(IV) it is possible to identify a 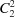
(6) motif formed by water mol­ecules and anions only (Fig. 14 ▸), and a 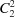
(6) motif built from alternating cations and anions can, in fact, be identified in each of compounds (V), (VI), (VIII), (IX) and (XI) (Figs. 15 ▸, 16 ▸, 20 ▸, 22 ▸). By contrast, a 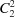
(12) motif, built from water mol­ecules and cations can be identified in the structure of compound (XII) (Fig. 24 ▸), but sub-structural motifs in the form of simple chains are uncommon in this series (Fig. 19 ▸).

## Database survey   

Compounds (I)–(IV), reported here, are isomorphous across the series of anions 4-*X*C_6_H_4_COO^−^, where *X* = H, F, Cl or Br, despite the rather disparate sizes of the substituents *X*. A similar, but more extreme, series of isomorphous salts was found in the substituted anilinium 5-nitro­(hydrogenphthalate) salts (4-*X*C_6_H_4_NH_3_)^+^·(C_8_H_4_NO_6_)^−^, which are isomorphous for *X* = H, Cl, Br and I (Glidewell *et al.*, 2005 ▸). The structures of a number of salts containing the chloranilate dianion have been reported (Ishida, 2004*a* ▸,*b* ▸,*c* ▸,*d* ▸; Sovago *et al.*, 2016 ▸), and the geometric features previously observed in this anion are fully consistent with the geometry found here in (XII): the nature of the electronic delocalization has been confirmed in several such salts using a combination of deformation density plots and net atomic charge calculations (Sovago *et al.*, 2016 ▸).

The structures of very few salts containing the 4-(meth­oxy­phen­yl)piperazin-1-ium cations have been reported. In 4-(4-meth­oxy­phen­yl)piperazin-1-ium chloride, two N—H⋯Cl hydrogen bonds link the ions into 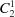
(4) chains (Zia-ur-Rehman *et al.*, 2009 ▸), and in the closely related 4-(4-nitro­phen­yl)piperazin-1-ium chloride monohydrate, a combination of N—H⋯O, O—H⋯Cl and N—H⋯Cl hydrogen bonds links the components into complex ribbons in which each anion accepts three hydrogen bonds (Lu, 2007 ▸). The structure of 4-(3-meth­oxy­phen­yl)piperazin-1-ium maleate has been reported (Verdonk *et al.*, 1997 ▸), as have those of the picrate (Verdonk *et al.*, 1997 ▸) and 6-chloro-5-isopropyl-2,4-dioxopyrimidin-1-ide (Al-Omary *et al.*, 2014 ▸) salts of the 4-(2-meth­oxy­phen­yl)piperazin-1-ium cation. Finally we note, in addition to the 1-aroyl-4-(4-meth­oxy­phen­yl)piperazines referred to in Section 1 above (Kiran Kumar *et al.*, 2019 ▸), the structure of 1-acetyl-(4-hy­droxy­phen­yl)piperazine (Kavitha *et al.*, 2013 ▸), which is an *N*-acetyl­ated derivative of 4-(4-hy­droxy­phen­yl)piperazines, a metabolite of 4-(4-meth­oxy­phen­yl)piperazine.

## Synthesis and crystallization   

All reagents were obtained commercially and were used as received. For the synthesis of each of compounds (I)–(XII), equimolar qu­anti­ties (0.52 mmol of each component) of *N*-(4-meth­oxy­phen­yl)piperazine and the appropriate acid were separately dissolved in methanol (10 ml) and the two solutions were then mixed, stirred briefly, and then set aside to crystallize, giving the solid products (I)–(XII) after a few days. The products were all collected by filtration and then dried in air. Yields (I) 81%, (II) 83%, (III) 83%, (IV) 81%, (V) 83%, (VI) 78%, (VII) 80%, (VIII) 82%, (IX) 82%, (X) 84%, (XI) 79%, (XII) 82%: melting ranges (I) 513–515 K, (II) 405–407 K, (III) 449–451 K, (IV) 447–449 K, (V) 471–473 K, (VI) 441–443 K, (VII) 475–477 K, (VIII) 439–441 K, (IX) 483–485 K, (X) 429–431 K, (XI) 393–395 K, (XII) 575–577 K. Spectroscopic data (IR and ^1^H NMR) are provided in the supporting information. Crystals of compounds (I), (II), and (VIII)–(XII) suitable for single-crystal X-ray diffraction analysis were selected directly from the prepared samples. Crystals of compounds (III)–(VII) suitable for single-crystal X-ray diffraction analysis were grown by slow evaporation, at ambient temperature and in the presence of air, of solutions in methanol–ethyl acetate (initial composition 1:1, *v*/*v*). A number of other acids were used in similar co-crystallization experiments but they did not provide crystal suitable for single-crystal X-ray diffraction, thus: 2- and 3-fluoro­benzoic acids [*cf*. compound (II)], 2- and 3-chloro­benzoic acids [*cf*. compound (III)], 2- and 3-bromo­benzoic acids [*cf*. compound (IV)], 2- and 3-iodo­benzoic acids, phthalic acid, 3-methyl­benzoic acid [*cf*. compound (I)], 2,4-di­chloro­benzoic acid, crotonic and adipic acids [*cf*. compounds (VIII)–(X)], and ascorbic, aspartic and glutamic acids.

## Refinement   

Crystal data, data collection and structure refinement details are summarized in Table 2 ▸. In each of the isomorphous compounds (I)–(IV), the 4-meth­oxy­phenyl group exhibits disorder over two sets of atomic sites, and in each of (VIII) and (IX), the anion exhibits disorder involving two sets of atomic sites having unequal occupancies. In each case, the bonded distances and the 1,3 non-bonded distances in the minor disorder component were restrained to be the same as the equivalent distances in the major disorder component, subject to s.u. values of 0.01 and 0.02 Å, respectively, and the anisotropic displacement parameters for pairs of partial-occupancy atoms occupying essentially the same physical space were constrained to be equal: in addition, it was found necessary to constrain the minor component of the carboxyl group in (IX) to be planar. The ratio of observed-to-unique data was only 39% for compounds (II) and (III): this is probably a consequence of the ambient temperature data collection allied to the disorder: in both (VII) and (IX), the average *U*_3_/*U*_1_ ratio was > 4.0: this may be consequence of the disorder. Apart from those in the minor disorder components of (I)–(IV), (VIII) and (IX), all H atoms were located in difference maps. The H atoms bonded to C atoms were then treated as riding atoms in geometrically idealized positions with C—H distances of 0.93 Å (alkenyl and aromatic), 0.96 Å (CH_3_) or 0.97 Å (CH_2_), and with *U*_iso_(H) = *kU*_eq_(C), where *k* = 1.5 for the methyl groups which were permitted to rotate but not to tilt, and 1.2 for all other H atoms bonded to C atoms: the H atoms bonded to C atoms in the minor disorder components were included on the same basis. The H atoms bonded to O atoms in the disordered components of (VIII) and (IX) were treated as riding atoms with O—H = 0.82 Å and *U*_iso_(H) = 1.5*U*_eq_(O), For the H atoms bonded to N atoms, and for the H atoms bonded to O atoms in (I)–(V), (VII), (X) and (XII), the atomic coordinates were refined with *U*_iso_(H) = 1.2*U*_eq_(N) or 1.5*U*_eq_(O), leading to the N—H and O—H distances shown in Table 1 ▸. The refined occupancies for the disorder components were 0.66 (2) and 0.34 (2) in (I), 0.81 (3) and 0.19 (3) in (II), 0.73 (2) and 0.27 (2) in (III), 0.80 (2) and 0.20 (2) in (IV), 0.660 (15) and 0.340 (15) in (VIII), and 0.906 (9) and 0.094 (9) in (IX). For compound (XI), the correct orientation of the structure relative to the polar axis direction was established using the Flack *x* parameter (Flack, 1983 ▸), *x* = 0.11 (7). However, for compounds (V), (VIII) and (IX), where there is very little resonant scattering the values of the Flack *x* parameter were indeterminate (Flack & Bernardinelli, 2000 ▸), with values −0.3 (5), −0.6 (7) and −0.3 (4), respectively: hence in these three cases, the correct orientation of the structure with respect to the polar axis direction cannot be established, although this has no chemical significance. The refinement of (XII) was treated as a non-merohedral twin, with twin matrix (−1, 0, 0/0, −1, 0/0.496, 0, 1) and with refined twin fractions 0.2467 (9) and 0.7533 (9).

## Supplementary Material

Crystal structure: contains datablock(s) global, I, II, III, IV, V, VI, VII, VIII, IX, X, XI, XII. DOI: 10.1107/S2056989019012702/ex2024sup1.cif

Structure factors: contains datablock(s) I. DOI: 10.1107/S2056989019012702/ex2024Isup2.hkl

Structure factors: contains datablock(s) II. DOI: 10.1107/S2056989019012702/ex2024IIsup3.hkl

Structure factors: contains datablock(s) III. DOI: 10.1107/S2056989019012702/ex2024IIIsup4.hkl

Structure factors: contains datablock(s) IV. DOI: 10.1107/S2056989019012702/ex2024IVsup5.hkl

Structure factors: contains datablock(s) V. DOI: 10.1107/S2056989019012702/ex2024Vsup6.hkl

Structure factors: contains datablock(s) VI. DOI: 10.1107/S2056989019012702/ex2024VIsup7.hkl

Structure factors: contains datablock(s) VII. DOI: 10.1107/S2056989019012702/ex2024VIIsup8.hkl

Structure factors: contains datablock(s) VIII. DOI: 10.1107/S2056989019012702/ex2024VIIIsup9.hkl

Structure factors: contains datablock(s) IX. DOI: 10.1107/S2056989019012702/ex2024IXsup10.hkl

Structure factors: contains datablock(s) X. DOI: 10.1107/S2056989019012702/ex2024Xsup11.hkl

Structure factors: contains datablock(s) XI. DOI: 10.1107/S2056989019012702/ex2024XIsup12.hkl

Structure factors: contains datablock(s) XII. DOI: 10.1107/S2056989019012702/ex2024XIIsup13.hkl

Supporting information file. DOI: 10.1107/S2056989019012702/ex2024Isup14.cml

Supporting information file. DOI: 10.1107/S2056989019012702/ex2024IIsup15.cml

Supporting information file. DOI: 10.1107/S2056989019012702/ex2024IIIsup16.cml

Supporting information file. DOI: 10.1107/S2056989019012702/ex2024IVsup17.cml

Supporting information file. DOI: 10.1107/S2056989019012702/ex2024Vsup18.cml

Supporting information file. DOI: 10.1107/S2056989019012702/ex2024VIsup19.cml

Supporting information file. DOI: 10.1107/S2056989019012702/ex2024VIIsup20.cml

Supporting information file. DOI: 10.1107/S2056989019012702/ex2024XIsup21.cml

CCDC references: 1953078, 1953077, 1953076, 1953075, 1953074, 1953073, 1953072, 1953071, 1953070, 1953069, 1953068, 1953067

Additional supporting information:  crystallographic information; 3D view; checkCIF report

## Figures and Tables

**Figure 2:**
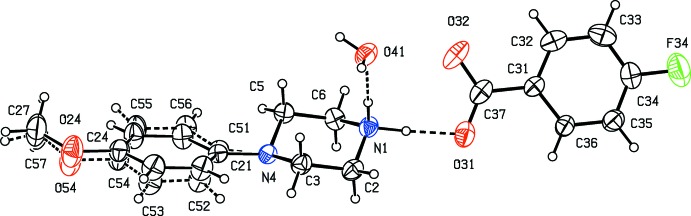
The independent components of compound (II), showing the atom-labelling scheme, the disorder of the 4-meth­oxy­phenyl group, and the hydrogen bonds within the selected asymmetric unit. The major disorder component is drawn using full lines and the minor disorder component is drawn using dashed lines. Displacement ellipsoids are drawn at the 30% probability level and, for the sake of clarity, a few of the atom labels have been omitted.

**Figure 3:**
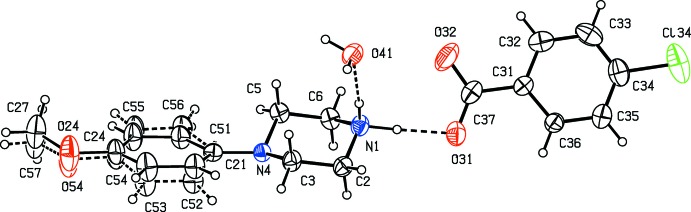
The independent components of compound (III), showing the atom-labelling scheme, the disorder of the 4-meth­oxy­phenyl group, and the hydrogen bonds within the selected asymmetric unit. The major disorder component is drawn using full lines and the minor disorder component is drawn using dashed lines. Displacement ellipsoids are drawn at the 30% probability level and, for the sake of clarity, a few of the atom labels have been omitted.

**Figure 4:**
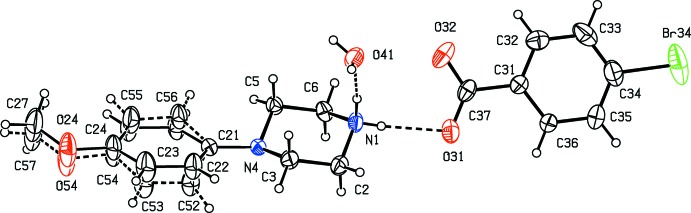
The independent components of compound (IV), showing the atom-labelling scheme, the disorder of the 4-meth­oxy­phenyl group, and the hydrogen bonds within the selected asymmetric unit. The major disorder component is drawn using full lines and the minor disorder component is drawn dashed broken lines. Displacement ellipsoids are drawn at the 30% probability level and, for the sake of clarity, a few of the atom labels have been omitted.

**Figure 9:**
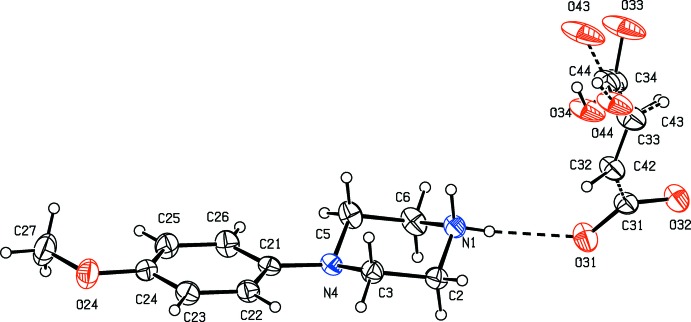
The independent components of compound (IX), showing the atom-labelling scheme, the disorder of anion, and the hydrogen bonds within the selected asymmetric unit. The major disorder component is drawn using full lines and the minor disorder component is drawn using dashed lines. Displacement ellipsoids are drawn at the 30% probability level.

**Figure 10:**
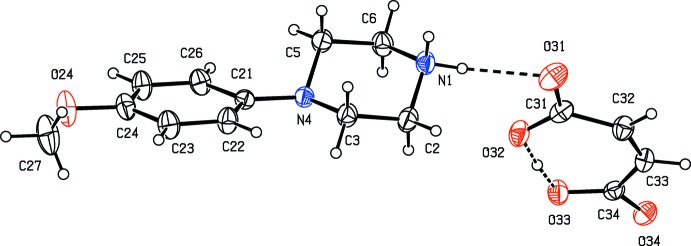
The independent components of compound (X), showing the atom-labelling scheme and the hydrogen bonds within the selected asymmetric unit. Displacement ellipsoids are drawn at the 30% probability level.

**Figure 11:**
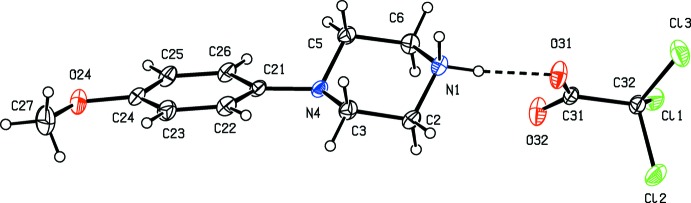
The independent components of compound (XI), showing the atom-labelling scheme and the hydrogen bond within the selected asymmetric unit. Displacement ellipsoids are drawn at the 30% probability level.

**Table 1:** Hydrogen-bond parameters and short inter­molecular contacts (Å, °) *Cg*1 and*Cg*2 are the centroids of the C31–C36 and C21–C26 rings, respectively.

Compound	*D*—H⋯*A*	*D*—H	H⋯*A*	*D*⋯*A*	*D*—H⋯*A*
(I)	N1—H11⋯O31	0.90 (2)	1.88 (2)	2.777 (3)	174.1 (19)
	N1—H12⋯O41	0.97 (2)	1.85 (2)	2.808 (3)	169.7 (18)
	O41—H41⋯O32^i^	0.88 (3)	1.75 (3)	2.631 (3)	177 (3)
	O41—H42⋯O31^ii^	0.91 (3)	1.87 (3)	2.763 (3)	169 (3)
	C2—H2*B*⋯O31^iii^	0.97	2.54	3.485 (3)	165
	C22—H22⋯*Cg*1^ii^	0.93	2.85	3.603 (3)	139
	C26—H26⋯*Cg*1^iv^	0.93	2.90	3.62 (2)	135
	C56—H56⋯*Cg*1^iv^	0.93	2.64	3.41 (5)	141
					
(II)	N1—H11⋯O31	1.09 (3)	1.67 (3)	2.758 (4)	174.1 (19)
	N1—H12⋯O41	0.86 (3)	1.96 (3)	2.818 (4)	170 (3)
	O41—H41⋯O32^i^	0.86 (4)	1.75 (4)	2.627 (4)	174 (4)
	O41—H42⋯O31^ii^	0.91 (4)	1.88 (4)	2.768 (3)	163 (3)
	C2—H2*B*⋯O31^iii^	0.97	2.58	3.529 (4)	166
	C6—H6*B*⋯O41^i^	0.97	2.57	3.386 (4)	142
	C26—H26⋯*Cg*1^iv^	0.93	2.81	3.56 (2)	138
	C56—H56⋯*Cg*1^iv^	0.93	2.96	3.55 (9)	123
					
(III)	N1—H11⋯O31	1.09 (3)	1.71 (3)	2.790 (4)	176 (3)
	N1—H12⋯O41	0.83 (3)	1.98 (3)	2.811 (4)	174 (3)
	O41—H41⋯O32^i^	0.91 (4)	1.73 (4)	2.624 (4)	172 (4)
	O41—H42⋯O31^ii^	0.94 (4)	1.84 (4)	2.775 (4)	170 (4)
	C2—H2*B*⋯O31^iii^	0.97	2.52	3.467 (4)	165
	C6—H6*B*⋯O41^i^	0.97	2.60	3.408 (4)	141
	C22—H22⋯*Cg*1^iv^	0.93	2.89	3.631 (13)	137
	C26—H26⋯*Cg*1^iv^	0.93	2.81	3.58 (2)	141
					
(IV)	N1—H11⋯O31	0.78 (4)	2.03 (4)	2.805 (5)	174 (5)
	N1—H12⋯O41	0.95 (5)	1.86 (5)	2.802 (5)	172 (4)
	O41—H41⋯O32^i^	0.79 (6)	1.84 (6)	2.623 (6)	170 (6)
	O41—H42⋯O31^ii^	0.79 (7)	2.00 (7)	2.772 (5)	169 (6)
	C2—H2*B*⋯O31^iii^	0.97	2.52	3.471 (5)	166
	C22—H22⋯*Cg*1^ii^	0.93	2.52	3.471 (5)	166
	C26—H26⋯*Cg*1^iv^	0.93	2.84	3.58 (2)	137
					
(V)	N1—H11⋯O31	0.96 (3)	1.85 (3)	2.759 (3)	156 (3)
	N1—H11⋯O32	0.96 (3)	2.47 (3)	3.283 (3)	142 (2)
	N1—H12⋯O32^v^	0.95 (3)	1.87 (3)	2.806 (3)	166 (2)
	O33—H33*A*⋯O31	0.97 (3)	1.60 (3)	2.516 (3)	156 (3)
	C6—H6*A*⋯O33^vi^	0.97	2.58	3.444 (3)	148
	C2—H2*A*⋯*Cg*1^vii^	0.97	2.88	3.711 (3)	144
	C26—H26⋯*Cg*1^viii^	0.93	2.87	3.642 (3)	141
					
(VI)	N1—H11⋯O31	0.976 (19)	1.714 (19)	2.677 (2)	168.2 (18)
	N1—H12⋯O32^ix^	0.94 (2)	1.82 (2)	2.749 (2)	168.3 (17)
	C2—H2*B*⋯N31^iv^	0.97	2.56	3.518 (2)	169
	C36—H36⋯O24^*x*^	0.93	2.51	3.432 (2)	172
	C3—H3*A*⋯*Cg*1^xi^	0.97	2.97	3.775 (2)	156
					
(VII)	O32—H32⋯O33	1.04 (4)	1.47 (4)	2.472 (3)	158 (3)
	N1—H11⋯O33	0.93 (3)	1.98 (3)	2.020 (3)	150 (3)
	N1—H11⋯O34	0.93 (3)	2.27 (3)	2.910 (3)	126 (2)
	N1—H12⋯O31^i^	0.93 (3)	2.04 (3)	2.931 (3)	160 (3)
	N1—H12⋯O32^i^	0.93 (3)	2.58 (3)	3.250 (3)	129 (2)
	C34—H34⋯O36^xii^	0.93	2.53	3.449 (3)	171
	C5—H5*B*⋯*Cg*2^xiii^	0.97	2.84	3.639 (3)	140
					
(VIII)	N1—H11⋯O31	0.86 (3)	1.90 (3)	2.750 (15)	167 (4)
	N1—H12⋯O32^xiv^	0.98 (3)	1.77 (4)	2.741 (19)	171 (3)
	O34—H34⋯O31^xv^	0.82	1.79	2.60 (2)	168
	N1—H11⋯O41	0.86 (3)	2.18 (4)	3.03 (3)	165 (4)
	N1—H12⋯O42^xiv^	0.98 (3)	1.82 (5)	2.77 (4)	163 (3)
	O44—H44⋯O41^xv^	0.82	1.56	2.35 (2)	161
	C3—H3*A*⋯*Cg*2^xvi^	0.97	2.76	3.652 (3)	154
					
(IX)	N1—H11⋯O31	0.81 (4)	2.18 (3)	2.940 (4)	155 (3)
	N1—H12⋯O32^xiv^	0.96 (4)	1.77 (4)	2.714 (4)	169 (3)
	O34—H34⋯O31^xv^	0.82	1.71	2.522 (5)	170
	O43—H34⋯O31^xv^	0.82	1.62	2.44 (2)	175
	C3—H3*A*⋯*Cg*2^xvi^	0.97	2.76	3.650 (3)	153
					
(*X*)	O33—H33*A*⋯O32	1.167 (18)	1.247 (18)	2.4121 (16)	175 (2)
	N1—H11⋯O31	0.915 (17)	2.126 (16)	2.9309 (19)	146.2 (15)
	N1—H11⋯O32	0.915 (17)	2.296 (17)	3.0798 (18)	143.5 (14)
	N1—H12⋯O34^xvii^	0.919 (18)	1.881 (18)	2.7563 (17)	158.5 (17)
	C2—H2*A*⋯O34^ii^	0.97	2.56	3.363 (2)	140
					
(XI)	N1—H11⋯O31	0.92 (4)	1.86 (4)	2.775 (4)	172 (3)
	N1—H11⋯O32^xviii^	0.97 (3)	1.80 (3)	2.724 (3)	158 (3)
					
(XII)	N1—H11⋯O31	0.89 (3)	1.96 (3)	2.802 (3)	157 (2)
	N1—H11⋯O33	0.89 (3)	2.29 (2)	2.838 (3)	119 (2)
	N1—H12⋯O41	0.90 (2)	1.92 (2)	2.798 (3)	168 (3)
	O41—H41⋯O33^i^	0.84 (4)	1.92 (4)	2.738 (3)	166 (3)
	O41—H42⋯O24^xix^	0.82 (3)	2.49 (3)	3.269 (3)	160 (3)

Symmetry codes: (i) 1 − *x*, 1 − *y*, 1 − *z*; (ii) 2 − *x*, 1 − *y*, 1 − *z*; (iii) 2 − *x*, 2 − *y*, 1 − *z*; (iv) 1 − *x*, 2 − *y*, 1 − *z*; (v) 1 − *x*, −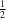 + *y*, 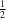 − *z*; (vi) 2 − *x*, −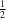 + *y*, 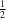 − *z*; (vii) 2 − *x*, 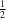 + *y*, 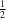 − *z*; (viii) 1 − *x*, 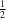 + *y*, 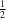 − *z*; (ix) 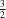 − *x*, −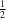 + *y*, *z*; (*x*) *x*, 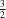 − *y*, 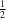 + *z*; (xi) −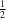 + *x*, 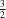 − *y*, 1 − *z*; (xii) 3 − *x*, 2 − *y*, 1 − *z*; (xiii) 1 − *x*, −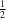 + *y*, 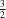 − *z*; (xiv) −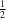 + *x*, 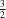 − *y*, *z*; (xv) −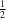 + *x*, 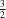 − *y*, 1 + *z*; (xvi) 1 − *x*, 1 − *y*, 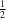 + *z*; (xvii) −1 + *x*, *y*, *z*; (xviii) −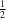 + *x*, 1 − *y*, *z*; (xix) −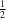 + *x*, 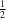 − *y*, −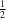 + *z*.

**Table:** 

	(I)	(II)	(III)	(IV)
Crystal data				
Chemical formula	C_11_H_17_N_2_O^+^·C_7_H_5_O_2_^−^·H_2_O	C_11_H_17_N_2_O^+^·C_7_H_4_FO_2_^−^·H_2_O	C_11_H_17_N_2_O^+^·C_7_H_4_ClO_2_^−^·H_2_O	C_11_H_17_N_2_O^+^·C_7_H_4_BrO_2_^−^·H_2_O
*M*_r_	332.39	350.38	366.83	411.28
Crystal system, space group	Triclinic, *P* 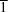	Triclinic, *P* 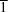	Triclinic, *P* 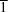	Triclinic, *P* 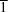
Temperature (K)	296	293	293	293
*a*, *b*, *c* (Å)	6.215 (1), 7.547 (1), 18.716 (4)	6.256 (1), 7.489 (1), 19.097 (2)	6.211 (1), 7.481 (1), 20.144 (4)	6.2004 (8), 7.4957 (9), 20.440 (2)
α, β, γ (°)	84.34 (2), 87.14 (2), 84.69 (2)	84.19 (1), 86.98 (2), 84.62 (2)	84.90 (2), 87.48 (2), 85.19 (2)	85.08 (1), 87.37 (1), 85.00 (1)
*V* (Å^3^)	869.1 (3)	885.4 (2)	928.4 (3)	942.17 (19)
*Z*	2	2	2	2
Radiation type	Mo *K*α	Mo *K*α	Mo *K*α	Mo *K*α
μ (mm^−1^)	0.09	0.10	0.23	2.21
Crystal size (mm)	0.40 × 0.24 × 0.04	0.40 × 0.24 × 0.04	0.20 × 0.16 × 0.02	0.48 × 0.44 × 0.16

Data collection				
Diffractometer	Oxford Diffraction Xcalibur with Sapphire CCD	Oxford Diffraction Xcalibur with Sapphire CCD	Oxford Diffraction Xcalibur with Sapphire CCD	Oxford Diffraction Xcalibur with Sapphire CCD
Absorption correction	Multi-scan (*CrysAlis RED*; Oxford Diffraction, 2009 ▸)	Multi-scan (*CrysAlis RED*; Oxford Diffraction, 2009 ▸)	Multi-scan (*CrysAlis RED*; Oxford Diffraction, 2009 ▸)	Multi-scan (*CrysAlis RED*; Oxford Diffraction, 2009 ▸)
*T*_min_, *T*_max_	0.834, 0.996	0.973, 0.996	0.951, 0.995	0.536, 0.719
No. of measured, independent and observed [*I* > 2σ(*I*)] reflections	5751, 3442, 1839	5760, 3477, 1355	5883, 3454, 1343	6176, 3818, 2063
*R*_int_	0.029	0.046	0.041	0.018
(sin θ/λ)_max_ (Å^−1^)	0.618	0.618	0.607	0.629

Refinement				
*R*[*F* ^2^ > 2σ(*F* ^2^)], *wR*(*F* ^2^), *S*	0.054, 0.134, 1.02	0.066, 0.128, 1.01	0.065, 0.135, 0.94	0.068, 0.197, 1.06
No. of reflections	3442	3477	3454	3818
No. of parameters	256	265	265	265
No. of restraints	17	17	17	17
H-atom treatment	H atoms treated by a mixture of independent and constrained refinement	H atoms treated by a mixture of independent and constrained refinement	H atoms treated by a mixture of independent and constrained refinement	H atoms treated by a mixture of independent and constrained refinement
Δρ_max_, Δρ_min_ (e Å^−3^)	0.17, −0.19	0.13, −0.14	0.24, −0.23	0.94, −0.64

**Table:** 

	(V)	(VI)	(VII)	(VIII)
Crystal data				
Chemical formula	C_11_H_17_N_2_O^+^·C_7_H_5_O_3_^−^	C_11_H_17_N_2_O^+^·C_6_H_4_NO_2_^−^	C_7_H_3_N_2_O_7_^+^·C_11_H_17_N_2_O^−^	C_11_H_17_N_2_O^+^·C_4_H_5_O_4_^−^
*M*_r_	330.38	315.37	420.38	310.35
Crystal system, space group	Orthorhombic, *P*2_1_2_1_2_1_	Orthorhombic, *P* *b* *c* *a*	Monoclinic, *P*2_1_/*c*	Orthorhombic, *P* *n* *a*2_1_
Temperature (K)	296	296	296	296
*a*, *b*, *c* (Å)	6.5009 (8), 7.9735 (9), 32.155 (4)	9.2817 (7), 11.2905 (7), 30.309 (2)	7.5500 (9), 7.6489 (9), 32.719 (6)	9.3225 (9), 28.261 (3), 5.8228 (8)
α, β, γ (°)	90, 90, 90	90, 90, 90	90, 91.30 (1), 90	90, 90, 90
*V* (Å^3^)	1666.8 (3)	3176.2 (4)	1889.0 (5)	1534.1 (3)
*Z*	4	8	4	4
Radiation type	Mo *K*α	Mo *K*α	Mo *K*α	Mo *K*α
μ (mm^−1^)	0.09	0.09	0.12	0.10
Crystal size (mm)	0.42 × 0.42 × 0.34	0.46 × 0.42 × 0.36	0.18 × 0.12 × 0.06	0.44 × 0.42 × 0.24

Data collection				
Diffractometer	Oxford Diffraction Xcalibur with Sapphire CCD	Oxford Diffraction Xcalibur with Sapphire CCD	Oxford Diffraction Xcalibur with Sapphire CCD	Oxford Diffraction Xcalibur with Sapphire CCD
Absorption correction	Multi-scan (*CrysAlis RED*; Oxford Diffraction, 2009 ▸)	Multi-scan (*CrysAlis RED*; Oxford Diffraction, 2009 ▸)	Multi-scan (*CrysAlis RED*; Oxford Diffraction, 2009 ▸)	Multi-scan (*CrysAlis RED*; Oxford Diffraction, 2009 ▸)
*T*_min_, *T*_max_	0.899, 0.969	0.879, 0.968	0.916, 0.993	0.816, 0.976
No. of measured, independent and observed [*I* > 2σ(*I*)] reflections	6249, 3564, 2875	22154, 3593, 2616	8215, 4074, 2003	5828, 2419, 2053
*R*_int_	0.014	0.028	0.038	0.018
(sin θ/λ)_max_ (Å^−1^)	0.656	0.658	0.660	0.649

Refinement				
*R*[*F* ^2^ > 2σ(*F* ^2^)], *wR*(*F* ^2^), *S*	0.041, 0.089, 1.05	0.048, 0.119, 1.04	0.066, 0.128, 1.03	0.043, 0.104, 1.14
No. of reflections	3564	3593	4074	2419
No. of parameters	228	215	281	233
No. of restraints	0	0	0	16
H-atom treatment	H atoms treated by a mixture of independent and constrained refinement	H atoms treated by a mixture of independent and constrained refinement	H atoms treated by a mixture of independent and constrained refinement	H atoms treated by a mixture of independent and constrained refinement
Δρ_max_, Δρ_min_ (e Å^−3^)	0.14, −0.13	0.19, −0.16	0.22, −0.23	0.16, −0.24
Absolute structure	Flack *x* determined using 1011 quotients [(*I* ^+^)−(*I* ^−^)]/[(*I* ^+^)+(*I* ^−^)] (Parsons *et al.*, 2013 ▸)	–	–	Flack *x* determined using 460 quotients [(*I* ^+^)−(*I* ^−^)]/[(*I* ^+^)+(*I* ^−^)] (Parsons *et al.*, 2013 ▸)
Absolute structure parameter	–	–	–	–

**Table:** 

	(IX)	(X)	(XI)	(XII)
Crystal data				
Chemical formula	C_11_H_17_N_2_O^+^·C_4_H_3_O_4_^−^	C_11_H_17_N_2_O^+^·C_4_H_3_O_4_^−^	C_11_H_17_N_2_O^+^·C_2_Cl_3_O_2_^−^	C_11_H_17_N_2_O^+^·0.5C_6_Cl_2_O_4_^2−^·H_2_O
*M*_r_	308.33	308.33	355.64	314.76
Crystal system, space group	Orthorhombic, *P* *n* *a*2_1_	Monoclinic, *P*2_1_/*c*	Orthorhombic, *P* *c* *a*2_1_	Monoclinic, *P*2_1_/*n*
Temperature (K)	296	296	296	296
*a*, *b*, *c* (Å)	9.069 (1), 28.528 (3), 5.8375 (9)	9.063 (1), 6.4956 (9), 26.093 (3)	10.6117 (11), 13.808 (1), 10.9137 (8)	9.1597 (5), 15.1434 (8), 10.8742 (6)
α, β, γ (°)	90, 90, 90	90, 93.18 (1), 90	90, 90, 90	90, 102.067 (5), 90
*V* (Å^3^)	1510.3 (3)	1533.7 (3)	1599.1 (2)	1475.02 (14)
*Z*	4	4	4	4
Radiation type	Mo *K*α	Mo *K*α	Mo *K*α	Mo *K*α
μ (mm^−1^)	0.10	0.10	0.58	0.28
Crystal size (mm)	0.48 × 0.48 × 0.08	0.48 × 0.44 × 0.32	0.48 × 0.48 × 0.20	0.44 × 0.24 × 0.20

Data collection				
Diffractometer	Oxford Diffraction Xcalibur with Sapphire CCD	Oxford Diffraction Xcalibur with Sapphire CCD	Oxford Diffraction Xcalibur with Sapphire CCD	Oxford Diffraction Xcalibur with Sapphire CCD
Absorption correction	Multi-scan (*CrysAlis RED*; Oxford Diffraction, 2009 ▸)	Multi-scan (*CrysAlis RED*; Oxford Diffraction, 2009 ▸)	Multi-scan (*CrysAlis RED*; Oxford Diffraction, 2009 ▸)	Multi-scan (*CrysAlis RED*; Oxford Diffraction, 2009 ▸)
*T*_min_, *T*_max_	0.888, 0.992	0.871, 0.968	0.476, 0.892	0.892, 0.947
No. of measured, independent and observed [*I* > 2σ(*I*)] reflections	5834, 2827, 2316	6112, 3311, 2459	6173, 2428, 2278	9650, 9650, 7444
*R*_int_	0.015	0.014	0.027	?
(sin θ/λ)_max_ (Å^−1^)	0.650	0.651	0.654	0.651

Refinement				
*R*[*F* ^2^ > 2σ(*F* ^2^)], *wR*(*F* ^2^), *S*	0.041, 0.101, 1.05	0.040, 0.111, 1.05	0.032, 0.086, 1.08	0.039, 0.105, 1.02
No. of reflections	2827	3311	2428	9650
No. of parameters	221	210	198	204
No. of restraints	11	0	1	0
H-atom treatment	H atoms treated by a mixture of independent and constrained refinement	H atoms treated by a mixture of independent and constrained refinement	H atoms treated by a mixture of independent and constrained refinement	H atoms treated by a mixture of independent and constrained refinement
Δρ_max_, Δρ_min_ (e Å^−3^)	0.15, −0.14	0.21, −0.13	0.25, −0.31	0.23, −0.32
Absolute structure	Flack *x* determined using 769 quotients [(*I* ^+^)−(*I* ^−^)]/[(*I* ^+^)+(*I* ^−^)] (Parsons *et al.*, 2013 ▸)	–	Classical Flack method preferred over Parsons because s.u. lower	–
Absolute structure parameter	–	–	0.11 (7)	–

Computer programs: *CrysAlis CCD* (Oxford Diffraction, 2009 ▸), *CrysAlis RED* (Oxford Diffraction, 2009 ▸), *SHELXT* (Sheldrick, 2015*a* ▸), *SHELXL2014* (Sheldrick, 2015*b* ▸) and *PLATON* (Spek, 2009 ▸).

## supplementary crystallographic information

### 4-(4-Methoxyphenyl)piperazin-1-ium benzoate monohydrate (I) Crystal data

**Table:**

C_11_H_17_N_2_O^+^·C_7_H_5_O_2_^−^·H_2_O	*Z* = 2
*M**_r_* = 332.39	*F*(000) = 356
Triclinic, *P*1	*D*_x_ = 1.270 Mg m^−^^3^
*a* = 6.215 (1) Å	Mo *K*α radiation, λ = 0.71073 Å
*b* = 7.547 (1) Å	Cell parameters from 3742 reflections
*c* = 18.716 (4) Å	θ = 2.8–28.0°
α = 84.34 (2)°	µ = 0.09 mm^−^^1^
β = 87.14 (2)°	*T* = 296 K
γ = 84.69 (2)°	Plate, colourless
*V* = 869.1 (3) Å^3^	0.40 × 0.24 × 0.04 mm

### 4-(4-Methoxyphenyl)piperazin-1-ium benzoate monohydrate (I) Data collection

**Table:**

Oxford Diffraction Xcalibur with Sapphire CCD diffractometer	3442 independent reflections
Radiation source: Enhance (Mo) X-ray Source	1839 reflections with *I* > 2σ(*I*)
Graphite monochromator	*R*_int_ = 0.029
ω scans	θ_max_ = 26.1°, θ_min_ = 2.8°
Absorption correction: multi-scan (CrysAlis RED; Oxford Diffraction, 2009)	*h* = −7→7
*T*_min_ = 0.834, *T*_max_ = 0.996	*k* = −9→9
5751 measured reflections	*l* = −19→23

### 4-(4-Methoxyphenyl)piperazin-1-ium benzoate monohydrate (I) Refinement

**Table:**

Refinement on *F*^2^	Primary atom site location: difference Fourier map
Least-squares matrix: full	Hydrogen site location: mixed
*R*[*F*^2^ > 2σ(*F*^2^)] = 0.054	H atoms treated by a mixture of independent and constrained refinement
*wR*(*F*^2^) = 0.134	*w* = 1/[σ^2^(*F*_o_^2^) + (0.0611*P*)^2^] where *P* = (*F*_o_^2^ + 2*F*_c_^2^)/3
*S* = 1.02	(Δ/σ)_max_ < 0.001
3442 reflections	Δρ_max_ = 0.17 e Å^−^^3^
256 parameters	Δρ_min_ = −0.19 e Å^−^^3^
17 restraints	

### 4-(4-Methoxyphenyl)piperazin-1-ium benzoate monohydrate (I) Special details

**Table:**

Experimental. Compound (I). IR (KBr , cm^-1^) 3328 (OH), 3002 (H_2_) 2841 (OCH_3_), 1591 (COO). NMR (CDCl_3_) δ(^1^H) 3.22 (m, 4H, piperazine), 3.29 (m, 4H, piperazine), 3.77 (s, 3H, OCH_3_), 6.86 (m, 4H, methoxyphenyl), 7.39 (m, 2H, phenyl), 7.46 (m, 1H, phenyl), 8.05 (m, 2H, phenyl).
Geometry. All esds (except the esd in the dihedral angle between two l.s. planes) are estimated using the full covariance matrix. The cell esds are taken into account individually in the estimation of esds in distances, angles and torsion angles; correlations between esds in cell parameters are only used when they are defined by crystal symmetry. An approximate (isotropic) treatment of cell esds is used for estimating esds involving l.s. planes.

### 4-(4-Methoxyphenyl)piperazin-1-ium benzoate monohydrate (I) Fractional atomic coordinates and isotropic or equivalent isotropic displacement parameters (Å^2^)

**Table:**

	*x*	*y*	*z*	*U*_iso_*/*U*_eq_	Occ. (<1)
N1	0.7874 (3)	0.7435 (3)	0.44989 (10)	0.0482 (5)	
H11	0.834 (3)	0.766 (3)	0.4926 (13)	0.058*	
H12	0.748 (3)	0.621 (3)	0.4555 (11)	0.058*	
C2	0.9747 (3)	0.7530 (3)	0.39809 (12)	0.0519 (6)	
H2A	1.0940	0.6709	0.4157	0.062*	
H2B	1.0215	0.8729	0.3934	0.062*	
C3	0.9144 (3)	0.7056 (3)	0.32596 (12)	0.0497 (6)	
H3A	1.0363	0.7191	0.2921	0.060*	
H3B	0.8837	0.5812	0.3301	0.060*	
N4	0.7262 (2)	0.8168 (2)	0.29814 (9)	0.0394 (4)	
C5	0.5425 (3)	0.8133 (3)	0.35024 (11)	0.0463 (5)	
H5A	0.4932	0.6943	0.3561	0.056*	
H5B	0.4244	0.8957	0.3320	0.056*	
C6	0.6019 (3)	0.8640 (3)	0.42214 (12)	0.0513 (6)	
H6A	0.6397	0.9866	0.4173	0.062*	
H6B	0.4789	0.8557	0.4559	0.062*	
C21	0.677 (2)	0.782 (2)	0.2275 (6)	0.034 (2)	0.66 (2)
C22	0.8134 (13)	0.6810 (17)	0.1831 (5)	0.0450 (18)	0.66 (2)
H22	0.9414	0.6235	0.2007	0.054*	0.66 (2)
C23	0.7627 (13)	0.6649 (18)	0.1132 (5)	0.053 (2)	0.66 (2)
H23	0.8602	0.6005	0.0841	0.064*	0.66 (2)
C24	0.5725 (14)	0.7413 (16)	0.0858 (5)	0.0438 (17)	0.66 (2)
C25	0.438 (2)	0.846 (3)	0.1278 (8)	0.0599 (10)	0.66 (2)
H25	0.3145	0.9097	0.1088	0.072*	0.66 (2)
C26	0.486 (3)	0.858 (3)	0.1984 (8)	0.054 (2)	0.66 (2)
H26	0.3857	0.9191	0.2276	0.064*	0.66 (2)
O24	0.541 (3)	0.720 (3)	0.0149 (7)	0.078 (3)	0.66 (2)
C27	0.335 (3)	0.773 (4)	−0.0122 (11)	0.090 (2)	0.66 (2)
H27A	0.2297	0.7017	0.0128	0.135*	0.66 (2)
H27B	0.3373	0.7580	−0.0626	0.135*	0.66 (2)
H27C	0.2962	0.8970	−0.0053	0.135*	0.66 (2)
C51	0.669 (5)	0.815 (5)	0.2258 (12)	0.034 (2)	0.34 (2)
C52	0.817 (3)	0.735 (2)	0.1784 (11)	0.0450 (18)	0.34 (2)
H52	0.9535	0.6917	0.1937	0.054*	0.34 (2)
C53	0.764 (3)	0.718 (3)	0.1087 (10)	0.053 (2)	0.34 (2)
H53	0.8615	0.6555	0.0790	0.064*	0.34 (2)
C54	0.571 (3)	0.792 (2)	0.0828 (10)	0.0438 (17)	0.34 (2)
C55	0.414 (4)	0.854 (7)	0.1309 (15)	0.0599 (10)	0.34 (2)
H55	0.2702	0.8738	0.1184	0.072*	0.34 (2)
C56	0.474 (5)	0.886 (7)	0.1983 (15)	0.054 (2)	0.34 (2)
H56	0.3803	0.9570	0.2260	0.064*	0.34 (2)
O54	0.528 (6)	0.756 (7)	0.0142 (14)	0.078 (3)	0.34 (2)
C57	0.310 (6)	0.780 (9)	−0.006 (2)	0.090 (2)	0.34 (2)
H57A	0.2919	0.7103	−0.0451	0.135*	0.34 (2)
H57B	0.2718	0.9042	−0.0207	0.135*	0.34 (2)
H57C	0.2171	0.7422	0.0342	0.135*	0.34 (2)
C31	0.8112 (3)	0.7289 (3)	0.70824 (12)	0.0441 (5)	
C32	0.6694 (4)	0.6501 (3)	0.75905 (17)	0.0642 (7)	
H32	0.5518	0.5977	0.7441	0.077*	
C33	0.7024 (5)	0.6491 (3)	0.83138 (17)	0.0777 (9)	
H33	0.6070	0.5956	0.8649	0.093*	
C34	0.8735 (5)	0.7259 (3)	0.85422 (15)	0.0741 (8)	
H34	0.8935	0.7264	0.9031	0.089*	
C35	1.0147 (4)	0.8017 (3)	0.80535 (14)	0.0631 (7)	
H35	1.1322	0.8530	0.8209	0.076*	
C36	0.9850 (3)	0.8032 (3)	0.73275 (13)	0.0483 (6)	
H36	1.0835	0.8550	0.6999	0.058*	
C37	0.7750 (4)	0.7325 (3)	0.62925 (15)	0.0563 (6)	
O31	0.9017 (3)	0.8122 (2)	0.58536 (9)	0.0664 (5)	
O32	0.6242 (3)	0.6520 (3)	0.61161 (12)	0.1072 (8)	
O41	0.7231 (3)	0.3783 (2)	0.45956 (10)	0.0657 (5)	
H41	0.607 (5)	0.372 (4)	0.4354 (16)	0.099*	
H42	0.839 (5)	0.318 (4)	0.4391 (16)	0.099*	

### 4-(4-Methoxyphenyl)piperazin-1-ium benzoate monohydrate (I) Atomic displacement parameters (Å^2^)

**Table:**

	*U*^11^	*U*^22^	*U*^33^	*U*^12^	*U*^13^	*U*^23^
N1	0.0614 (13)	0.0460 (11)	0.0394 (12)	−0.0089 (9)	−0.0119 (10)	−0.0069 (9)
C2	0.0452 (14)	0.0584 (14)	0.0529 (16)	−0.0018 (11)	−0.0116 (11)	−0.0075 (12)
C3	0.0424 (13)	0.0583 (14)	0.0483 (15)	0.0016 (10)	−0.0047 (10)	−0.0084 (11)
N4	0.0391 (10)	0.0403 (10)	0.0386 (11)	0.0002 (7)	−0.0018 (8)	−0.0064 (8)
C5	0.0414 (12)	0.0553 (13)	0.0413 (14)	0.0022 (10)	−0.0001 (10)	−0.0072 (10)
C6	0.0561 (14)	0.0529 (14)	0.0438 (15)	0.0044 (11)	−0.0012 (11)	−0.0083 (11)
C21	0.0428 (15)	0.022 (6)	0.0377 (14)	−0.001 (3)	−0.0009 (10)	−0.002 (2)
C22	0.0449 (14)	0.041 (5)	0.048 (2)	0.005 (3)	−0.0021 (13)	−0.007 (3)
C23	0.0575 (16)	0.053 (6)	0.048 (2)	0.008 (3)	0.0060 (13)	−0.015 (3)
C24	0.0628 (16)	0.031 (5)	0.0378 (17)	−0.004 (3)	−0.0012 (13)	−0.002 (3)
C25	0.057 (3)	0.072 (2)	0.047 (2)	0.020 (3)	−0.013 (2)	−0.0097 (16)
C26	0.057 (2)	0.054 (7)	0.0477 (16)	0.022 (2)	−0.0054 (14)	−0.014 (3)
O24	0.083 (2)	0.110 (10)	0.0410 (12)	0.009 (3)	−0.0107 (11)	−0.023 (3)
C27	0.081 (5)	0.140 (3)	0.052 (3)	−0.008 (4)	−0.018 (4)	−0.020 (4)
C51	0.0428 (15)	0.022 (6)	0.0377 (14)	−0.001 (3)	−0.0009 (10)	−0.002 (2)
C52	0.0449 (14)	0.041 (5)	0.048 (2)	0.005 (3)	−0.0021 (13)	−0.007 (3)
C53	0.0575 (16)	0.053 (6)	0.048 (2)	0.008 (3)	0.0060 (13)	−0.015 (3)
C54	0.0628 (16)	0.031 (5)	0.0378 (17)	−0.004 (3)	−0.0012 (13)	−0.002 (3)
C55	0.057 (3)	0.072 (2)	0.047 (2)	0.020 (3)	−0.013 (2)	−0.0097 (16)
C56	0.057 (2)	0.054 (7)	0.0477 (16)	0.022 (2)	−0.0054 (14)	−0.014 (3)
O54	0.083 (2)	0.110 (10)	0.0410 (12)	0.009 (3)	−0.0107 (11)	−0.023 (3)
C57	0.081 (5)	0.140 (3)	0.052 (3)	−0.008 (4)	−0.018 (4)	−0.020 (4)
C31	0.0428 (12)	0.0353 (11)	0.0550 (15)	0.0023 (10)	−0.0041 (11)	−0.0117 (10)
C32	0.0510 (15)	0.0506 (15)	0.092 (2)	−0.0061 (11)	0.0098 (14)	−0.0174 (14)
C33	0.091 (2)	0.0614 (17)	0.073 (2)	0.0018 (15)	0.0343 (17)	0.0038 (15)
C34	0.099 (2)	0.0639 (17)	0.0554 (19)	0.0101 (16)	−0.0013 (17)	−0.0025 (14)
C35	0.0746 (18)	0.0624 (16)	0.0530 (18)	−0.0031 (13)	−0.0179 (14)	−0.0044 (13)
C36	0.0509 (13)	0.0454 (13)	0.0492 (16)	−0.0057 (10)	−0.0079 (11)	−0.0023 (10)
C37	0.0540 (15)	0.0504 (15)	0.0680 (19)	0.0062 (12)	−0.0207 (13)	−0.0233 (13)
O31	0.0856 (13)	0.0687 (11)	0.0468 (11)	−0.0072 (10)	−0.0138 (9)	−0.0092 (9)
O32	0.0869 (14)	0.1422 (19)	0.1065 (18)	−0.0380 (13)	−0.0368 (12)	−0.0380 (15)
O41	0.0653 (12)	0.0631 (11)	0.0715 (14)	−0.0142 (9)	−0.0198 (9)	−0.0044 (9)

### 4-(4-Methoxyphenyl)piperazin-1-ium benzoate monohydrate (I) Geometric parameters (Å, º)

**Table:**

N1—C2	1.480 (3)	C27—H27C	0.9600
N1—C6	1.483 (3)	C51—C56	1.381 (7)
N1—H11	0.90 (2)	C51—C52	1.385 (7)
N1—H12	0.97 (2)	C52—C53	1.380 (7)
C2—C3	1.504 (3)	C52—H52	0.9300
C2—H2A	0.9700	C53—C54	1.368 (7)
C2—H2B	0.9700	C53—H53	0.9300
C3—N4	1.461 (2)	C54—C55	1.374 (9)
C3—H3A	0.9700	C54—O54	1.380 (7)
C3—H3B	0.9700	C55—C56	1.383 (9)
N4—C51	1.42 (2)	C55—H55	0.9300
N4—C21	1.428 (10)	C56—H56	0.9300
N4—C5	1.464 (2)	O54—C57	1.415 (9)
C5—C6	1.507 (3)	C57—H57A	0.9600
C5—H5A	0.9700	C57—H57B	0.9600
C5—H5B	0.9700	C57—H57C	0.9600
C6—H6A	0.9700	C31—C36	1.380 (3)
C6—H6B	0.9700	C31—C32	1.391 (3)
C21—C26	1.382 (4)	C31—C37	1.504 (3)
C21—C22	1.386 (4)	C32—C33	1.378 (4)
C22—C23	1.380 (4)	C32—H32	0.9300
C22—H22	0.9300	C33—C34	1.363 (4)
C23—C24	1.367 (4)	C33—H33	0.9300
C23—H23	0.9300	C34—C35	1.358 (3)
C24—C25	1.372 (7)	C34—H34	0.9300
C24—O24	1.378 (4)	C35—C36	1.379 (3)
C25—C26	1.382 (5)	C35—H35	0.9300
C25—H25	0.9300	C36—H36	0.9300
C26—H26	0.9300	C37—O32	1.238 (3)
O24—C27	1.413 (6)	C37—O31	1.258 (3)
C27—H27A	0.9600	O41—H41	0.88 (3)
C27—H27B	0.9600	O41—H42	0.91 (3)
			
C2—N1—C6	109.95 (18)	O24—C27—H27A	109.5
C2—N1—H11	106.8 (14)	O24—C27—H27B	109.5
C6—N1—H11	115.6 (14)	H27A—C27—H27B	109.5
C2—N1—H12	108.2 (12)	O24—C27—H27C	109.5
C6—N1—H12	109.2 (12)	H27A—C27—H27C	109.5
H11—N1—H12	106.8 (18)	H27B—C27—H27C	109.5
N1—C2—C3	110.23 (17)	C56—C51—C52	116.4 (9)
N1—C2—H2A	109.6	C56—C51—N4	125.0 (16)
C3—C2—H2A	109.6	C52—C51—N4	118.6 (16)
N1—C2—H2B	109.6	C53—C52—C51	121.0 (9)
C3—C2—H2B	109.6	C53—C52—H52	119.5
H2A—C2—H2B	108.1	C51—C52—H52	119.5
N4—C3—C2	112.64 (17)	C54—C53—C52	121.0 (9)
N4—C3—H3A	109.1	C54—C53—H53	119.5
C2—C3—H3A	109.1	C52—C53—H53	119.5
N4—C3—H3B	109.1	C53—C54—C55	118.1 (9)
C2—C3—H3B	109.1	C53—C54—O54	116.3 (10)
H3A—C3—H3B	107.8	C55—C54—O54	123.3 (12)
C51—N4—C3	120.7 (9)	C54—C55—C56	118.8 (13)
C21—N4—C3	113.1 (5)	C54—C55—H55	120.6
C51—N4—C5	114.2 (13)	C56—C55—H55	120.6
C21—N4—C5	114.4 (6)	C51—C56—C55	122.1 (10)
C3—N4—C5	111.20 (16)	C51—C56—H56	119.0
N4—C5—C6	111.63 (17)	C55—C56—H56	119.0
N4—C5—H5A	109.3	C54—O54—C57	117.7 (12)
C6—C5—H5A	109.3	O54—C57—H57A	109.5
N4—C5—H5B	109.3	O54—C57—H57B	109.5
C6—C5—H5B	109.3	H57A—C57—H57B	109.5
H5A—C5—H5B	108.0	O54—C57—H57C	109.5
N1—C6—C5	110.23 (17)	H57A—C57—H57C	109.5
N1—C6—H6A	109.6	H57B—C57—H57C	109.5
C5—C6—H6A	109.6	C36—C31—C32	117.9 (2)
N1—C6—H6B	109.6	C36—C31—C37	121.4 (2)
C5—C6—H6B	109.6	C32—C31—C37	120.8 (2)
H6A—C6—H6B	108.1	C33—C32—C31	120.4 (2)
C26—C21—C22	116.0 (4)	C33—C32—H32	119.8
C26—C21—N4	119.5 (8)	C31—C32—H32	119.8
C22—C21—N4	124.4 (8)	C34—C33—C32	120.6 (3)
C23—C22—C21	121.2 (4)	C34—C33—H33	119.7
C23—C22—H22	119.4	C32—C33—H33	119.7
C21—C22—H22	119.4	C35—C34—C33	119.8 (3)
C24—C23—C22	121.5 (4)	C35—C34—H34	120.1
C24—C23—H23	119.3	C33—C34—H34	120.1
C22—C23—H23	119.3	C34—C35—C36	120.5 (2)
C23—C24—C25	118.5 (4)	C34—C35—H35	119.7
C23—C24—O24	116.6 (5)	C36—C35—H35	119.7
C25—C24—O24	124.6 (6)	C35—C36—C31	120.9 (2)
C24—C25—C26	119.5 (7)	C35—C36—H36	119.6
C24—C25—H25	120.3	C31—C36—H36	119.6
C26—C25—H25	120.3	O32—C37—O31	124.1 (3)
C21—C26—C25	122.9 (5)	O32—C37—C31	117.3 (3)
C21—C26—H26	118.5	O31—C37—C31	118.6 (2)
C25—C26—H26	118.5	H41—O41—H42	111 (3)
C24—O24—C27	118.1 (6)		
			
C6—N1—C2—C3	−57.3 (2)	C3—N4—C51—C56	166 (4)
N1—C2—C3—N4	55.6 (2)	C5—N4—C51—C56	30 (5)
C2—C3—N4—C51	168.3 (19)	C21—N4—C51—C52	−56 (9)
C2—C3—N4—C21	175.8 (8)	C3—N4—C51—C52	−13 (4)
C2—C3—N4—C5	−53.8 (2)	C5—N4—C51—C52	−149 (2)
C51—N4—C5—C6	−164.9 (15)	C56—C51—C52—C53	−4 (5)
C21—N4—C5—C6	−176.0 (7)	N4—C51—C52—C53	175 (2)
C3—N4—C5—C6	54.3 (2)	C51—C52—C53—C54	5 (3)
C2—N1—C6—C5	58.3 (2)	C52—C53—C54—C55	−12 (4)
N4—C5—C6—N1	−57.0 (2)	C52—C53—C54—O54	−176 (3)
C51—N4—C21—C26	−49 (10)	C53—C54—C55—C56	18 (6)
C3—N4—C21—C26	170.7 (16)	O54—C54—C55—C56	−180 (5)
C5—N4—C21—C26	42 (2)	C52—C51—C56—C55	10 (7)
C51—N4—C21—C22	128 (12)	N4—C51—C56—C55	−169 (4)
C3—N4—C21—C22	−12.5 (17)	C54—C55—C56—C51	−17 (8)
C5—N4—C21—C22	−141.2 (12)	C53—C54—O54—C57	161 (4)
C26—C21—C22—C23	2 (2)	C55—C54—O54—C57	−2 (7)
N4—C21—C22—C23	−174.5 (11)	C36—C31—C32—C33	0.7 (3)
C21—C22—C23—C24	−2.6 (14)	C37—C31—C32—C33	−179.3 (2)
C22—C23—C24—C25	4.6 (17)	C31—C32—C33—C34	0.3 (4)
C22—C23—C24—O24	178.6 (15)	C32—C33—C34—C35	−1.0 (4)
C23—C24—C25—C26	−6 (3)	C33—C34—C35—C36	0.7 (4)
O24—C24—C25—C26	−180 (2)	C34—C35—C36—C31	0.4 (3)
C22—C21—C26—C25	−4 (3)	C32—C31—C36—C35	−1.0 (3)
N4—C21—C26—C25	173 (2)	C37—C31—C36—C35	178.9 (2)
C24—C25—C26—C21	7 (4)	C36—C31—C37—O32	175.1 (2)
C23—C24—O24—C27	171 (2)	C32—C31—C37—O32	−5.0 (3)
C25—C24—O24—C27	−16 (3)	C36—C31—C37—O31	−3.1 (3)
C21—N4—C51—C56	123 (14)	C32—C31—C37—O31	176.9 (2)

### 4-(4-Methoxyphenyl)piperazin-1-ium benzoate monohydrate (I) Hydrogen-bond geometry (Å, º)

**Table:**

*D*—H···*A*	*D*—H	H···*A*	*D*···*A*	*D*—H···*A*
N1—H11···O31	0.90 (2)	1.88 (2)	2.777 (3)	174.1 (19)
N1—H12···O41	0.97 (2)	1.85 (2)	2.808 (3)	169.7 (18)
O41—H41···O32^i^	0.88 (3)	1.75 (3)	2.631 (3)	177 (3)
O41—H42···O31^ii^	0.91 (3)	1.87 (3)	2.763 (3)	169 (3)
C2—H2*B*···O31^iii^	0.97	2.54	3.485 (3)	165
C22—H22···*Cg*1^ii^	0.93	2.85	3.603 (3)	139
C26—H26···*Cg*1^iv^	0.93	2.90	3.62 (2)	135
C56—H56···*Cg*1^iv^	0.93	2.64	3.41 (5)	141

Symmetry codes: (i) −*x*+1, −*y*+1, −*z*+1; (ii) −*x*+2, −*y*+1, −*z*+1; (iii) −*x*+2, −*y*+2, −*z*+1; (iv) −*x*+1, −*y*+2, −*z*+1.

### 4-(4-Methoxyphenyl)piperazin-1-ium 4-fluorobenzoate monohydrate (II) Crystal data

**Table:**

C_11_H_17_N_2_O^+^·C_7_H_4_FO_2_^−^·H_2_O	*Z* = 2
*M**_r_* = 350.38	*F*(000) = 372
Triclinic, *P*1	*D*_x_ = 1.314 Mg m^−^^3^
*a* = 6.256 (1) Å	Mo *K*α radiation, λ = 0.71073 Å
*b* = 7.489 (1) Å	Cell parameters from 3771 reflections
*c* = 19.097 (2) Å	θ = 2.9–27.9°
α = 84.19 (1)°	µ = 0.10 mm^−^^1^
β = 86.98 (2)°	*T* = 293 K
γ = 84.62 (2)°	Plate, colourless
*V* = 885.4 (2) Å^3^	0.40 × 0.24 × 0.04 mm

### 4-(4-Methoxyphenyl)piperazin-1-ium 4-fluorobenzoate monohydrate (II) Data collection

**Table:**

Oxford Diffraction Xcalibur with Sapphire CCD diffractometer	3477 independent reflections
Radiation source: Enhance (Mo) X-ray Source	1355 reflections with *I* > 2σ(*I*)
Graphite monochromator	*R*_int_ = 0.046
ω scans	θ_max_ = 26.1°, θ_min_ = 2.9°
Absorption correction: multi-scan (CrysAlis RED; Oxford Diffraction, 2009)	*h* = −7→6
*T*_min_ = 0.973, *T*_max_ = 0.996	*k* = −9→9
5760 measured reflections	*l* = −23→22

### 4-(4-Methoxyphenyl)piperazin-1-ium 4-fluorobenzoate monohydrate (II) Refinement

**Table:**

Refinement on *F*^2^	Primary atom site location: difference Fourier map
Least-squares matrix: full	Hydrogen site location: mixed
*R*[*F*^2^ > 2σ(*F*^2^)] = 0.066	H atoms treated by a mixture of independent and constrained refinement
*wR*(*F*^2^) = 0.128	*w* = 1/[σ^2^(*F*_o_^2^) + (0.0404*P*)^2^] where *P* = (*F*_o_^2^ + 2*F*_c_^2^)/3
*S* = 1.01	(Δ/σ)_max_ < 0.001
3477 reflections	Δρ_max_ = 0.13 e Å^−^^3^
265 parameters	Δρ_min_ = −0.14 e Å^−^^3^
17 restraints	

### 4-(4-Methoxyphenyl)piperazin-1-ium 4-fluorobenzoate monohydrate (II) Special details

**Table:**

Experimental. Compound (II). IR (KBr , cm^-1^) 3317 (OH), 3011 (NH_2_), 2838 (OCH_3_), 1588 (COO), 1365 (CF) NMR (CDCl_3_) δ(^1^H) 3.23 (m, 4H, piperazine), 3.29 (m, 4H, piperazine), 3.77 (s, 3H, OCH_3_), 6.86 (m, 4H, methoxyphenyl), 7.05 (m, 2H, fluorophenyl), 8.05 (m, 2H, fluorophenyl).
Geometry. All esds (except the esd in the dihedral angle between two l.s. planes) are estimated using the full covariance matrix. The cell esds are taken into account individually in the estimation of esds in distances, angles and torsion angles; correlations between esds in cell parameters are only used when they are defined by crystal symmetry. An approximate (isotropic) treatment of cell esds is used for estimating esds involving l.s. planes.

### 4-(4-Methoxyphenyl)piperazin-1-ium 4-fluorobenzoate monohydrate (II) Fractional atomic coordinates and isotropic or equivalent isotropic displacement parameters (Å^2^)

**Table:**

	*x*	*y*	*z*	*U*_iso_*/*U*_eq_	Occ. (<1)
N1	0.7813 (5)	0.7442 (3)	0.45140 (15)	0.0536 (8)	
H11	0.823 (4)	0.778 (3)	0.5031 (15)	0.064*	
H12	0.749 (4)	0.634 (4)	0.4565 (14)	0.064*	
C2	0.9661 (5)	0.7540 (4)	0.39966 (16)	0.0599 (9)	
H2A	1.0848	0.6706	0.4164	0.072*	
H2B	1.0131	0.8746	0.3952	0.072*	
C3	0.9056 (5)	0.7083 (4)	0.32889 (15)	0.0574 (9)	
H3A	1.0264	0.7229	0.2955	0.069*	
H3B	0.8755	0.5829	0.3326	0.069*	
N4	0.7185 (4)	0.8206 (3)	0.30227 (12)	0.0444 (6)	
C5	0.5377 (5)	0.8151 (4)	0.35391 (14)	0.0527 (8)	
H5A	0.4900	0.6947	0.3596	0.063*	
H5B	0.4192	0.8977	0.3365	0.063*	
C6	0.5960 (5)	0.8649 (4)	0.42410 (14)	0.0567 (9)	
H6A	0.6324	0.9887	0.4194	0.068*	
H6B	0.4736	0.8556	0.4572	0.068*	
C21	0.670 (3)	0.795 (6)	0.2319 (6)	0.0442 (18)	0.81 (3)
C22	0.8062 (12)	0.6932 (18)	0.1883 (4)	0.064 (2)	0.81 (3)
H22	0.9351	0.6378	0.2053	0.077*	0.81 (3)
C23	0.7544 (9)	0.673 (2)	0.1207 (3)	0.069 (3)	0.81 (3)
H23	0.8520	0.6083	0.0923	0.083*	0.81 (3)
C24	0.5644 (10)	0.7455 (18)	0.0942 (3)	0.058 (2)	0.81 (3)
C25	0.4255 (15)	0.846 (3)	0.1357 (5)	0.0680 (18)	0.81 (3)
H25	0.2967	0.9002	0.1182	0.082*	0.81 (3)
C26	0.4782 (18)	0.866 (3)	0.2037 (5)	0.062 (2)	0.81 (3)
H26	0.3798	0.9313	0.2317	0.074*	0.81 (3)
O24	0.5299 (13)	0.7159 (19)	0.0261 (3)	0.095 (2)	0.81 (3)
C27	0.3282 (18)	0.772 (3)	−0.0015 (6)	0.107 (4)	0.81 (3)
H27A	0.2181	0.7178	0.0279	0.160*	0.81 (3)
H27B	0.3249	0.7366	−0.0483	0.160*	0.81 (3)
H27C	0.3035	0.9011	−0.0029	0.160*	0.81 (3)
C51	0.655 (11)	0.79 (3)	0.236 (2)	0.0442 (18)	0.19 (3)
C52	0.810 (6)	0.754 (6)	0.1833 (17)	0.064 (2)	0.19 (3)
H52	0.9531	0.7290	0.1949	0.077*	0.19 (3)
C53	0.756 (4)	0.752 (6)	0.1146 (15)	0.069 (3)	0.19 (3)
H53	0.8593	0.7106	0.0818	0.083*	0.19 (3)
C54	0.554 (4)	0.808 (5)	0.0936 (13)	0.058 (2)	0.19 (3)
C55	0.396 (6)	0.842 (13)	0.144 (2)	0.0680 (18)	0.19 (3)
H55	0.2524	0.8581	0.1327	0.082*	0.19 (3)
C56	0.453 (7)	0.851 (13)	0.213 (2)	0.062 (2)	0.19 (3)
H56	0.3509	0.8987	0.2444	0.074*	0.19 (3)
O54	0.512 (5)	0.786 (6)	0.0251 (14)	0.095 (2)	0.19 (3)
C57	0.300 (7)	0.827 (12)	0.004 (3)	0.107 (4)	0.19 (3)
H57A	0.2472	0.9444	0.0166	0.160*	0.19 (3)
H57B	0.2101	0.7391	0.0266	0.160*	0.19 (3)
H57C	0.2965	0.8256	−0.0463	0.160*	0.19 (3)
C31	0.8132 (5)	0.7284 (4)	0.70495 (17)	0.0485 (8)	
C32	0.6742 (5)	0.6533 (4)	0.7564 (2)	0.0676 (10)	
H32	0.5542	0.6028	0.7431	0.081*	
C33	0.7114 (6)	0.6527 (5)	0.8267 (2)	0.0798 (11)	
H33	0.6181	0.6022	0.8611	0.096*	
C34	0.8883 (7)	0.7278 (5)	0.8448 (2)	0.0752 (11)	
F34	0.9236 (3)	0.7291 (3)	0.91437 (11)	0.1208 (9)	
C35	1.0294 (5)	0.8010 (4)	0.7961 (2)	0.0658 (10)	
H35	1.1495	0.8505	0.8100	0.079*	
C36	0.9911 (5)	0.8003 (4)	0.72606 (17)	0.0542 (9)	
H36	1.0870	0.8494	0.6922	0.065*	
C37	0.7690 (6)	0.7297 (5)	0.6289 (2)	0.0614 (10)	
O31	0.8943 (4)	0.8069 (3)	0.58380 (12)	0.0715 (7)	
O32	0.6112 (4)	0.6566 (4)	0.61332 (13)	0.1091 (10)	
O41	0.7239 (4)	0.3737 (3)	0.46049 (12)	0.0700 (8)	
H41	0.615 (6)	0.356 (5)	0.4356 (17)	0.105*	
H42	0.835 (6)	0.312 (5)	0.4379 (18)	0.105*	

### 4-(4-Methoxyphenyl)piperazin-1-ium 4-fluorobenzoate monohydrate (II) Atomic displacement parameters (Å^2^)

**Table:**

	*U*^11^	*U*^22^	*U*^33^	*U*^12^	*U*^13^	*U*^23^
N1	0.065 (2)	0.0429 (18)	0.0550 (18)	−0.0089 (16)	−0.0145 (16)	−0.0061 (15)
C2	0.050 (2)	0.060 (3)	0.070 (2)	−0.0047 (18)	−0.0107 (19)	−0.0069 (18)
C3	0.049 (2)	0.065 (2)	0.059 (2)	−0.0019 (18)	−0.0035 (17)	−0.0091 (18)
N4	0.0405 (16)	0.0437 (17)	0.0483 (16)	0.0016 (13)	−0.0044 (13)	−0.0054 (12)
C5	0.049 (2)	0.057 (2)	0.052 (2)	−0.0005 (16)	−0.0015 (17)	−0.0083 (16)
C6	0.059 (2)	0.057 (2)	0.053 (2)	0.0029 (18)	−0.0011 (17)	−0.0049 (17)
C21	0.044 (3)	0.042 (3)	0.047 (2)	−0.004 (4)	0.001 (2)	−0.008 (3)
C22	0.055 (2)	0.070 (7)	0.068 (3)	0.009 (3)	−0.005 (2)	−0.020 (3)
C23	0.063 (3)	0.080 (8)	0.067 (3)	0.009 (3)	0.004 (2)	−0.030 (4)
C24	0.066 (3)	0.058 (6)	0.053 (3)	−0.007 (3)	−0.003 (2)	−0.017 (3)
C25	0.061 (3)	0.087 (3)	0.054 (4)	0.012 (4)	−0.009 (3)	−0.009 (4)
C26	0.056 (3)	0.070 (5)	0.056 (3)	0.016 (4)	−0.001 (2)	−0.013 (4)
O24	0.094 (3)	0.129 (7)	0.0660 (19)	0.008 (4)	−0.0132 (16)	−0.039 (3)
C27	0.102 (5)	0.156 (14)	0.067 (4)	−0.004 (5)	−0.027 (4)	−0.023 (4)
C51	0.044 (3)	0.042 (3)	0.047 (2)	−0.004 (4)	0.001 (2)	−0.008 (3)
C52	0.055 (2)	0.070 (7)	0.068 (3)	0.009 (3)	−0.005 (2)	−0.020 (3)
C53	0.063 (3)	0.080 (8)	0.067 (3)	0.009 (3)	0.004 (2)	−0.030 (4)
C54	0.066 (3)	0.058 (6)	0.053 (3)	−0.007 (3)	−0.003 (2)	−0.017 (3)
C55	0.061 (3)	0.087 (3)	0.054 (4)	0.012 (4)	−0.009 (3)	−0.009 (4)
C56	0.056 (3)	0.070 (5)	0.056 (3)	0.016 (4)	−0.001 (2)	−0.013 (4)
O54	0.094 (3)	0.129 (7)	0.0660 (19)	0.008 (4)	−0.0132 (16)	−0.039 (3)
C57	0.102 (5)	0.156 (14)	0.067 (4)	−0.004 (5)	−0.027 (4)	−0.023 (4)
C31	0.045 (2)	0.039 (2)	0.063 (2)	0.0008 (16)	−0.0084 (18)	−0.0119 (16)
C32	0.051 (2)	0.055 (3)	0.099 (3)	−0.0062 (18)	−0.002 (2)	−0.017 (2)
C33	0.072 (3)	0.073 (3)	0.089 (3)	−0.004 (2)	0.020 (2)	−0.001 (2)
C34	0.083 (3)	0.084 (3)	0.057 (3)	0.003 (2)	−0.009 (2)	−0.004 (2)
F34	0.131 (2)	0.164 (2)	0.0633 (15)	0.0008 (17)	−0.0068 (13)	−0.0036 (14)
C35	0.059 (2)	0.068 (3)	0.072 (3)	−0.0106 (19)	−0.011 (2)	−0.006 (2)
C36	0.056 (2)	0.052 (2)	0.056 (2)	−0.0059 (18)	−0.0122 (17)	−0.0070 (16)
C37	0.057 (3)	0.042 (2)	0.087 (3)	0.0070 (19)	−0.022 (2)	−0.019 (2)
O31	0.0848 (18)	0.0686 (18)	0.0638 (17)	−0.0057 (15)	−0.0208 (14)	−0.0123 (13)
O32	0.0871 (19)	0.129 (3)	0.124 (2)	−0.0362 (18)	−0.0427 (16)	−0.0343 (17)
O41	0.0693 (17)	0.0628 (18)	0.0816 (18)	−0.0137 (14)	−0.0227 (13)	−0.0073 (13)

### 4-(4-Methoxyphenyl)piperazin-1-ium 4-fluorobenzoate monohydrate (II) Geometric parameters (Å, º)

**Table:**

N1—C2	1.483 (4)	C27—H27C	0.9600
N1—C6	1.485 (4)	C51—C56	1.380 (9)
N1—H11	1.09 (3)	C51—C52	1.390 (19)
N1—H12	0.87 (3)	C52—C53	1.374 (9)
C2—C3	1.503 (3)	C52—H52	0.9300
C2—H2A	0.9700	C53—C54	1.362 (9)
C2—H2B	0.9700	C53—H53	0.9300
C3—N4	1.458 (3)	C54—C55	1.370 (13)
C3—H3A	0.9700	C54—O54	1.377 (9)
C3—H3B	0.9700	C55—C56	1.383 (9)
N4—C51	1.39 (5)	C55—H55	0.9300
N4—C21	1.429 (10)	C56—H56	0.9300
N4—C5	1.462 (3)	O54—C57	1.408 (10)
C5—C6	1.499 (3)	C57—H57A	0.9600
C5—H5A	0.9700	C57—H57B	0.9600
C5—H5B	0.9700	C57—H57C	0.9600
C6—H6A	0.9700	C31—C36	1.377 (3)
C6—H6B	0.9700	C31—C32	1.388 (4)
C21—C26	1.379 (6)	C31—C37	1.491 (4)
C21—C22	1.389 (15)	C32—C33	1.374 (4)
C22—C23	1.374 (4)	C32—H32	0.9300
C22—H22	0.9300	C33—C34	1.363 (4)
C23—C24	1.361 (5)	C33—H33	0.9300
C23—H23	0.9300	C34—C35	1.358 (4)
C24—C25	1.369 (9)	C34—F34	1.360 (4)
C24—O24	1.373 (4)	C35—C36	1.373 (4)
C25—C26	1.383 (5)	C35—H35	0.9300
C25—H25	0.9300	C36—H36	0.9300
C26—H26	0.9300	C37—O32	1.236 (3)
O24—C27	1.405 (4)	C37—O31	1.266 (4)
C27—H27A	0.9600	O41—H41	0.88 (3)
C27—H27B	0.9600	O41—H42	0.91 (4)
			
C2—N1—C6	109.5 (2)	O24—C27—H27A	109.5
C2—N1—H11	111.6 (13)	O24—C27—H27B	109.5
C6—N1—H11	111.1 (13)	H27A—C27—H27B	109.5
C2—N1—H12	107 (2)	O24—C27—H27C	109.5
C6—N1—H12	110.0 (19)	H27A—C27—H27C	109.5
H11—N1—H12	107 (2)	H27B—C27—H27C	109.5
N1—C2—C3	110.9 (2)	C56—C51—C52	115.3 (18)
N1—C2—H2A	109.5	C56—C51—N4	122 (4)
C3—C2—H2A	109.5	C52—C51—N4	120 (5)
N1—C2—H2B	109.5	C53—C52—C51	121.2 (14)
C3—C2—H2B	109.5	C53—C52—H52	119.4
H2A—C2—H2B	108.1	C51—C52—H52	119.4
N4—C3—C2	112.7 (2)	C54—C53—C52	121.5 (11)
N4—C3—H3A	109.0	C54—C53—H53	119.3
C2—C3—H3A	109.0	C52—C53—H53	119.3
N4—C3—H3B	109.0	C53—C54—C55	118.3 (12)
C2—C3—H3B	109.0	C53—C54—O54	116.6 (13)
H3A—C3—H3B	107.8	C55—C54—O54	123.2 (15)
C51—N4—C3	117 (4)	C54—C55—C56	119.0 (16)
C21—N4—C3	114.4 (9)	C54—C55—H55	120.5
C51—N4—C5	111 (4)	C56—C55—H55	120.5
C21—N4—C5	115.5 (10)	C51—C56—C55	122.8 (13)
C3—N4—C5	110.9 (2)	C51—C56—H56	118.6
N4—C5—C6	112.1 (2)	C55—C56—H56	118.6
N4—C5—H5A	109.2	C54—O54—C57	117.8 (15)
C6—C5—H5A	109.2	O54—C57—H57A	109.5
N4—C5—H5B	109.2	O54—C57—H57B	109.5
C6—C5—H5B	109.2	H57A—C57—H57B	109.5
H5A—C5—H5B	107.9	O54—C57—H57C	109.5
N1—C6—C5	110.6 (2)	H57A—C57—H57C	109.5
N1—C6—H6A	109.5	H57B—C57—H57C	109.5
C5—C6—H6A	109.5	C36—C31—C32	118.3 (3)
N1—C6—H6B	109.5	C36—C31—C37	121.4 (3)
C5—C6—H6B	109.5	C32—C31—C37	120.2 (3)
H6A—C6—H6B	108.1	C33—C32—C31	121.0 (3)
C26—C21—C22	115.6 (7)	C33—C32—H32	119.5
C26—C21—N4	121.3 (11)	C31—C32—H32	119.5
C22—C21—N4	123.1 (9)	C34—C33—C32	118.3 (4)
C23—C22—C21	121.3 (4)	C34—C33—H33	120.8
C23—C22—H22	119.4	C32—C33—H33	120.8
C21—C22—H22	119.4	C35—C34—F34	119.1 (4)
C24—C23—C22	121.8 (4)	C35—C34—C33	122.6 (4)
C24—C23—H23	119.1	F34—C34—C33	118.3 (4)
C22—C23—H23	119.1	C34—C35—C36	118.6 (3)
C23—C24—C25	118.5 (4)	C34—C35—H35	120.7
C23—C24—O24	116.6 (4)	C36—C35—H35	120.7
C25—C24—O24	124.8 (4)	C35—C36—C31	121.2 (3)
C24—C25—C26	119.5 (6)	C35—C36—H36	119.4
C24—C25—H25	120.2	C31—C36—H36	119.4
C26—C25—H25	120.2	O32—C37—O31	123.5 (4)
C21—C26—C25	123.2 (6)	O32—C37—C31	118.3 (4)
C21—C26—H26	118.4	O31—C37—C31	118.2 (3)
C25—C26—H26	118.4	H41—O41—H42	101 (3)
C24—O24—C27	118.8 (4)		
			
C6—N1—C2—C3	−56.3 (3)	C3—N4—C51—C52	−38 (19)
N1—C2—C3—N4	55.3 (3)	C5—N4—C51—C52	−166 (12)
C2—C3—N4—C51	178 (9)	C56—C51—C52—C53	−9 (19)
C2—C3—N4—C21	174 (2)	N4—C51—C52—C53	−170 (10)
C2—C3—N4—C5	−53.7 (3)	C51—C52—C53—C54	8 (11)
C51—N4—C5—C6	−174 (8)	C52—C53—C54—C55	−10 (7)
C21—N4—C5—C6	−173 (2)	C52—C53—C54—O54	−175 (4)
C3—N4—C5—C6	54.6 (3)	C53—C54—C55—C56	12 (11)
C2—N1—C6—C5	57.4 (3)	O54—C54—C55—C56	176 (7)
N4—C5—C6—N1	−57.2 (3)	N4—C51—C56—C55	172 (13)
C3—N4—C21—C26	169 (3)	C54—C55—C56—C51	−14 (15)
C5—N4—C21—C26	38 (4)	C53—C54—O54—C57	174 (5)
C3—N4—C21—C22	−10 (5)	C55—C54—O54—C57	10 (8)
C5—N4—C21—C22	−140 (3)	C36—C31—C32—C33	0.8 (4)
C26—C21—C22—C23	2 (4)	C37—C31—C32—C33	−179.3 (3)
N4—C21—C22—C23	−179 (2)	C31—C32—C33—C34	0.1 (5)
C21—C22—C23—C24	−3 (2)	C32—C33—C34—C35	−0.7 (5)
C22—C23—C24—C25	2.3 (13)	C32—C33—C34—F34	179.2 (3)
C22—C23—C24—O24	−179.7 (6)	F34—C34—C35—C36	−179.4 (3)
C23—C24—C25—C26	−2 (2)	C33—C34—C35—C36	0.5 (5)
O24—C24—C25—C26	−179.9 (14)	C34—C35—C36—C31	0.4 (5)
C22—C21—C26—C25	−2 (4)	C32—C31—C36—C35	−1.0 (4)
N4—C21—C26—C25	179 (3)	C37—C31—C36—C35	179.1 (3)
C24—C25—C26—C21	2 (3)	C36—C31—C37—O32	177.2 (3)
C23—C24—O24—C27	173.8 (10)	C32—C31—C37—O32	−2.7 (4)
C25—C24—O24—C27	−8.4 (16)	C36—C31—C37—O31	−3.3 (4)
C3—N4—C51—C56	162 (12)	C32—C31—C37—O31	176.8 (3)
C5—N4—C51—C56	34 (18)		

### 4-(4-Methoxyphenyl)piperazin-1-ium 4-fluorobenzoate monohydrate (II) Hydrogen-bond geometry (Å, º)

**Table:**

*D*—H···*A*	*D*—H	H···*A*	*D*···*A*	*D*—H···*A*
N1—H11···O31	1.09 (3)	1.67 (3)	2.758 (4)	174.1 (19)
N1—H12···O41	0.86 (3)	1.96 (3)	2.818 (4)	170 (3)
O41—H41···O32^i^	0.86 (4)	1.75 (4)	2.627 (4)	174 (4)
O41—H42···O31^ii^	0.91 (4)	1.88 (4)	2.768 (3)	163 (3)
C2—H2*B*···O31^iii^	0.97	2.58	3.529 (4)	166
C6—H6*B*···O41^i^	0.97	2.57	3.386 (4)	142
C22—H22···*Cg*1^ii^	0.93	2.93	3.664 (12)	137
C26—H26···*Cg*1^iv^	0.93	2.81	3.56 (2)	138
C56—H56···*Cg*1^iv^	0.93	2.96	3.55 (9)	123

Symmetry codes: (i) −*x*+1, −*y*+1, −*z*+1; (ii) −*x*+2, −*y*+1, −*z*+1; (iii) −*x*+2, −*y*+2, −*z*+1; (iv) −*x*+1, −*y*+2, −*z*+1.

### 4-(4-Methoxyphenyl)piperazin-1-ium 4-chlorobenzoate monohydrate (III) Crystal data

**Table:**

C_11_H_17_N_2_O^+^·C_7_H_4_ClO_2_^−^·H_2_O	*Z* = 2
*M**_r_* = 366.83	*F*(000) = 388
Triclinic, *P*1	*D*_x_ = 1.312 Mg m^−^^3^
*a* = 6.211 (1) Å	Mo *K*α radiation, λ = 0.71073 Å
*b* = 7.481 (1) Å	Cell parameters from 3962 reflections
*c* = 20.144 (4) Å	θ = 2.8–28.8°
α = 84.90 (2)°	µ = 0.23 mm^−^^1^
β = 87.48 (2)°	*T* = 293 K
γ = 85.19 (2)°	Plate, colourless
*V* = 928.4 (3) Å^3^	0.20 × 0.16 × 0.02 mm

### 4-(4-Methoxyphenyl)piperazin-1-ium 4-chlorobenzoate monohydrate (III) Data collection

**Table:**

Oxford Diffraction Xcalibur with Sapphire CCD diffractometer	3454 independent reflections
Radiation source: Enhance (Mo) X-ray Source	1343 reflections with *I* > 2σ(*I*)
Graphite monochromator	*R*_int_ = 0.041
ω scans	θ_max_ = 25.5°, θ_min_ = 2.8°
Absorption correction: multi-scan (CrysAlis RED; Oxford Diffraction, 2009)	*h* = −7→7
*T*_min_ = 0.951, *T*_max_ = 0.995	*k* = −9→8
5883 measured reflections	*l* = −24→22

### 4-(4-Methoxyphenyl)piperazin-1-ium 4-chlorobenzoate monohydrate (III) Refinement

**Table:**

Refinement on *F*^2^	Primary atom site location: difference Fourier map
Least-squares matrix: full	Hydrogen site location: mixed
*R*[*F*^2^ > 2σ(*F*^2^)] = 0.065	H atoms treated by a mixture of independent and constrained refinement
*wR*(*F*^2^) = 0.135	*w* = 1/[σ^2^(*F*_o_^2^) + (0.0492*P*)^2^] where *P* = (*F*_o_^2^ + 2*F*_c_^2^)/3
*S* = 0.94	(Δ/σ)_max_ < 0.001
3454 reflections	Δρ_max_ = 0.24 e Å^−^^3^
265 parameters	Δρ_min_ = −0.23 e Å^−^^3^
17 restraints	

### 4-(4-Methoxyphenyl)piperazin-1-ium 4-chlorobenzoate monohydrate (III) Special details

**Table:**

Experimental. Compound (III). IR (KBr , cm^-1^) 3320 (OH), 3003 (NH_2_), 2837 (OCH_3_), 1582 (COO), 772(CCl). NMR (CDCl_3_) δ(^1^H) 3.23 (m, 4H, piperazine), 3.28 (m, 4H, piperazine), 3.77 (s, 3H, OCH_3_), 6.86 (m, 4H, methoxyphenyl), 7.36 (d, J = 8.4 Hz, 2H, chlorophenyl), 7.98 (d, J = 8.4 Hz,2H, chlorophenyl).
Geometry. All esds (except the esd in the dihedral angle between two l.s. planes) are estimated using the full covariance matrix. The cell esds are taken into account individually in the estimation of esds in distances, angles and torsion angles; correlations between esds in cell parameters are only used when they are defined by crystal symmetry. An approximate (isotropic) treatment of cell esds is used for estimating esds involving l.s. planes.

### 4-(4-Methoxyphenyl)piperazin-1-ium 4-chlorobenzoate monohydrate (III) Fractional atomic coordinates and isotropic or equivalent isotropic displacement parameters (Å^2^)

**Table:**

	*x*	*y*	*z*	*U*_iso_*/*U*_eq_	Occ. (<1)
N1	0.7833 (5)	0.7442 (4)	0.45388 (15)	0.0536 (9)	
H11	0.827 (5)	0.777 (4)	0.5028 (16)	0.064*	
H12	0.760 (5)	0.636 (4)	0.4593 (16)	0.064*	
C2	0.9718 (6)	0.7498 (5)	0.40636 (16)	0.0570 (10)	
H2A	1.0888	0.6667	0.4234	0.068*	
H2B	1.0216	0.8699	0.4020	0.068*	
C3	0.9136 (6)	0.6998 (4)	0.33895 (16)	0.0545 (10)	
H3A	1.0371	0.7116	0.3082	0.065*	
H3B	0.8803	0.5749	0.3427	0.065*	
N4	0.7287 (4)	0.8126 (3)	0.31215 (12)	0.0433 (7)	
C5	0.5425 (5)	0.8110 (4)	0.35968 (15)	0.0516 (9)	
H5A	0.4918	0.6912	0.3650	0.062*	
H5B	0.4260	0.8935	0.3420	0.062*	
C6	0.5992 (6)	0.8644 (4)	0.42667 (15)	0.0551 (10)	
H6A	0.6377	0.9880	0.4222	0.066*	
H6B	0.4747	0.8569	0.4573	0.066*	
C21	0.685 (4)	0.783 (9)	0.2449 (9)	0.0446 (14)	0.73 (2)
C22	0.8261 (13)	0.6777 (19)	0.2052 (4)	0.060 (3)	0.73 (2)
H22	0.9523	0.6211	0.2230	0.072*	0.73 (2)
C23	0.7808 (13)	0.657 (2)	0.1401 (4)	0.074 (3)	0.73 (2)
H23	0.8807	0.5916	0.1141	0.088*	0.73 (2)
C24	0.5923 (18)	0.730 (3)	0.1130 (5)	0.065 (4)	0.73 (2)
C25	0.450 (2)	0.828 (5)	0.1516 (8)	0.075 (3)	0.73 (2)
H25	0.3196	0.8781	0.1341	0.089*	0.73 (2)
C26	0.496 (2)	0.854 (3)	0.2162 (6)	0.060 (3)	0.73 (2)
H26	0.3958	0.9221	0.2413	0.072*	0.73 (2)
O24	0.564 (2)	0.697 (2)	0.0482 (6)	0.104 (4)	0.73 (2)
C27	0.362 (3)	0.743 (2)	0.0203 (7)	0.101 (4)	0.73 (2)
H27A	0.2517	0.6892	0.0485	0.152*	0.73 (2)
H27B	0.3623	0.6989	−0.0231	0.152*	0.73 (2)
H27C	0.3331	0.8713	0.0164	0.152*	0.73 (2)
C51	0.668 (10)	0.79 (3)	0.247 (2)	0.0446 (14)	0.27 (2)
C52	0.833 (4)	0.755 (4)	0.1991 (13)	0.060 (3)	0.27 (2)
H52	0.9761	0.7413	0.2112	0.072*	0.27 (2)
C53	0.783 (4)	0.739 (4)	0.1340 (12)	0.074 (3)	0.27 (2)
H53	0.8924	0.7021	0.1039	0.088*	0.27 (2)
C54	0.577 (5)	0.775 (9)	0.1126 (15)	0.065 (4)	0.27 (2)
C55	0.418 (6)	0.825 (15)	0.158 (2)	0.075 (3)	0.27 (2)
H55	0.2775	0.8534	0.1437	0.089*	0.27 (2)
C56	0.464 (5)	0.834 (8)	0.2235 (18)	0.060 (3)	0.27 (2)
H56	0.3532	0.8701	0.2531	0.072*	0.27 (2)
O54	0.548 (7)	0.751 (7)	0.0468 (16)	0.104 (4)	0.27 (2)
C57	0.351 (8)	0.814 (6)	0.018 (2)	0.101 (4)	0.27 (2)
H57A	0.2346	0.7622	0.0438	0.152*	0.27 (2)
H57B	0.3518	0.7814	−0.0267	0.152*	0.27 (2)
H57C	0.3326	0.9432	0.0182	0.152*	0.27 (2)
C31	0.8074 (6)	0.7372 (4)	0.69495 (18)	0.0470 (9)	
C32	0.6605 (7)	0.6665 (5)	0.7412 (2)	0.0704 (11)	
H32	0.5407	0.6164	0.7267	0.084*	
C33	0.6886 (8)	0.6688 (5)	0.8091 (2)	0.0834 (13)	
H33	0.5883	0.6212	0.8400	0.100*	
C34	0.8662 (9)	0.7424 (6)	0.8301 (2)	0.0770 (13)	
Cl34	0.8970 (3)	0.74735 (19)	0.91487 (5)	0.1383 (7)	
C35	1.0144 (7)	0.8114 (5)	0.7851 (2)	0.0695 (11)	
H35	1.1350	0.8601	0.7997	0.083*	
C36	0.9845 (6)	0.8084 (4)	0.71778 (17)	0.0543 (10)	
H36	1.0861	0.8555	0.6872	0.065*	
C37	0.7721 (7)	0.7351 (5)	0.6222 (2)	0.0581 (11)	
O31	0.9006 (5)	0.8125 (3)	0.58079 (12)	0.0697 (8)	
O32	0.6183 (5)	0.6568 (4)	0.60573 (15)	0.1085 (11)	
O41	0.7230 (5)	0.3744 (3)	0.46334 (13)	0.0683 (8)	
H41	0.614 (7)	0.363 (5)	0.4360 (19)	0.102*	
H42	0.846 (7)	0.315 (5)	0.4434 (19)	0.102*	

### 4-(4-Methoxyphenyl)piperazin-1-ium 4-chlorobenzoate monohydrate (III) Atomic displacement parameters (Å^2^)

**Table:**

	*U*^11^	*U*^22^	*U*^33^	*U*^12^	*U*^13^	*U*^23^
N1	0.065 (2)	0.0467 (18)	0.0505 (19)	−0.0043 (19)	−0.0122 (18)	−0.0079 (17)
C2	0.044 (2)	0.069 (3)	0.057 (2)	−0.003 (2)	−0.008 (2)	−0.0023 (19)
C3	0.043 (2)	0.065 (2)	0.053 (2)	0.007 (2)	−0.0072 (19)	−0.0050 (18)
N4	0.0390 (19)	0.0473 (17)	0.0432 (17)	0.0009 (15)	−0.0023 (15)	−0.0044 (13)
C5	0.049 (2)	0.055 (2)	0.049 (2)	0.0054 (19)	0.0007 (19)	−0.0041 (18)
C6	0.057 (3)	0.056 (2)	0.051 (2)	0.006 (2)	−0.0009 (19)	−0.0052 (18)
C21	0.038 (4)	0.046 (7)	0.049 (3)	0.001 (5)	0.001 (3)	−0.005 (2)
C22	0.054 (3)	0.064 (8)	0.059 (3)	0.021 (4)	−0.007 (2)	−0.011 (4)
C23	0.073 (4)	0.084 (9)	0.059 (3)	0.035 (5)	−0.002 (3)	−0.020 (5)
C24	0.074 (4)	0.077 (12)	0.043 (2)	0.008 (3)	−0.004 (2)	−0.016 (4)
C25	0.064 (5)	0.104 (4)	0.053 (4)	0.021 (7)	−0.013 (4)	−0.013 (5)
C26	0.048 (4)	0.081 (6)	0.049 (4)	0.012 (5)	0.003 (3)	−0.012 (4)
O24	0.104 (3)	0.148 (12)	0.059 (2)	0.039 (5)	−0.0194 (19)	−0.038 (4)
C27	0.109 (5)	0.125 (13)	0.070 (3)	0.022 (7)	−0.034 (3)	−0.025 (7)
C51	0.038 (4)	0.046 (7)	0.049 (3)	0.001 (5)	0.001 (3)	−0.005 (2)
C52	0.054 (3)	0.064 (8)	0.059 (3)	0.021 (4)	−0.007 (2)	−0.011 (4)
C53	0.073 (4)	0.084 (9)	0.059 (3)	0.035 (5)	−0.002 (3)	−0.020 (5)
C54	0.074 (4)	0.077 (12)	0.043 (2)	0.008 (3)	−0.004 (2)	−0.016 (4)
C55	0.064 (5)	0.104 (4)	0.053 (4)	0.021 (7)	−0.013 (4)	−0.013 (5)
C56	0.048 (4)	0.081 (6)	0.049 (4)	0.012 (5)	0.003 (3)	−0.012 (4)
O54	0.104 (3)	0.148 (12)	0.059 (2)	0.039 (5)	−0.0194 (19)	−0.038 (4)
C57	0.109 (5)	0.125 (13)	0.070 (3)	0.022 (7)	−0.034 (3)	−0.025 (7)
C31	0.042 (2)	0.040 (2)	0.059 (2)	0.0033 (19)	−0.006 (2)	−0.0082 (18)
C32	0.061 (3)	0.061 (3)	0.090 (3)	−0.008 (2)	0.005 (3)	−0.012 (2)
C33	0.080 (4)	0.085 (3)	0.080 (4)	−0.001 (3)	0.029 (3)	0.002 (3)
C34	0.089 (4)	0.078 (3)	0.060 (3)	0.017 (3)	−0.007 (3)	−0.005 (2)
Cl34	0.1856 (16)	0.1630 (13)	0.0572 (8)	0.0402 (11)	−0.0065 (8)	−0.0083 (8)
C35	0.070 (3)	0.077 (3)	0.062 (3)	−0.002 (2)	−0.015 (3)	−0.010 (2)
C36	0.054 (3)	0.054 (2)	0.055 (3)	−0.007 (2)	−0.007 (2)	−0.0026 (18)
C37	0.056 (3)	0.044 (2)	0.075 (3)	0.007 (2)	−0.017 (3)	−0.017 (2)
O31	0.087 (2)	0.0683 (18)	0.0553 (17)	−0.0048 (16)	−0.0119 (16)	−0.0086 (14)
O32	0.095 (2)	0.132 (3)	0.110 (2)	−0.033 (2)	−0.040 (2)	−0.0309 (19)
O41	0.068 (2)	0.0661 (17)	0.0737 (19)	−0.0116 (16)	−0.0191 (15)	−0.0057 (14)

### 4-(4-Methoxyphenyl)piperazin-1-ium 4-chlorobenzoate monohydrate (III) Geometric parameters (Å, º)

**Table:**

N1—C2	1.481 (4)	C27—H27C	0.9600
N1—C6	1.487 (4)	C51—C56	1.376 (12)
N1—H11	1.09 (3)	C51—C52	1.40 (2)
N1—H12	0.83 (3)	C52—C53	1.378 (9)
C2—C3	1.507 (4)	C52—H52	0.9300
C2—H2A	0.9700	C53—C54	1.366 (9)
C2—H2B	0.9700	C53—H53	0.9300
C3—N4	1.461 (4)	C54—C55	1.363 (14)
C3—H3A	0.9700	C54—O54	1.373 (8)
C3—H3B	0.9700	C55—C56	1.380 (9)
N4—C51	1.40 (3)	C55—H55	0.9300
N4—C21	1.434 (12)	C56—H56	0.9300
N4—C5	1.468 (4)	O54—C57	1.401 (9)
C5—C6	1.505 (4)	C57—H57A	0.9600
C5—H5A	0.9700	C57—H57B	0.9600
C5—H5B	0.9700	C57—H57C	0.9600
C6—H6A	0.9700	C31—C36	1.376 (4)
C6—H6B	0.9700	C31—C32	1.378 (5)
C21—C26	1.376 (12)	C31—C37	1.494 (5)
C21—C22	1.40 (2)	C32—C33	1.389 (5)
C22—C23	1.378 (5)	C32—H32	0.9300
C22—H22	0.9300	C33—C34	1.372 (5)
C23—C24	1.366 (6)	C33—H33	0.9300
C23—H23	0.9300	C34—C35	1.363 (5)
C24—C25	1.363 (12)	C34—Cl34	1.731 (4)
C24—O24	1.373 (5)	C35—C36	1.379 (4)
C25—C26	1.380 (6)	C35—H35	0.9300
C25—H25	0.9300	C36—H36	0.9300
C26—H26	0.9300	C37—O32	1.233 (4)
O24—C27	1.400 (6)	C37—O31	1.265 (4)
C27—H27A	0.9600	O41—H41	0.91 (4)
C27—H27B	0.9600	O41—H42	0.94 (4)
			
C2—N1—C6	110.0 (3)	O24—C27—H27A	109.5
C2—N1—H11	110.7 (16)	O24—C27—H27B	109.5
C6—N1—H11	112.6 (15)	H27A—C27—H27B	109.5
C2—N1—H12	105 (2)	O24—C27—H27C	109.5
C6—N1—H12	114 (2)	H27A—C27—H27C	109.5
H11—N1—H12	105 (3)	H27B—C27—H27C	109.5
N1—C2—C3	110.9 (3)	C56—C51—C52	116.1 (16)
N1—C2—H2A	109.5	C56—C51—N4	125 (2)
C3—C2—H2A	109.5	C52—C51—N4	118 (4)
N1—C2—H2B	109.5	C53—C52—C51	120.5 (14)
C3—C2—H2B	109.5	C53—C52—H52	119.7
H2A—C2—H2B	108.1	C51—C52—H52	119.7
N4—C3—C2	112.4 (3)	C54—C53—C52	121.5 (11)
N4—C3—H3A	109.1	C54—C53—H53	119.2
C2—C3—H3A	109.1	C52—C53—H53	119.2
N4—C3—H3B	109.1	C55—C54—C53	118.5 (10)
C2—C3—H3B	109.1	C55—C54—O54	125.5 (13)
H3A—C3—H3B	107.9	C53—C54—O54	116.0 (12)
C51—N4—C3	118 (4)	C54—C55—C56	120.5 (12)
C21—N4—C3	113.7 (13)	C54—C55—H55	119.8
C51—N4—C5	112 (4)	C56—C55—H55	119.8
C21—N4—C5	115.8 (14)	C51—C56—C55	122.4 (12)
C3—N4—C5	111.1 (2)	C51—C56—H56	118.8
N4—C5—C6	111.8 (3)	C55—C56—H56	118.8
N4—C5—H5A	109.3	C54—O54—C57	119.2 (14)
C6—C5—H5A	109.3	O54—C57—H57A	109.5
N4—C5—H5B	109.3	O54—C57—H57B	109.5
C6—C5—H5B	109.3	H57A—C57—H57B	109.5
H5A—C5—H5B	107.9	O54—C57—H57C	109.5
N1—C6—C5	110.5 (3)	H57A—C57—H57C	109.5
N1—C6—H6A	109.6	H57B—C57—H57C	109.5
C5—C6—H6A	109.6	C36—C31—C32	118.3 (3)
N1—C6—H6B	109.6	C36—C31—C37	121.7 (4)
C5—C6—H6B	109.6	C32—C31—C37	120.0 (4)
H6A—C6—H6B	108.1	C31—C32—C33	121.0 (4)
C26—C21—C22	116.2 (8)	C31—C32—H32	119.5
C26—C21—N4	121.0 (15)	C33—C32—H32	119.5
C22—C21—N4	122.8 (12)	C34—C33—C32	119.1 (4)
C23—C22—C21	120.9 (6)	C34—C33—H33	120.4
C23—C22—H22	119.5	C32—C33—H33	120.4
C21—C22—H22	119.5	C35—C34—C33	120.7 (4)
C24—C23—C22	121.4 (5)	C35—C34—Cl34	120.7 (4)
C24—C23—H23	119.3	C33—C34—Cl34	118.6 (4)
C22—C23—H23	119.3	C34—C35—C36	119.6 (4)
C25—C24—C23	118.5 (5)	C34—C35—H35	120.2
C25—C24—O24	125.6 (6)	C36—C35—H35	120.2
C23—C24—O24	115.9 (6)	C31—C36—C35	121.3 (4)
C24—C25—C26	120.7 (8)	C31—C36—H36	119.4
C24—C25—H25	119.7	C35—C36—H36	119.4
C26—C25—H25	119.7	O32—C37—O31	123.5 (4)
C21—C26—C25	122.3 (10)	O32—C37—C31	117.7 (4)
C21—C26—H26	118.8	O31—C37—C31	118.7 (4)
C25—C26—H26	118.8	H41—O41—H42	105 (3)
C24—O24—C27	119.3 (6)		
			
C6—N1—C2—C3	−56.4 (3)	C3—N4—C51—C52	−37 (18)
N1—C2—C3—N4	55.4 (4)	C5—N4—C51—C52	−168 (11)
C2—C3—N4—C51	175 (9)	C56—C51—C52—C53	−9 (19)
C2—C3—N4—C21	173 (3)	N4—C51—C52—C53	−178 (9)
C2—C3—N4—C5	−54.1 (3)	C51—C52—C53—C54	7 (11)
C51—N4—C5—C6	−171 (8)	C52—C53—C54—C55	−1 (10)
C21—N4—C5—C6	−173 (3)	C52—C53—C54—O54	−179 (5)
C3—N4—C5—C6	54.8 (3)	C53—C54—C55—C56	−2 (14)
C2—N1—C6—C5	57.2 (3)	O54—C54—C55—C56	176 (8)
N4—C5—C6—N1	−56.8 (4)	C52—C51—C56—C55	6 (20)
C3—N4—C21—C26	169 (4)	N4—C51—C56—C55	174 (13)
C5—N4—C21—C26	38 (7)	C54—C55—C56—C51	−1 (17)
C3—N4—C21—C22	−10 (7)	C55—C54—O54—C57	13 (13)
C5—N4—C21—C22	−140 (4)	C53—C54—O54—C57	−169 (5)
C26—C21—C22—C23	3 (7)	C36—C31—C32—C33	0.8 (5)
N4—C21—C22—C23	−178 (4)	C37—C31—C32—C33	−179.2 (3)
C21—C22—C23—C24	−3 (4)	C31—C32—C33—C34	−0.3 (6)
C22—C23—C24—C25	1 (3)	C32—C33—C34—C35	−0.4 (6)
C22—C23—C24—O24	−178.6 (14)	C32—C33—C34—Cl34	179.1 (3)
C23—C24—C25—C26	1 (5)	C33—C34—C35—C36	0.5 (6)
O24—C24—C25—C26	−180 (3)	Cl34—C34—C35—C36	−179.0 (3)
C22—C21—C26—C25	−2 (7)	C32—C31—C36—C35	−0.7 (5)
N4—C21—C26—C25	180 (4)	C37—C31—C36—C35	179.3 (3)
C24—C25—C26—C21	0 (6)	C34—C35—C36—C31	0.1 (5)
C25—C24—O24—C27	−10 (4)	C36—C31—C37—O32	174.6 (3)
C23—C24—O24—C27	169.9 (15)	C32—C31—C37—O32	−5.3 (5)
C3—N4—C51—C56	155 (12)	C36—C31—C37—O31	−5.1 (5)
C5—N4—C51—C56	24 (19)	C32—C31—C37—O31	174.9 (3)

### 4-(4-Methoxyphenyl)piperazin-1-ium 4-chlorobenzoate monohydrate (III) Hydrogen-bond geometry (Å, º)

**Table:**

*D*—H···*A*	*D*—H	H···*A*	*D*···*A*	*D*—H···*A*
N1—H11···O31	1.09 (3)	1.71 (3)	2.790 (4)	176 (3)
N1—H12···O41	0.83 (3)	1.98 (3)	2.811 (4)	174 (3)
O41—H41···O32^i^	0.91 (4)	1.73 (4)	2.624 (4)	172 (4)
O41—H42···O31^ii^	0.94 (4)	1.84 (4)	2.775 (4)	170 (4)
C2—H2*B*···O31^iii^	0.97	2.52	3.467 (4)	165
C6—H6*B*···O41^i^	0.97	2.60	3.408 (4)	141
C22—H22···*Cg*1^iv^	0.93	2.89	3.631 (13)	137
C26—H26···*Cg*1^iv^	0.93	2.81	3.58 (2)	141

Symmetry codes: (i) −*x*+1, −*y*+1, −*z*+1; (ii) −*x*+2, −*y*+1, −*z*+1; (iii) −*x*+2, −*y*+2, −*z*+1; (iv) −*x*+1, −*y*+2, −*z*+1.

### 4-(4-Methoxyphenyl)piperazin-1-ium 4-bromobenzoate monohydrate (IV) Crystal data

**Table:**

C_11_H_17_N_2_O^+^·C_7_H_4_BrO_2_^−^·H_2_O	*Z* = 2
*M**_r_* = 411.28	*F*(000) = 424
Triclinic, *P*1	*D*_x_ = 1.450 Mg m^−^^3^
*a* = 6.2004 (8) Å	Mo *K*α radiation, λ = 0.71073 Å
*b* = 7.4957 (9) Å	Cell parameters from 3927 reflections
*c* = 20.440 (2) Å	θ = 2.8–27.9°
α = 85.08 (1)°	µ = 2.21 mm^−^^1^
β = 87.37 (1)°	*T* = 293 K
γ = 85.00 (1)°	Plate, colourless
*V* = 942.17 (19) Å^3^	0.48 × 0.44 × 0.16 mm

### 4-(4-Methoxyphenyl)piperazin-1-ium 4-bromobenzoate monohydrate (IV) Data collection

**Table:**

Oxford Diffraction Xcalibur with Sapphire CCD diffractometer	3818 independent reflections
Radiation source: Enhance (Mo) X-ray Source	2063 reflections with *I* > 2σ(*I*)
Graphite monochromator	*R*_int_ = 0.018
ω scans	θ_max_ = 26.6°, θ_min_ = 2.8°
Absorption correction: multi-scan (CrysAlis RED; Oxford Diffraction, 2009)	*h* = −7→7
*T*_min_ = 0.536, *T*_max_ = 0.719	*k* = −8→9
6176 measured reflections	*l* = −23→25

### 4-(4-Methoxyphenyl)piperazin-1-ium 4-bromobenzoate monohydrate (IV) Refinement

**Table:**

Refinement on *F*^2^	Primary atom site location: difference Fourier map
Least-squares matrix: full	Hydrogen site location: mixed
*R*[*F*^2^ > 2σ(*F*^2^)] = 0.068	H atoms treated by a mixture of independent and constrained refinement
*wR*(*F*^2^) = 0.197	*w* = 1/[σ^2^(*F*_o_^2^) + (0.0819*P*)^2^ + 0.9249*P*] where *P* = (*F*_o_^2^ + 2*F*_c_^2^)/3
*S* = 1.06	(Δ/σ)_max_ = 0.001
3818 reflections	Δρ_max_ = 0.94 e Å^−^^3^
265 parameters	Δρ_min_ = −0.64 e Å^−^^3^
17 restraints	

### 4-(4-Methoxyphenyl)piperazin-1-ium 4-bromobenzoate monohydrate (IV) Special details

**Table:**

Experimental. Compound (IV). IR (KBr , cm^-1^) 3319 (OH), 3001 (NH_2_), 2836 (OCH_3_), 1580 (COO), 600(CBr). . NMR (CDCl_3_) δ(^1^H) ) 3.23 (m, 4H, piperazine), 3.30 (m, 4H, piperazine), 3.77 (s, 3H, OCH_3_), 6.85 (m, 4H, methoxyphenyl), 7.51 (d, J = 8.4 Hz, 2H, bromophenyl), 7.90 (d, J = 8.4 Hz,2H, bromophenyl).
Geometry. All esds (except the esd in the dihedral angle between two l.s. planes) are estimated using the full covariance matrix. The cell esds are taken into account individually in the estimation of esds in distances, angles and torsion angles; correlations between esds in cell parameters are only used when they are defined by crystal symmetry. An approximate (isotropic) treatment of cell esds is used for estimating esds involving l.s. planes.

### 4-(4-Methoxyphenyl)piperazin-1-ium 4-bromobenzoate monohydrate (IV) Fractional atomic coordinates and isotropic or equivalent isotropic displacement parameters (Å^2^)

**Table:**

	*x*	*y*	*z*	*U*_iso_*/*U*_eq_	Occ. (<1)
N1	0.7803 (6)	0.7425 (5)	0.45428 (17)	0.0475 (9)	
H11	0.813 (7)	0.770 (6)	0.488 (2)	0.057*	
H12	0.753 (7)	0.620 (7)	0.461 (2)	0.057*	
C2	0.9709 (7)	0.7508 (6)	0.4079 (2)	0.0530 (11)	
H2A	1.0899	0.6702	0.4250	0.064*	
H2B	1.0169	0.8718	0.4034	0.064*	
C3	0.9143 (6)	0.6978 (6)	0.3414 (2)	0.0502 (10)	
H3A	1.0382	0.7086	0.3110	0.060*	
H3B	0.8808	0.5732	0.3454	0.060*	
N4	0.7286 (5)	0.8112 (4)	0.31535 (14)	0.0389 (7)	
C5	0.5420 (6)	0.8089 (6)	0.36137 (18)	0.0454 (10)	
H5A	0.4924	0.6891	0.3665	0.055*	
H5B	0.4249	0.8909	0.3438	0.055*	
C6	0.5976 (7)	0.8631 (6)	0.42775 (19)	0.0485 (10)	
H6A	0.6368	0.9862	0.4234	0.058*	
H6B	0.4723	0.8565	0.4578	0.058*	
C21	0.6845 (19)	0.784 (6)	0.2485 (4)	0.0421 (11)	0.80 (2)
C22	0.8313 (11)	0.6853 (18)	0.2086 (3)	0.066 (3)	0.80 (2)
H22	0.9604	0.6325	0.2256	0.079*	0.80 (2)
C23	0.7862 (12)	0.666 (2)	0.1442 (3)	0.084 (4)	0.80 (2)
H23	0.8895	0.6040	0.1183	0.101*	0.80 (2)
C24	0.5980 (13)	0.7331 (16)	0.1174 (4)	0.062 (3)	0.80 (2)
C25	0.4512 (15)	0.826 (5)	0.1561 (6)	0.080 (4)	0.80 (2)
H25	0.3201	0.8733	0.1390	0.096*	0.80 (2)
C26	0.4941 (16)	0.850 (3)	0.2209 (4)	0.064 (4)	0.80 (2)
H26	0.3899	0.9127	0.2462	0.077*	0.80 (2)
O24	0.5730 (16)	0.7011 (17)	0.0530 (3)	0.101 (3)	0.80 (2)
C27	0.373 (2)	0.743 (3)	0.0258 (6)	0.114 (6)	0.80 (2)
H27A	0.2623	0.6966	0.0551	0.171*	0.80 (2)
H27B	0.3717	0.6905	−0.0154	0.171*	0.80 (2)
H27C	0.3464	0.8712	0.0186	0.171*	0.80 (2)
C51	0.672 (7)	0.79 (2)	0.2512 (15)	0.0421 (11)	0.20 (2)
C52	0.838 (5)	0.786 (6)	0.2026 (13)	0.066 (3)	0.20 (2)
H52	0.9785	0.8027	0.2134	0.079*	0.20 (2)
C53	0.795 (5)	0.760 (7)	0.1384 (13)	0.084 (4)	0.20 (2)
H53	0.9078	0.7242	0.1096	0.101*	0.20 (2)
C54	0.590 (5)	0.787 (9)	0.1169 (14)	0.062 (3)	0.20 (2)
C55	0.426 (6)	0.81 (2)	0.163 (2)	0.080 (4)	0.20 (2)
H55	0.2833	0.8234	0.1492	0.096*	0.20 (2)
C56	0.466 (6)	0.818 (13)	0.2286 (19)	0.064 (4)	0.20 (2)
H56	0.3515	0.8441	0.2581	0.077*	0.20 (2)
O54	0.565 (7)	0.773 (7)	0.0512 (15)	0.101 (3)	0.20 (2)
C57	0.364 (9)	0.817 (13)	0.025 (3)	0.114 (6)	0.20 (2)
H57A	0.2541	0.7740	0.0554	0.171*	0.20 (2)
H57B	0.3582	0.7635	−0.0157	0.171*	0.20 (2)
H57C	0.3404	0.9457	0.0173	0.171*	0.20 (2)
C31	0.8064 (6)	0.7392 (5)	0.6932 (2)	0.0436 (9)	
C32	0.6590 (8)	0.6690 (7)	0.7386 (3)	0.0661 (13)	
H32	0.5388	0.6192	0.7245	0.079*	
C33	0.6890 (10)	0.6724 (8)	0.8053 (3)	0.0832 (17)	
H33	0.5888	0.6259	0.8360	0.100*	
C34	0.8682 (10)	0.7450 (8)	0.8256 (2)	0.0802 (16)	
Br34	0.90280 (17)	0.75220 (15)	0.91705 (3)	0.1491 (5)	
C35	1.0170 (9)	0.8124 (7)	0.7814 (2)	0.0683 (14)	
H35	1.1383	0.8599	0.7959	0.082*	
C36	0.9870 (7)	0.8100 (6)	0.7146 (2)	0.0500 (10)	
H36	1.0884	0.8561	0.6843	0.060*	
C37	0.7721 (7)	0.7357 (6)	0.6201 (2)	0.0528 (11)	
O31	0.8989 (6)	0.8126 (4)	0.57976 (15)	0.0631 (9)	
O32	0.6180 (7)	0.6578 (6)	0.6044 (2)	0.0999 (14)	
O41	0.7233 (6)	0.3743 (5)	0.46351 (17)	0.0633 (9)	
H41	0.620 (10)	0.353 (8)	0.445 (3)	0.095*	
H42	0.820 (11)	0.313 (9)	0.449 (3)	0.095*	

### 4-(4-Methoxyphenyl)piperazin-1-ium 4-bromobenzoate monohydrate (IV) Atomic displacement parameters (Å^2^)

**Table:**

	*U*^11^	*U*^22^	*U*^33^	*U*^12^	*U*^13^	*U*^23^
N1	0.062 (2)	0.048 (2)	0.0349 (17)	−0.0089 (18)	−0.0128 (16)	−0.0048 (16)
C2	0.044 (2)	0.067 (3)	0.049 (2)	−0.004 (2)	−0.0092 (19)	−0.004 (2)
C3	0.037 (2)	0.067 (3)	0.046 (2)	0.002 (2)	−0.0018 (18)	−0.003 (2)
N4	0.0365 (17)	0.047 (2)	0.0333 (15)	−0.0045 (14)	0.0000 (13)	−0.0017 (14)
C5	0.039 (2)	0.059 (3)	0.038 (2)	0.0005 (19)	−0.0004 (17)	−0.0020 (19)
C6	0.055 (3)	0.052 (3)	0.037 (2)	0.003 (2)	0.0016 (18)	−0.0034 (18)
C21	0.044 (3)	0.048 (3)	0.034 (2)	−0.006 (3)	0.0000 (19)	−0.002 (3)
C22	0.053 (3)	0.093 (9)	0.048 (3)	0.025 (4)	−0.009 (2)	−0.017 (4)
C23	0.076 (4)	0.122 (10)	0.049 (3)	0.036 (5)	0.001 (3)	−0.031 (5)
C24	0.072 (3)	0.079 (9)	0.036 (2)	0.009 (4)	−0.007 (2)	−0.018 (3)
C25	0.058 (4)	0.133 (10)	0.046 (4)	0.015 (7)	−0.015 (3)	−0.008 (6)
C26	0.055 (4)	0.099 (10)	0.036 (3)	0.015 (5)	−0.001 (2)	−0.015 (5)
O24	0.108 (3)	0.148 (10)	0.046 (2)	0.033 (5)	−0.019 (2)	−0.037 (4)
C27	0.108 (5)	0.181 (19)	0.058 (4)	0.008 (7)	−0.030 (4)	−0.038 (7)
C51	0.044 (3)	0.048 (3)	0.034 (2)	−0.006 (3)	0.0000 (19)	−0.002 (3)
C52	0.053 (3)	0.093 (9)	0.048 (3)	0.025 (4)	−0.009 (2)	−0.017 (4)
C53	0.076 (4)	0.122 (10)	0.049 (3)	0.036 (5)	0.001 (3)	−0.031 (5)
C54	0.072 (3)	0.079 (9)	0.036 (2)	0.009 (4)	−0.007 (2)	−0.018 (3)
C55	0.058 (4)	0.133 (10)	0.046 (4)	0.015 (7)	−0.015 (3)	−0.008 (6)
C56	0.055 (4)	0.099 (10)	0.036 (3)	0.015 (5)	−0.001 (2)	−0.015 (5)
O54	0.108 (3)	0.148 (10)	0.046 (2)	0.033 (5)	−0.019 (2)	−0.037 (4)
C57	0.108 (5)	0.181 (19)	0.058 (4)	0.008 (7)	−0.030 (4)	−0.038 (7)
C31	0.041 (2)	0.040 (2)	0.049 (2)	0.0019 (18)	−0.0051 (18)	−0.0056 (18)
C32	0.052 (3)	0.070 (3)	0.076 (3)	−0.009 (2)	0.001 (2)	−0.005 (3)
C33	0.082 (4)	0.098 (4)	0.063 (3)	−0.002 (3)	0.023 (3)	0.011 (3)
C34	0.089 (4)	0.100 (4)	0.048 (3)	0.001 (3)	−0.006 (3)	0.004 (3)
Br34	0.1950 (10)	0.2001 (11)	0.0444 (4)	0.0349 (8)	−0.0150 (4)	−0.0114 (4)
C35	0.073 (3)	0.082 (4)	0.053 (3)	−0.006 (3)	−0.021 (3)	−0.010 (3)
C36	0.049 (2)	0.056 (3)	0.046 (2)	−0.010 (2)	−0.0083 (19)	0.000 (2)
C37	0.051 (3)	0.045 (3)	0.063 (3)	0.006 (2)	−0.020 (2)	−0.012 (2)
O31	0.083 (2)	0.063 (2)	0.0446 (17)	−0.0054 (18)	−0.0124 (17)	−0.0053 (15)
O32	0.087 (3)	0.130 (4)	0.094 (3)	−0.037 (3)	−0.038 (2)	−0.024 (3)
O41	0.064 (2)	0.065 (2)	0.064 (2)	−0.0133 (17)	−0.0210 (17)	−0.0029 (17)

### 4-(4-Methoxyphenyl)piperazin-1-ium 4-bromobenzoate monohydrate (IV) Geometric parameters (Å, º)

**Table:**

N1—C6	1.479 (5)	C27—H27C	0.9600
N1—C2	1.483 (6)	C51—C56	1.369 (11)
N1—H11	0.78 (5)	C51—C52	1.40 (2)
N1—H12	0.95 (5)	C52—C53	1.383 (11)
C2—C3	1.513 (6)	C52—H52	0.9300
C2—H2A	0.9700	C53—C54	1.353 (11)
C2—H2B	0.9700	C53—H53	0.9300
C3—N4	1.463 (5)	C54—C55	1.362 (19)
C3—H3A	0.9700	C54—O54	1.374 (10)
C3—H3B	0.9700	C55—C56	1.392 (11)
N4—C51	1.40 (4)	C55—H55	0.9300
N4—C21	1.441 (10)	C56—H56	0.9300
N4—C5	1.458 (5)	O54—C57	1.383 (12)
C5—C6	1.512 (5)	C57—H57A	0.9600
C5—H5A	0.9700	C57—H57B	0.9600
C5—H5B	0.9700	C57—H57C	0.9600
C6—H6A	0.9700	C31—C32	1.377 (6)
C6—H6B	0.9700	C31—C36	1.385 (5)
C21—C26	1.367 (10)	C31—C37	1.521 (6)
C21—C22	1.399 (19)	C32—C33	1.387 (7)
C22—C23	1.380 (7)	C32—H32	0.9300
C22—H22	0.9300	C33—C34	1.375 (8)
C23—C24	1.351 (8)	C33—H33	0.9300
C23—H23	0.9300	C34—C35	1.360 (8)
C24—C25	1.362 (16)	C34—Br34	1.897 (5)
C24—O24	1.374 (6)	C35—C36	1.388 (6)
C25—C26	1.392 (8)	C35—H35	0.9300
C25—H25	0.9300	C36—H36	0.9300
C26—H26	0.9300	C37—O32	1.231 (5)
O24—C27	1.383 (8)	C37—O31	1.252 (6)
C27—H27A	0.9600	O41—H41	0.79 (6)
C27—H27B	0.9600	O41—H42	0.79 (7)
			
C6—N1—C2	109.7 (3)	O24—C27—H27A	109.5
C6—N1—H11	111 (3)	O24—C27—H27B	109.5
C2—N1—H11	108 (3)	H27A—C27—H27B	109.5
C6—N1—H12	115 (3)	O24—C27—H27C	109.5
C2—N1—H12	106 (3)	H27A—C27—H27C	109.5
H11—N1—H12	106 (4)	H27B—C27—H27C	109.5
N1—C2—C3	110.1 (3)	C56—C51—C52	115 (2)
N1—C2—H2A	109.6	C56—C51—N4	125 (3)
C3—C2—H2A	109.6	C52—C51—N4	117 (4)
N1—C2—H2B	109.6	C53—C52—C51	120.4 (19)
C3—C2—H2B	109.6	C53—C52—H52	119.8
H2A—C2—H2B	108.1	C51—C52—H52	119.8
N4—C3—C2	111.3 (3)	C54—C53—C52	120.8 (13)
N4—C3—H3A	109.4	C54—C53—H53	119.6
C2—C3—H3A	109.4	C52—C53—H53	119.6
N4—C3—H3B	109.4	C53—C54—C55	117.8 (11)
C2—C3—H3B	109.4	C53—C54—O54	116.7 (13)
H3A—C3—H3B	108.0	C55—C54—O54	125.2 (15)
C51—N4—C5	112 (2)	C54—C55—C56	121.2 (16)
C21—N4—C5	115.3 (7)	C54—C55—H55	119.4
C51—N4—C3	117 (4)	C56—C55—H55	119.4
C21—N4—C3	114.0 (11)	C51—C56—C55	121.4 (12)
C5—N4—C3	111.5 (3)	C51—C56—H56	119.3
N4—C5—C6	111.4 (3)	C55—C56—H56	119.3
N4—C5—H5A	109.4	C54—O54—C57	119.3 (17)
C6—C5—H5A	109.4	O54—C57—H57A	109.5
N4—C5—H5B	109.4	O54—C57—H57B	109.5
C6—C5—H5B	109.4	H57A—C57—H57B	109.5
H5A—C5—H5B	108.0	O54—C57—H57C	109.5
N1—C6—C5	110.0 (3)	H57A—C57—H57C	109.5
N1—C6—H6A	109.7	H57B—C57—H57C	109.5
C5—C6—H6A	109.7	C32—C31—C36	119.5 (4)
N1—C6—H6B	109.7	C32—C31—C37	120.0 (4)
C5—C6—H6B	109.7	C36—C31—C37	120.5 (4)
H6A—C6—H6B	108.2	C31—C32—C33	120.3 (5)
C26—C21—C22	116.1 (7)	C31—C32—H32	119.9
C26—C21—N4	121.7 (13)	C33—C32—H32	119.9
C22—C21—N4	122.2 (10)	C34—C33—C32	119.4 (5)
C23—C22—C21	120.6 (6)	C34—C33—H33	120.3
C23—C22—H22	119.7	C32—C33—H33	120.3
C21—C22—H22	119.7	C35—C34—C33	121.1 (5)
C24—C23—C22	122.6 (5)	C35—C34—Br34	120.3 (5)
C24—C23—H23	118.7	C33—C34—Br34	118.6 (4)
C22—C23—H23	118.7	C34—C35—C36	119.7 (5)
C23—C24—C25	117.4 (5)	C34—C35—H35	120.2
C23—C24—O24	116.5 (5)	C36—C35—H35	120.2
C25—C24—O24	126.1 (6)	C31—C36—C35	120.1 (4)
C24—C25—C26	121.1 (10)	C31—C36—H36	119.9
C24—C25—H25	119.5	C35—C36—H36	119.9
C26—C25—H25	119.5	O32—C37—O31	124.0 (5)
C21—C26—C25	122.1 (10)	O32—C37—C31	117.1 (5)
C21—C26—H26	119.0	O31—C37—C31	118.9 (4)
C25—C26—H26	119.0	H41—O41—H42	105 (6)
C24—O24—C27	119.2 (5)		
			
C6—N1—C2—C3	−58.3 (5)	C5—N4—C51—C52	−180 (9)
N1—C2—C3—N4	56.7 (5)	C3—N4—C51—C52	−49 (14)
C2—C3—N4—C51	174 (7)	C56—C51—C52—C53	−20 (15)
C2—C3—N4—C21	172.1 (16)	N4—C51—C52—C53	179 (8)
C2—C3—N4—C5	−55.2 (4)	C51—C52—C53—C54	19 (9)
C51—N4—C5—C6	−171 (8)	C52—C53—C54—C55	−10 (12)
C21—N4—C5—C6	−172.5 (19)	C52—C53—C54—O54	175 (5)
C3—N4—C5—C6	55.6 (4)	C53—C54—C55—C56	3 (19)
C2—N1—C6—C5	58.6 (4)	O54—C54—C55—C56	178 (10)
N4—C5—C6—N1	−57.4 (4)	C52—C51—C56—C55	13 (18)
C5—N4—C21—C26	36 (4)	N4—C51—C56—C55	173 (14)
C3—N4—C21—C26	166 (2)	C54—C55—C56—C51	−5 (21)
C5—N4—C21—C22	−143 (2)	C53—C54—O54—C57	−173 (6)
C3—N4—C21—C22	−12 (4)	C55—C54—O54—C57	12 (14)
C26—C21—C22—C23	3 (4)	C36—C31—C32—C33	1.2 (7)
N4—C21—C22—C23	−178 (2)	C37—C31—C32—C33	−179.8 (5)
C21—C22—C23—C24	−3 (2)	C31—C32—C33—C34	−0.5 (8)
C22—C23—C24—C25	1 (2)	C32—C33—C34—C35	−0.4 (9)
C22—C23—C24—O24	−178.9 (9)	C32—C33—C34—Br34	178.8 (4)
C23—C24—C25—C26	0 (4)	C33—C34—C35—C36	0.7 (9)
O24—C24—C25—C26	180 (2)	Br34—C34—C35—C36	−178.5 (4)
C22—C21—C26—C25	−2 (4)	C32—C31—C36—C35	−0.9 (6)
N4—C21—C26—C25	179 (3)	C37—C31—C36—C35	−179.9 (4)
C24—C25—C26—C21	1 (4)	C34—C35—C36—C31	0.0 (7)
C23—C24—O24—C27	170.6 (12)	C32—C31—C37—O32	−4.8 (6)
C25—C24—O24—C27	−9 (3)	C36—C31—C37—O32	174.2 (4)
C5—N4—C51—C56	21 (16)	C32—C31—C37—O31	174.5 (4)
C3—N4—C51—C56	151 (11)	C36—C31—C37—O31	−6.5 (6)

### 4-(4-Methoxyphenyl)piperazin-1-ium 4-bromobenzoate monohydrate (IV) Hydrogen-bond geometry (Å, º)

**Table:**

*D*—H···*A*	*D*—H	H···*A*	*D*···*A*	*D*—H···*A*
N1—H11···O31	0.78 (4)	2.03 (4)	2.805 (5)	174 (5)
N1—H12···O41	0.95 (5)	1.86 (5)	2.802 (5)	172 (4)
O41—H41···O32^i^	0.79 (6)	1.84 (6)	2.623 (6)	170 (6)
O41—H42···O31^ii^	0.79 (7)	2.00 (7)	2.772 (5)	169 (6)
C2—H2*B*···O31^iii^	0.97	2.52	3.471 (5)	166
C22—H22···*Cg*1^ii^	0.93	2.52	3.471 (5)	166
C26—H26···*Cg*1^iv^	0.93	2.84	3.58 (2)	137

Symmetry codes: (i) −*x*+1, −*y*+1, −*z*+1; (ii) −*x*+2, −*y*+1, −*z*+1; (iii) −*x*+2, −*y*+2, −*z*+1; (iv) −*x*+1, −*y*+2, −*z*+1.

### 4-(4-Methoxyphenyl)piperazin-1-ium 2-hydroxybenzoate (V) Crystal data

**Table:**

C_11_H_17_N_2_O^+^·C_7_H_5_O_3_^−^	*D*_x_ = 1.317 Mg m^−^^3^
*M**_r_* = 330.38	Mo *K*α radiation, λ = 0.71073 Å
Orthorhombic, *P*2_1_2_1_2_1_	Cell parameters from 3564 reflections
*a* = 6.5009 (8) Å	θ = 2.5–27.8°
*b* = 7.9735 (9) Å	µ = 0.09 mm^−^^1^
*c* = 32.155 (4) Å	*T* = 296 K
*V* = 1666.8 (3) Å^3^	Block, colourless
*Z* = 4	0.42 × 0.42 × 0.34 mm
*F*(000) = 704	

### 4-(4-Methoxyphenyl)piperazin-1-ium 2-hydroxybenzoate (V) Data collection

**Table:**

Oxford Diffraction Xcalibur with Sapphire CCD diffractometer	3564 independent reflections
Radiation source: Enhance (Mo) X-ray Source	2875 reflections with *I* > 2σ(*I*)
Graphite monochromator	*R*_int_ = 0.014
ω scans	θ_max_ = 27.8°, θ_min_ = 2.5°
Absorption correction: multi-scan (CrysAlis RED; Oxford Diffraction, 2009)	*h* = −6→8
*T*_min_ = 0.899, *T*_max_ = 0.969	*k* = −10→6
6249 measured reflections	*l* = −38→41

### 4-(4-Methoxyphenyl)piperazin-1-ium 2-hydroxybenzoate (V) Refinement

**Table:**

Refinement on *F*^2^	Hydrogen site location: mixed
Least-squares matrix: full	H atoms treated by a mixture of independent and constrained refinement
*R*[*F*^2^ > 2σ(*F*^2^)] = 0.041	*w* = 1/[σ^2^(*F*_o_^2^) + (0.0299*P*)^2^ + 0.3737*P*] where *P* = (*F*_o_^2^ + 2*F*_c_^2^)/3
*wR*(*F*^2^) = 0.089	(Δ/σ)_max_ < 0.001
*S* = 1.05	Δρ_max_ = 0.14 e Å^−^^3^
3564 reflections	Δρ_min_ = −0.13 e Å^−^^3^
228 parameters	Extinction correction: SHELXL, Fc^*^=kFc[1+0.001xFc^2^λ^3^/sin(2θ)]^-1/4^
0 restraints	Extinction coefficient: 0.0244 (17)
Primary atom site location: difference Fourier map	Absolute structure: Flack *x* determined using 1011 quotients [(*I*^+^)-(*I*^-^)]/[(*I*^+^)+(*I*^-^)] (Parsons *et al.*, 2013)

### 4-(4-Methoxyphenyl)piperazin-1-ium 2-hydroxybenzoate (V) Special details

**Table:**

Experimental. Compound (V). IR (KBr , cm^-1^) 3650 (OH), 3040 (NH_2_), 2835 (OCH_3_), 1571 (COO). NMR (CDCl_3_) δ(^1^H) 3.31 (m, 8H, piperazine), 3.77 (s, 3H, OCH_3_), 6.85 (m, 5H, hydroxyphenyl and methoxyphenyl), 6.92 (m, 1H, hydroxyphenyl), 7.35 (t, 1H, hydroxyphenyl), 7.87 (m, 1H, hydroxyphenyl).
Geometry. All esds (except the esd in the dihedral angle between two l.s. planes) are estimated using the full covariance matrix. The cell esds are taken into account individually in the estimation of esds in distances, angles and torsion angles; correlations between esds in cell parameters are only used when they are defined by crystal symmetry. An approximate (isotropic) treatment of cell esds is used for estimating esds involving l.s. planes.

### 4-(4-Methoxyphenyl)piperazin-1-ium 2-hydroxybenzoate (V) Fractional atomic coordinates and isotropic or equivalent isotropic displacement parameters (Å^2^)

**Table:**

	*x*	*y*	*z*	*U*_iso_*/*U*_eq_	
N1	0.5261 (3)	0.3567 (3)	0.21309 (7)	0.0468 (5)	
H11	0.616 (4)	0.369 (4)	0.2367 (9)	0.056*	
H12	0.452 (4)	0.254 (4)	0.2144 (8)	0.056*	
C2	0.6568 (4)	0.3607 (4)	0.17563 (8)	0.0488 (6)	
H2A	0.7532	0.2680	0.1765	0.059*	
H2B	0.7353	0.4642	0.1753	0.059*	
C3	0.5299 (4)	0.3490 (3)	0.13672 (8)	0.0443 (6)	
H3A	0.6189	0.3643	0.1128	0.053*	
H3B	0.4705	0.2377	0.1349	0.053*	
N4	0.3654 (3)	0.4727 (3)	0.13512 (6)	0.0381 (5)	
C5	0.2422 (4)	0.4788 (3)	0.17339 (7)	0.0426 (6)	
H5A	0.1606	0.3773	0.1755	0.051*	
H5B	0.1485	0.5733	0.1720	0.051*	
C6	0.3752 (4)	0.4953 (3)	0.21130 (8)	0.0460 (6)	
H6A	0.4472	0.6018	0.2106	0.055*	
H6B	0.2898	0.4933	0.2360	0.055*	
C21	0.2490 (4)	0.4737 (3)	0.09771 (7)	0.0373 (5)	
C22	0.3077 (4)	0.3820 (4)	0.06280 (8)	0.0515 (7)	
H22	0.4232	0.3134	0.0642	0.062*	
C23	0.1976 (5)	0.3911 (4)	0.02617 (8)	0.0544 (7)	
H23	0.2412	0.3295	0.0033	0.065*	
C24	0.0260 (4)	0.4886 (3)	0.02292 (8)	0.0460 (6)	
C25	−0.0385 (4)	0.5770 (4)	0.05738 (8)	0.0529 (7)	
H25	−0.1573	0.6418	0.0560	0.063*	
C26	0.0728 (4)	0.5697 (4)	0.09394 (8)	0.0503 (7)	
H26	0.0277	0.6312	0.1168	0.060*	
O24	−0.0704 (3)	0.4899 (3)	−0.01523 (5)	0.0638 (6)	
C27	−0.2374 (6)	0.6009 (5)	−0.02052 (10)	0.0833 (11)	
H27A	−0.2851	0.5953	−0.0487	0.125*	
H27B	−0.1938	0.7133	−0.0144	0.125*	
H27C	−0.3469	0.5697	−0.0020	0.125*	
C31	0.9904 (4)	0.4542 (3)	0.32938 (7)	0.0370 (5)	
C32	1.1705 (4)	0.3584 (3)	0.32627 (7)	0.0394 (5)	
C33	1.3127 (4)	0.3600 (4)	0.35834 (8)	0.0501 (7)	
H33	1.4324	0.2967	0.3561	0.060*	
C34	1.2782 (5)	0.4544 (4)	0.39334 (8)	0.0557 (7)	
H34	1.3746	0.4546	0.4147	0.067*	
C35	1.1023 (5)	0.5484 (4)	0.39698 (8)	0.0584 (8)	
H35	1.0790	0.6119	0.4208	0.070*	
C36	0.9613 (4)	0.5480 (3)	0.36539 (8)	0.0501 (7)	
H36	0.8427	0.6123	0.3681	0.060*	
O33	1.2074 (3)	0.2609 (2)	0.29254 (6)	0.0548 (5)	
H33A	1.082 (5)	0.275 (4)	0.2766 (10)	0.082*	
C37	0.8348 (4)	0.4561 (3)	0.29516 (8)	0.0453 (6)	
O31	0.8614 (3)	0.3519 (3)	0.26569 (6)	0.0601 (6)	
O32	0.6867 (3)	0.5531 (3)	0.29674 (7)	0.0682 (6)	

### 4-(4-Methoxyphenyl)piperazin-1-ium 2-hydroxybenzoate (V) Atomic displacement parameters (Å^2^)

**Table:**

	*U*^11^	*U*^22^	*U*^33^	*U*^12^	*U*^13^	*U*^23^
N1	0.0464 (13)	0.0489 (12)	0.0452 (12)	−0.0102 (11)	−0.0143 (11)	0.0037 (11)
C2	0.0363 (13)	0.0520 (15)	0.0581 (15)	−0.0003 (12)	−0.0058 (13)	−0.0004 (14)
C3	0.0364 (13)	0.0475 (14)	0.0489 (14)	0.0030 (12)	0.0007 (11)	−0.0024 (12)
N4	0.0353 (10)	0.0416 (11)	0.0374 (10)	0.0037 (9)	−0.0007 (8)	−0.0024 (9)
C5	0.0388 (12)	0.0494 (14)	0.0395 (12)	0.0027 (12)	0.0008 (11)	−0.0041 (11)
C6	0.0484 (14)	0.0492 (15)	0.0404 (13)	−0.0030 (13)	0.0000 (12)	−0.0022 (12)
C21	0.0396 (12)	0.0350 (12)	0.0374 (12)	−0.0018 (11)	0.0015 (10)	−0.0011 (10)
C22	0.0559 (16)	0.0543 (16)	0.0444 (14)	0.0172 (14)	0.0005 (13)	−0.0062 (13)
C23	0.0699 (19)	0.0551 (17)	0.0381 (13)	0.0109 (15)	0.0013 (14)	−0.0102 (12)
C24	0.0555 (15)	0.0431 (14)	0.0393 (13)	−0.0032 (13)	−0.0070 (12)	0.0005 (11)
C25	0.0501 (15)	0.0582 (17)	0.0503 (16)	0.0139 (14)	−0.0088 (13)	−0.0047 (13)
C26	0.0523 (15)	0.0567 (17)	0.0418 (14)	0.0153 (14)	−0.0030 (12)	−0.0121 (12)
O24	0.0793 (14)	0.0684 (14)	0.0435 (10)	0.0051 (12)	−0.0190 (10)	−0.0036 (9)
C27	0.080 (2)	0.102 (3)	0.068 (2)	0.014 (2)	−0.0331 (19)	0.000 (2)
C31	0.0382 (12)	0.0331 (12)	0.0396 (13)	−0.0030 (10)	−0.0004 (10)	0.0076 (10)
C32	0.0416 (12)	0.0353 (12)	0.0413 (13)	−0.0017 (11)	−0.0006 (12)	0.0047 (11)
C33	0.0398 (14)	0.0552 (16)	0.0554 (16)	0.0006 (13)	−0.0092 (13)	0.0093 (14)
C34	0.0586 (17)	0.0642 (18)	0.0442 (15)	−0.0139 (16)	−0.0139 (14)	0.0084 (14)
C35	0.076 (2)	0.0595 (18)	0.0401 (15)	−0.0090 (17)	0.0015 (14)	−0.0046 (13)
C36	0.0524 (15)	0.0472 (15)	0.0506 (15)	0.0064 (14)	0.0064 (13)	0.0028 (12)
O33	0.0547 (12)	0.0555 (12)	0.0542 (11)	0.0139 (10)	−0.0054 (10)	−0.0087 (9)
C37	0.0431 (14)	0.0409 (14)	0.0517 (15)	−0.0037 (13)	−0.0069 (12)	0.0127 (12)
O31	0.0639 (13)	0.0586 (12)	0.0580 (12)	0.0036 (11)	−0.0245 (10)	−0.0057 (10)
O32	0.0524 (11)	0.0730 (14)	0.0793 (14)	0.0200 (11)	−0.0115 (11)	0.0136 (11)

### 4-(4-Methoxyphenyl)piperazin-1-ium 2-hydroxybenzoate (V) Geometric parameters (Å, º)

**Table:**

N1—C2	1.474 (3)	C24—C25	1.379 (4)
N1—C6	1.479 (3)	C25—C26	1.382 (4)
N1—H11	0.96 (3)	C25—H25	0.9300
N1—H12	0.95 (3)	C26—H26	0.9300
C2—C3	1.502 (3)	O24—C27	1.411 (4)
C2—H2A	0.9700	C27—H27A	0.9600
C2—H2B	0.9700	C27—H27B	0.9600
C3—N4	1.456 (3)	C27—H27C	0.9600
C3—H3A	0.9700	C31—C36	1.391 (3)
C3—H3B	0.9700	C31—C32	1.401 (3)
N4—C21	1.421 (3)	C31—C37	1.495 (3)
N4—C5	1.469 (3)	C32—O33	1.356 (3)
C5—C6	1.500 (3)	C32—C33	1.385 (3)
C5—H5A	0.9700	C33—C34	1.372 (4)
C5—H5B	0.9700	C33—H33	0.9300
C6—H6A	0.9700	C34—C35	1.372 (4)
C6—H6B	0.9700	C34—H34	0.9300
C21—C26	1.383 (3)	C35—C36	1.368 (4)
C21—C22	1.393 (3)	C35—H35	0.9300
C22—C23	1.380 (4)	C36—H36	0.9300
C22—H22	0.9300	O33—H33A	0.97 (3)
C23—C24	1.364 (4)	C37—O32	1.236 (3)
C23—H23	0.9300	C37—O31	1.272 (3)
C24—O24	1.378 (3)		
			
C2—N1—C6	109.5 (2)	C24—C23—H23	119.3
C2—N1—H11	107.1 (16)	C22—C23—H23	119.3
C6—N1—H11	110.9 (17)	C23—C24—O24	116.4 (2)
C2—N1—H12	110.3 (16)	C23—C24—C25	118.6 (2)
C6—N1—H12	108.2 (16)	O24—C24—C25	125.0 (2)
H11—N1—H12	111 (2)	C24—C25—C26	120.2 (2)
N1—C2—C3	111.3 (2)	C24—C25—H25	119.9
N1—C2—H2A	109.4	C26—C25—H25	119.9
C3—C2—H2A	109.4	C25—C26—C21	122.1 (2)
N1—C2—H2B	109.4	C25—C26—H26	118.9
C3—C2—H2B	109.4	C21—C26—H26	118.9
H2A—C2—H2B	108.0	C24—O24—C27	117.5 (2)
N4—C3—C2	113.0 (2)	O24—C27—H27A	109.5
N4—C3—H3A	109.0	O24—C27—H27B	109.5
C2—C3—H3A	109.0	H27A—C27—H27B	109.5
N4—C3—H3B	109.0	O24—C27—H27C	109.5
C2—C3—H3B	109.0	H27A—C27—H27C	109.5
H3A—C3—H3B	107.8	H27B—C27—H27C	109.5
C21—N4—C3	115.14 (18)	C36—C31—C32	117.8 (2)
C21—N4—C5	114.77 (18)	C36—C31—C37	121.0 (2)
C3—N4—C5	113.17 (19)	C32—C31—C37	121.2 (2)
N4—C5—C6	111.69 (19)	O33—C32—C33	118.8 (2)
N4—C5—H5A	109.3	O33—C32—C31	121.2 (2)
C6—C5—H5A	109.3	C33—C32—C31	120.0 (2)
N4—C5—H5B	109.3	C34—C33—C32	120.4 (3)
C6—C5—H5B	109.3	C34—C33—H33	119.8
H5A—C5—H5B	107.9	C32—C33—H33	119.8
N1—C6—C5	110.4 (2)	C35—C34—C33	120.4 (3)
N1—C6—H6A	109.6	C35—C34—H34	119.8
C5—C6—H6A	109.6	C33—C34—H34	119.8
N1—C6—H6B	109.6	C36—C35—C34	119.6 (3)
C5—C6—H6B	109.6	C36—C35—H35	120.2
H6A—C6—H6B	108.1	C34—C35—H35	120.2
C26—C21—C22	116.5 (2)	C35—C36—C31	121.9 (3)
C26—C21—N4	121.2 (2)	C35—C36—H36	119.1
C22—C21—N4	122.2 (2)	C31—C36—H36	119.1
C23—C22—C21	121.2 (3)	C32—O33—H33A	102 (2)
C23—C22—H22	119.4	O32—C37—O31	123.0 (2)
C21—C22—H22	119.4	O32—C37—C31	120.2 (3)
C24—C23—C22	121.3 (2)	O31—C37—C31	116.7 (2)
			
C6—N1—C2—C3	−57.5 (3)	C22—C21—C26—C25	−1.0 (4)
N1—C2—C3—N4	52.5 (3)	N4—C21—C26—C25	177.7 (3)
C2—C3—N4—C21	176.2 (2)	C23—C24—O24—C27	−174.6 (3)
C2—C3—N4—C5	−49.1 (3)	C25—C24—O24—C27	5.7 (4)
C21—N4—C5—C6	−174.1 (2)	C36—C31—C32—O33	178.5 (2)
C3—N4—C5—C6	51.0 (3)	C37—C31—C32—O33	−1.6 (3)
C2—N1—C6—C5	59.7 (3)	C36—C31—C32—C33	−0.3 (3)
N4—C5—C6—N1	−56.4 (3)	C37—C31—C32—C33	179.5 (2)
C3—N4—C21—C26	171.8 (2)	O33—C32—C33—C34	−178.6 (2)
C5—N4—C21—C26	37.8 (3)	C31—C32—C33—C34	0.3 (4)
C3—N4—C21—C22	−9.6 (3)	C32—C33—C34—C35	0.0 (4)
C5—N4—C21—C22	−143.6 (2)	C33—C34—C35—C36	−0.2 (4)
C26—C21—C22—C23	1.7 (4)	C34—C35—C36—C31	0.2 (4)
N4—C21—C22—C23	−176.9 (3)	C32—C31—C36—C35	0.1 (4)
C21—C22—C23—C24	−0.8 (4)	C37—C31—C36—C35	−179.8 (2)
C22—C23—C24—O24	179.2 (3)	C36—C31—C37—O32	6.6 (4)
C22—C23—C24—C25	−1.0 (4)	C32—C31—C37—O32	−173.2 (2)
C23—C24—C25—C26	1.8 (4)	C36—C31—C37—O31	−171.9 (2)
O24—C24—C25—C26	−178.5 (3)	C32—C31—C37—O31	8.3 (3)
C24—C25—C26—C21	−0.7 (5)		

### 4-(4-Methoxyphenyl)piperazin-1-ium 2-hydroxybenzoate (V) Hydrogen-bond geometry (Å, º)

**Table:**

*D*—H···*A*	*D*—H	H···*A*	*D*···*A*	*D*—H···*A*
N1—H11···O31	0.96 (3)	1.85 (3)	2.759 (3)	156 (3)
N1—H11···O32	0.96 (3)	2.47 (3)	3.283 (3)	142 (2)
N1—H12···O32^i^	0.95 (3)	1.87 (3)	2.806 (3)	166 (2)
O33—H33*A*···O31	0.97 (3)	1.60 (3)	2.516 (3)	156 (3)
C6—H6*A*···O33^ii^	0.97	2.58	3.444 (3)	148
C2—H2*A*···*Cg*1^iii^	0.97	2.88	3.711 (3)	144
C26—H26···*Cg*1^iv^	0.93	2.87	3.642 (3)	141

Symmetry codes: (i) −*x*+1, *y*−1/2, −*z*+1/2; (ii) −*x*+2, *y*+1/2, −*z*+1/2; (iii) −*x*+2, *y*−1/2, −*z*+1/2; (iv) −*x*+1, *y*+1/2, −*z*+1/2.

### 4-(4-Methoxyphenyl)piperazin-1-ium pyridine-3-carboxylate (VI) Crystal data

**Table:**

C_11_H_17_N_2_O^+^·C_6_H_4_NO_2_^−^	*D*_x_ = 1.319 Mg m^−^^3^
*M**_r_* = 315.37	Mo *K*α radiation, λ = 0.71073 Å
Orthorhombic, *P**b**c**a*	Cell parameters from 3593 reflections
*a* = 9.2817 (7) Å	θ = 2.6–27.9°
*b* = 11.2905 (7) Å	µ = 0.09 mm^−^^1^
*c* = 30.309 (2) Å	*T* = 296 K
*V* = 3176.2 (4) Å^3^	Block, colourless
*Z* = 8	0.46 × 0.42 × 0.36 mm
*F*(000) = 1344	

### 4-(4-Methoxyphenyl)piperazin-1-ium pyridine-3-carboxylate (VI) Data collection

**Table:**

Oxford Diffraction Xcalibur with Sapphire CCD diffractometer	3593 independent reflections
Radiation source: Enhance (Mo) X-ray Source	2616 reflections with *I* > 2σ(*I*)
Graphite monochromator	*R*_int_ = 0.028
ω scans	θ_max_ = 27.9°, θ_min_ = 2.6°
Absorption correction: multi-scan (CrysAlis RED; Oxford Diffraction, 2009)	*h* = −11→11
*T*_min_ = 0.879, *T*_max_ = 0.968	*k* = −14→13
22154 measured reflections	*l* = −38→35

### 4-(4-Methoxyphenyl)piperazin-1-ium pyridine-3-carboxylate (VI) Refinement

**Table:**

Refinement on *F*^2^	Hydrogen site location: mixed
Least-squares matrix: full	H atoms treated by a mixture of independent and constrained refinement
*R*[*F*^2^ > 2σ(*F*^2^)] = 0.048	*w* = 1/[σ^2^(*F*_o_^2^) + (0.0416*P*)^2^ + 1.5726*P*] where *P* = (*F*_o_^2^ + 2*F*_c_^2^)/3
*wR*(*F*^2^) = 0.119	(Δ/σ)_max_ = 0.001
*S* = 1.03	Δρ_max_ = 0.19 e Å^−^^3^
3593 reflections	Δρ_min_ = −0.16 e Å^−^^3^
215 parameters	Extinction correction: SHELXL, Fc^*^=kFc[1+0.001xFc^2^λ^3^/sin(2θ)]^-1/4^
0 restraints	Extinction coefficient: 0.0074 (6)
Primary atom site location: difference Fourier map	

### 4-(4-Methoxyphenyl)piperazin-1-ium pyridine-3-carboxylate (VI) Special details

**Table:**

Experimental. Compound (VI). IR (KBr , cm^-1^) 3040 (NH_2_), 2829 (OCH_3_), 1584 (COO). NMR (CDCl_3_) δ(^1^H) 3.27 (m, 4H, piperazine), 3.34 (m, 4H, piperazine), 3.77 (s, 3H, OCH_3_), 6.90 (m, 4H, methoxyphenyl), 7.33 (m, 1H, nicotinate), 8.67 (m, 2H, nicotinate), 9.24 (m, 1H, nicotinate).
Geometry. All esds (except the esd in the dihedral angle between two l.s. planes) are estimated using the full covariance matrix. The cell esds are taken into account individually in the estimation of esds in distances, angles and torsion angles; correlations between esds in cell parameters are only used when they are defined by crystal symmetry. An approximate (isotropic) treatment of cell esds is used for estimating esds involving l.s. planes.

### 4-(4-Methoxyphenyl)piperazin-1-ium pyridine-3-carboxylate (VI) Fractional atomic coordinates and isotropic or equivalent isotropic displacement parameters (Å^2^)

**Table:**

	*x*	*y*	*z*	*U*_iso_*/*U*_eq_	
N1	0.66467 (17)	0.63416 (13)	0.53807 (5)	0.0405 (4)	
H12	0.711 (2)	0.5701 (18)	0.5241 (6)	0.049*	
H11	0.650 (2)	0.6962 (17)	0.5161 (6)	0.049*	
C2	0.52299 (18)	0.59306 (15)	0.55418 (6)	0.0401 (4)	
H2A	0.4642	0.5684	0.5294	0.048*	
H2B	0.4739	0.6577	0.5690	0.048*	
C3	0.54099 (18)	0.49095 (14)	0.58566 (5)	0.0368 (4)	
H3A	0.4473	0.4669	0.5967	0.044*	
H3B	0.5831	0.4242	0.5702	0.044*	
N4	0.63342 (14)	0.52403 (11)	0.62269 (4)	0.0337 (3)	
C5	0.77180 (19)	0.57192 (16)	0.60810 (6)	0.0440 (4)	
H5A	0.8273	0.5094	0.5942	0.053*	
H5B	0.8255	0.5995	0.6336	0.053*	
C6	0.7534 (2)	0.67296 (16)	0.57600 (6)	0.0479 (5)	
H6A	0.7070	0.7391	0.5907	0.058*	
H6B	0.8470	0.6990	0.5656	0.058*	
C21	0.63956 (17)	0.44215 (13)	0.65799 (5)	0.0317 (3)	
C22	0.5429 (2)	0.35039 (16)	0.66255 (6)	0.0449 (4)	
H22	0.4750	0.3379	0.6405	0.054*	
C23	0.5436 (2)	0.27603 (15)	0.69898 (6)	0.0464 (5)	
H23	0.4759	0.2155	0.7010	0.056*	
C24	0.64245 (19)	0.29057 (14)	0.73195 (5)	0.0391 (4)	
C25	0.7405 (2)	0.38118 (18)	0.72806 (6)	0.0558 (5)	
H25	0.8087	0.3926	0.7501	0.067*	
C26	0.7395 (2)	0.45523 (17)	0.69203 (6)	0.0523 (5)	
H26	0.8073	0.5157	0.6903	0.063*	
O24	0.65388 (16)	0.22083 (12)	0.76910 (4)	0.0551 (4)	
C27	0.5378 (3)	0.14351 (19)	0.77845 (7)	0.0632 (6)	
H27A	0.5581	0.0999	0.8049	0.095*	
H27B	0.4512	0.1888	0.7824	0.095*	
H27C	0.5252	0.0893	0.7544	0.095*	
N31	0.6143 (2)	1.14730 (14)	0.39733 (6)	0.0587 (5)	
C32	0.6210 (2)	1.07950 (15)	0.43309 (6)	0.0439 (4)	
H32	0.6092	1.1158	0.4604	0.053*	
C33	0.64434 (17)	0.95882 (13)	0.43243 (5)	0.0332 (4)	
C34	0.66384 (19)	0.90618 (16)	0.39174 (6)	0.0419 (4)	
H34	0.6786	0.8249	0.3896	0.050*	
C35	0.6611 (2)	0.97549 (19)	0.35436 (6)	0.0517 (5)	
H35	0.6767	0.9422	0.3267	0.062*	
C36	0.6353 (2)	1.09376 (19)	0.35863 (7)	0.0588 (6)	
H36	0.6321	1.1397	0.3332	0.071*	
C37	0.64844 (18)	0.88736 (15)	0.47434 (6)	0.0378 (4)	
O31	0.60454 (17)	0.78319 (11)	0.47188 (4)	0.0589 (4)	
O32	0.69492 (15)	0.93503 (12)	0.50849 (4)	0.0543 (4)	

### 4-(4-Methoxyphenyl)piperazin-1-ium pyridine-3-carboxylate (VI) Atomic displacement parameters (Å^2^)

**Table:**

	*U*^11^	*U*^22^	*U*^33^	*U*^12^	*U*^13^	*U*^23^
N1	0.0484 (9)	0.0353 (7)	0.0379 (8)	0.0052 (7)	0.0025 (7)	0.0083 (6)
C2	0.0387 (9)	0.0413 (9)	0.0403 (9)	0.0052 (7)	−0.0030 (7)	0.0026 (8)
C3	0.0337 (9)	0.0384 (9)	0.0383 (9)	−0.0016 (7)	−0.0021 (7)	0.0027 (7)
N4	0.0313 (7)	0.0354 (7)	0.0344 (7)	−0.0020 (6)	0.0001 (6)	0.0032 (6)
C5	0.0382 (9)	0.0509 (10)	0.0428 (9)	−0.0112 (8)	−0.0039 (8)	0.0099 (8)
C6	0.0515 (11)	0.0435 (10)	0.0488 (11)	−0.0119 (8)	−0.0021 (9)	0.0082 (8)
C21	0.0315 (8)	0.0321 (8)	0.0314 (8)	0.0024 (6)	0.0031 (6)	−0.0009 (6)
C22	0.0483 (11)	0.0452 (10)	0.0413 (10)	−0.0135 (8)	−0.0089 (8)	0.0042 (8)
C23	0.0536 (11)	0.0405 (9)	0.0452 (10)	−0.0153 (8)	0.0004 (8)	0.0044 (8)
C24	0.0465 (10)	0.0363 (8)	0.0345 (8)	0.0008 (8)	0.0049 (7)	0.0038 (7)
C25	0.0547 (12)	0.0632 (12)	0.0495 (11)	−0.0151 (10)	−0.0169 (9)	0.0159 (10)
C26	0.0487 (11)	0.0556 (11)	0.0526 (11)	−0.0201 (9)	−0.0122 (9)	0.0165 (9)
O24	0.0657 (9)	0.0559 (8)	0.0436 (7)	−0.0092 (7)	−0.0005 (6)	0.0169 (6)
C27	0.0701 (14)	0.0575 (12)	0.0620 (13)	−0.0050 (11)	0.0117 (11)	0.0232 (10)
N31	0.0794 (13)	0.0393 (8)	0.0573 (10)	−0.0007 (9)	−0.0084 (9)	0.0127 (8)
C32	0.0537 (11)	0.0356 (9)	0.0425 (10)	0.0018 (8)	−0.0021 (8)	0.0007 (8)
C33	0.0283 (8)	0.0318 (8)	0.0396 (9)	−0.0024 (6)	−0.0031 (7)	0.0036 (6)
C34	0.0434 (10)	0.0364 (9)	0.0458 (10)	0.0001 (8)	−0.0040 (8)	−0.0040 (8)
C35	0.0523 (12)	0.0664 (13)	0.0363 (10)	−0.0086 (10)	−0.0004 (8)	−0.0028 (9)
C36	0.0712 (14)	0.0595 (13)	0.0458 (11)	−0.0115 (11)	−0.0079 (10)	0.0195 (10)
C37	0.0343 (9)	0.0377 (9)	0.0415 (9)	−0.0008 (7)	−0.0032 (7)	0.0065 (7)
O31	0.0833 (11)	0.0368 (7)	0.0566 (8)	−0.0120 (7)	−0.0157 (7)	0.0144 (6)
O32	0.0670 (9)	0.0575 (8)	0.0385 (7)	−0.0166 (7)	−0.0106 (6)	0.0060 (6)

### 4-(4-Methoxyphenyl)piperazin-1-ium pyridine-3-carboxylate (VI) Geometric parameters (Å, º)

**Table:**

N1—C2	1.477 (2)	C24—C25	1.375 (3)
N1—C6	1.480 (2)	C24—O24	1.3781 (19)
N1—H12	0.94 (2)	C25—C26	1.375 (2)
N1—H11	0.97 (2)	C25—H25	0.9300
C2—C3	1.506 (2)	C26—H26	0.9300
C2—H2A	0.9700	O24—C27	1.416 (2)
C2—H2B	0.9700	C27—H27A	0.9600
C3—N4	1.461 (2)	C27—H27B	0.9600
C3—H3A	0.9700	C27—H27C	0.9600
C3—H3B	0.9700	N31—C32	1.328 (2)
N4—C21	1.4152 (19)	N31—C36	1.334 (3)
N4—C5	1.462 (2)	C32—C33	1.380 (2)
C5—C6	1.509 (2)	C32—H32	0.9300
C5—H5A	0.9700	C33—C34	1.381 (2)
C5—H5B	0.9700	C33—C37	1.505 (2)
C6—H6A	0.9700	C34—C35	1.377 (3)
C6—H6B	0.9700	C34—H34	0.9300
C21—C22	1.377 (2)	C35—C36	1.363 (3)
C21—C26	1.395 (2)	C35—H35	0.9300
C22—C23	1.387 (2)	C36—H36	0.9300
C22—H22	0.9300	C37—O32	1.244 (2)
C23—C24	1.366 (3)	C37—O31	1.247 (2)
C23—H23	0.9300		
			
C2—N1—C6	109.37 (13)	C24—C23—C22	120.82 (16)
C2—N1—H12	108.5 (12)	C24—C23—H23	119.6
C6—N1—H12	108.6 (12)	C22—C23—H23	119.6
C2—N1—H11	109.1 (11)	C23—C24—C25	118.14 (16)
C6—N1—H11	113.2 (11)	C23—C24—O24	125.48 (16)
H12—N1—H11	107.9 (15)	C25—C24—O24	116.38 (16)
N1—C2—C3	110.55 (13)	C24—C25—C26	121.05 (17)
N1—C2—H2A	109.5	C24—C25—H25	119.5
C3—C2—H2A	109.5	C26—C25—H25	119.5
N1—C2—H2B	109.5	C25—C26—C21	121.82 (17)
C3—C2—H2B	109.5	C25—C26—H26	119.1
H2A—C2—H2B	108.1	C21—C26—H26	119.1
N4—C3—C2	110.87 (13)	C24—O24—C27	117.22 (15)
N4—C3—H3A	109.5	O24—C27—H27A	109.5
C2—C3—H3A	109.5	O24—C27—H27B	109.5
N4—C3—H3B	109.5	H27A—C27—H27B	109.5
C2—C3—H3B	109.5	O24—C27—H27C	109.5
H3A—C3—H3B	108.1	H27A—C27—H27C	109.5
C21—N4—C3	115.94 (13)	H27B—C27—H27C	109.5
C21—N4—C5	115.77 (13)	C32—N31—C36	116.71 (16)
C3—N4—C5	112.21 (13)	N31—C32—C33	124.38 (17)
N4—C5—C6	112.02 (15)	N31—C32—H32	117.8
N4—C5—H5A	109.2	C33—C32—H32	117.8
C6—C5—H5A	109.2	C32—C33—C34	117.29 (16)
N4—C5—H5B	109.2	C32—C33—C37	121.41 (15)
C6—C5—H5B	109.2	C34—C33—C37	121.31 (14)
H5A—C5—H5B	107.9	C35—C34—C33	119.20 (17)
N1—C6—C5	109.88 (14)	C35—C34—H34	120.4
N1—C6—H6A	109.7	C33—C34—H34	120.4
C5—C6—H6A	109.7	C36—C35—C34	118.80 (18)
N1—C6—H6B	109.7	C36—C35—H35	120.6
C5—C6—H6B	109.7	C34—C35—H35	120.6
H6A—C6—H6B	108.2	N31—C36—C35	123.59 (18)
C22—C21—C26	116.02 (15)	N31—C36—H36	118.2
C22—C21—N4	122.74 (15)	C35—C36—H36	118.2
C26—C21—N4	121.11 (14)	O32—C37—O31	124.84 (16)
C21—C22—C23	122.14 (16)	O32—C37—C33	118.62 (15)
C21—C22—H22	118.9	O31—C37—C33	116.54 (15)
C23—C22—H22	118.9		
			
C6—N1—C2—C3	−59.85 (18)	O24—C24—C25—C26	179.32 (18)
N1—C2—C3—N4	57.18 (18)	C24—C25—C26—C21	0.1 (3)
C2—C3—N4—C21	170.15 (13)	C22—C21—C26—C25	−0.4 (3)
C2—C3—N4—C5	−53.72 (18)	N4—C21—C26—C25	175.52 (18)
C21—N4—C5—C6	−170.28 (14)	C23—C24—O24—C27	−13.4 (3)
C3—N4—C5—C6	53.52 (19)	C25—C24—O24—C27	167.31 (18)
C2—N1—C6—C5	58.59 (19)	C36—N31—C32—C33	−1.7 (3)
N4—C5—C6—N1	−55.7 (2)	N31—C32—C33—C34	0.9 (3)
C3—N4—C21—C22	−13.9 (2)	N31—C32—C33—C37	−179.08 (17)
C5—N4—C21—C22	−148.45 (17)	C32—C33—C34—C35	0.9 (3)
C3—N4—C21—C26	170.48 (16)	C37—C33—C34—C35	−179.12 (16)
C5—N4—C21—C26	35.9 (2)	C33—C34—C35—C36	−1.8 (3)
C26—C21—C22—C23	0.7 (3)	C32—N31—C36—C35	0.7 (3)
N4—C21—C22—C23	−175.17 (16)	C34—C35—C36—N31	1.0 (3)
C21—C22—C23—C24	−0.6 (3)	C32—C33—C37—O32	−32.1 (2)
C22—C23—C24—C25	0.3 (3)	C34—C33—C37—O32	147.86 (17)
C22—C23—C24—O24	−178.99 (17)	C32—C33—C37—O31	148.09 (18)
C23—C24—C25—C26	0.0 (3)	C34—C33—C37—O31	−31.9 (2)

### 4-(4-Methoxyphenyl)piperazin-1-ium pyridine-3-carboxylate (VI) Hydrogen-bond geometry (Å, º)

**Table:**

*D*—H···*A*	*D*—H	H···*A*	*D*···*A*	*D*—H···*A*
N1—H11···O31	0.976 (19)	1.714 (19)	2.677 (2)	168.2 (18)
N1—H12···O32^i^	0.94 (2)	1.82 (2)	2.749 (2)	168.3 (17)
C2—H2*B*···N31^ii^	0.97	2.56	3.518 (2)	169
C36—H36···O24^iii^	0.93	2.51	3.432 (2)	172
C3—H3*A*···*Cg*1^iv^	0.97	2.97	3.775 (2)	156

Symmetry codes: (i) −*x*+3/2, *y*−1/2, *z*; (ii) −*x*+1, −*y*+2, −*z*+1; (iii) *x*, −*y*+3/2, *z*−1/2; (iv) *x*−1/2, −*y*+3/2, −*z*+1.

### 4-(4-Methoxyphenyl)piperazin-1-ium 2-hydroxy-3,5-dinitrobenzoate (VII) Crystal data

**Table:**

C_7_H_3_N_2_O_7_^+^·C_11_H_17_N_2_O^−^	*F*(000) = 880
*M**_r_* = 420.38	*D*_x_ = 1.478 Mg m^−^^3^
Monoclinic, *P*2_1_/*c*	Mo *K*α radiation, λ = 0.71073 Å
*a* = 7.5500 (9) Å	Cell parameters from 4078 reflections
*b* = 7.6489 (9) Å	θ = 2.7–28.0°
*c* = 32.719 (6) Å	µ = 0.12 mm^−^^1^
β = 91.30 (1)°	*T* = 296 K
*V* = 1889.0 (5) Å^3^	Block, colourless
*Z* = 4	0.18 × 0.12 × 0.06 mm

### 4-(4-Methoxyphenyl)piperazin-1-ium 2-hydroxy-3,5-dinitrobenzoate (VII) Data collection

**Table:**

Oxford Diffraction Xcalibur with Sapphire CCD diffractometer	4074 independent reflections
Radiation source: Enhance (Mo) X-ray Source	2003 reflections with *I* > 2σ(*I*)
Graphite monochromator	*R*_int_ = 0.038
ω scans	θ_max_ = 28.0°, θ_min_ = 2.7°
Absorption correction: multi-scan (CrysAlis RED; Oxford Diffraction, 2009)	*h* = −9→5
*T*_min_ = 0.916, *T*_max_ = 0.993	*k* = −9→9
8215 measured reflections	*l* = −42→41

### 4-(4-Methoxyphenyl)piperazin-1-ium 2-hydroxy-3,5-dinitrobenzoate (VII) Refinement

**Table:**

Refinement on *F*^2^	Primary atom site location: difference Fourier map
Least-squares matrix: full	Hydrogen site location: mixed
*R*[*F*^2^ > 2σ(*F*^2^)] = 0.066	H atoms treated by a mixture of independent and constrained refinement
*wR*(*F*^2^) = 0.128	*w* = 1/[σ^2^(*F*_o_^2^) + (0.0413*P*)^2^ + 0.4471*P*] where *P* = (*F*_o_^2^ + 2*F*_c_^2^)/3
*S* = 1.03	(Δ/σ)_max_ < 0.001
4074 reflections	Δρ_max_ = 0.22 e Å^−^^3^
281 parameters	Δρ_min_ = −0.23 e Å^−^^3^
0 restraints	

### 4-(4-Methoxyphenyl)piperazin-1-ium 2-hydroxy-3,5-dinitrobenzoate (VII) Special details

**Table:**

Experimental. Compound (VII). IR (KBr , cm^-1^) 3084 (NH_2_), 2834 (OCH_3_), 1568 (COO), 1499 (NO_2_). NMR (CDCl_3_) δ(^1^H) 3.05 (m, 4H, piperazine), 3.37 (m, 4H, piperazine), 3.77 (s, 3H, OCH_3_), 6.85 (m, 4H, methoxyphenyl), 7.52 (s, 1H, 3,5-dinitrosalicylate), 8.09 (s, 1H, 3,5-dinitrosalicylate), 8.99 (s, 1H, 3,5-dinitrosalicylate).
Geometry. All esds (except the esd in the dihedral angle between two l.s. planes) are estimated using the full covariance matrix. The cell esds are taken into account individually in the estimation of esds in distances, angles and torsion angles; correlations between esds in cell parameters are only used when they are defined by crystal symmetry. An approximate (isotropic) treatment of cell esds is used for estimating esds involving l.s. planes.

### 4-(4-Methoxyphenyl)piperazin-1-ium 2-hydroxy-3,5-dinitrobenzoate (VII) Fractional atomic coordinates and isotropic or equivalent isotropic displacement parameters (Å^2^)

**Table:**

	*x*	*y*	*z*	*U*_iso_*/*U*_eq_	
N1	0.6215 (3)	0.5723 (4)	0.61103 (8)	0.0465 (7)	
H11	0.703 (4)	0.587 (4)	0.5906 (9)	0.056*	
H12	0.533 (4)	0.494 (4)	0.6028 (9)	0.056*	
C2	0.5349 (4)	0.7433 (4)	0.61776 (9)	0.0531 (9)	
H2A	0.4783	0.7826	0.5925	0.064*	
H2B	0.6235	0.8292	0.6258	0.064*	
C3	0.3982 (4)	0.7294 (4)	0.65055 (9)	0.0489 (8)	
H3A	0.3504	0.8446	0.6559	0.059*	
H3B	0.3016	0.6558	0.6407	0.059*	
N4	0.4712 (3)	0.6567 (3)	0.68854 (7)	0.0399 (6)	
C5	0.5669 (4)	0.4924 (4)	0.68187 (9)	0.0483 (8)	
H5A	0.4832	0.4024	0.6734	0.058*	
H5B	0.6233	0.4554	0.7074	0.058*	
C6	0.7057 (4)	0.5118 (4)	0.64972 (8)	0.0505 (8)	
H6A	0.7947	0.5955	0.6588	0.061*	
H6B	0.7637	0.4003	0.6454	0.061*	
C21	0.3501 (4)	0.6515 (4)	0.72152 (9)	0.0382 (7)	
C22	0.1878 (4)	0.7370 (4)	0.72002 (10)	0.0510 (8)	
H22	0.1538	0.7966	0.6963	0.061*	
C23	0.0745 (4)	0.7358 (4)	0.75303 (10)	0.0522 (8)	
H23	−0.0327	0.7954	0.7512	0.063*	
C24	0.1201 (4)	0.6473 (4)	0.78814 (9)	0.0455 (8)	
C25	0.2807 (4)	0.5624 (4)	0.79002 (9)	0.0547 (9)	
H25	0.3138	0.5028	0.8138	0.066*	
C26	0.3934 (4)	0.5636 (4)	0.75758 (9)	0.0499 (8)	
H26	0.5008	0.5044	0.7598	0.060*	
O24	0.0184 (3)	0.6354 (3)	0.82246 (6)	0.0640 (7)	
C27	−0.1485 (4)	0.7203 (5)	0.82152 (11)	0.0745 (11)	
H27A	−0.2061	0.7017	0.8470	0.112*	
H27B	−0.2206	0.6733	0.7996	0.112*	
H27C	−0.1318	0.8433	0.8173	0.112*	
C31	0.8836 (3)	0.6893 (4)	0.46865 (8)	0.0355 (7)	
C32	0.8864 (3)	0.7026 (4)	0.51247 (9)	0.0374 (7)	
C33	1.0424 (3)	0.7812 (4)	0.52995 (8)	0.0352 (7)	
C34	1.1812 (3)	0.8371 (4)	0.50706 (8)	0.0372 (7)	
H34	1.2797	0.8892	0.5195	0.045*	
C35	1.1728 (3)	0.8150 (4)	0.46526 (9)	0.0368 (7)	
C36	1.0252 (3)	0.7425 (4)	0.44590 (9)	0.0383 (7)	
H36	1.0217	0.7298	0.4176	0.046*	
C37	0.7243 (4)	0.6189 (4)	0.44690 (10)	0.0446 (8)	
O31	0.7120 (3)	0.6063 (3)	0.40978 (6)	0.0609 (7)	
O32	0.5931 (2)	0.5689 (3)	0.46961 (7)	0.0626 (7)	
H32	0.638 (4)	0.590 (5)	0.4996 (12)	0.094*	
O33	0.7553 (2)	0.6499 (3)	0.53332 (6)	0.0530 (6)	
N33	1.0576 (3)	0.8102 (4)	0.57403 (8)	0.0481 (7)	
O34	0.9695 (3)	0.7215 (4)	0.59704 (7)	0.0868 (9)	
O35	1.1628 (3)	0.9203 (3)	0.58660 (6)	0.0627 (7)	
N35	1.3207 (3)	0.8725 (3)	0.44092 (9)	0.0492 (7)	
O36	1.4399 (3)	0.9552 (3)	0.45754 (7)	0.0715 (7)	
O37	1.3199 (3)	0.8357 (3)	0.40434 (7)	0.0687 (7)	

### 4-(4-Methoxyphenyl)piperazin-1-ium 2-hydroxy-3,5-dinitrobenzoate (VII) Atomic displacement parameters (Å^2^)

**Table:**

	*U*^11^	*U*^22^	*U*^33^	*U*^12^	*U*^13^	*U*^23^
N1	0.0429 (15)	0.061 (2)	0.0360 (15)	−0.0125 (14)	0.0016 (11)	−0.0055 (14)
C2	0.064 (2)	0.053 (2)	0.0417 (19)	−0.0007 (17)	0.0004 (16)	0.0075 (17)
C3	0.0565 (19)	0.053 (2)	0.0371 (18)	0.0086 (16)	0.0021 (15)	0.0053 (16)
N4	0.0438 (13)	0.0416 (16)	0.0342 (14)	0.0040 (11)	−0.0003 (11)	0.0033 (12)
C5	0.0505 (18)	0.055 (2)	0.0397 (19)	0.0108 (16)	0.0033 (14)	0.0063 (16)
C6	0.0477 (18)	0.066 (2)	0.0374 (18)	0.0033 (16)	−0.0019 (14)	−0.0007 (17)
C21	0.0430 (16)	0.0340 (18)	0.0375 (17)	−0.0001 (13)	−0.0035 (13)	−0.0012 (14)
C22	0.0511 (19)	0.057 (2)	0.045 (2)	0.0118 (16)	0.0001 (15)	0.0135 (17)
C23	0.0480 (18)	0.053 (2)	0.056 (2)	0.0132 (15)	0.0034 (16)	0.0039 (18)
C24	0.0482 (18)	0.045 (2)	0.044 (2)	0.0021 (15)	0.0057 (15)	−0.0026 (16)
C25	0.062 (2)	0.064 (2)	0.0382 (19)	0.0154 (18)	−0.0005 (15)	0.0126 (18)
C26	0.0490 (18)	0.059 (2)	0.0417 (19)	0.0167 (16)	−0.0010 (15)	0.0055 (17)
O24	0.0614 (15)	0.0814 (18)	0.0497 (14)	0.0129 (12)	0.0142 (11)	0.0028 (13)
C27	0.057 (2)	0.096 (3)	0.071 (3)	0.014 (2)	0.0207 (19)	−0.007 (2)
C31	0.0344 (15)	0.0352 (17)	0.0366 (17)	−0.0028 (13)	−0.0008 (13)	−0.0002 (14)
C32	0.0318 (15)	0.0367 (18)	0.0437 (18)	0.0004 (13)	0.0011 (13)	0.0046 (15)
C33	0.0367 (16)	0.0371 (18)	0.0315 (16)	0.0003 (13)	−0.0022 (13)	0.0003 (14)
C34	0.0352 (15)	0.0340 (18)	0.0422 (18)	−0.0016 (13)	−0.0053 (13)	−0.0020 (15)
C35	0.0316 (15)	0.0345 (17)	0.0443 (19)	−0.0064 (13)	0.0036 (13)	0.0017 (15)
C36	0.0405 (16)	0.0355 (17)	0.0388 (17)	−0.0019 (13)	0.0018 (13)	−0.0015 (14)
C37	0.0397 (17)	0.048 (2)	0.046 (2)	−0.0086 (14)	−0.0020 (15)	0.0005 (16)
O31	0.0570 (13)	0.0868 (19)	0.0389 (14)	−0.0254 (12)	−0.0012 (10)	−0.0093 (13)
O32	0.0429 (13)	0.099 (2)	0.0455 (13)	−0.0297 (12)	−0.0024 (10)	0.0073 (14)
O33	0.0396 (12)	0.0793 (17)	0.0404 (13)	−0.0153 (11)	0.0032 (10)	0.0039 (12)
N33	0.0381 (14)	0.063 (2)	0.0424 (17)	0.0001 (13)	−0.0041 (12)	0.0013 (15)
O34	0.0712 (16)	0.146 (3)	0.0427 (15)	−0.0443 (16)	0.0011 (12)	0.0102 (16)
O35	0.0688 (15)	0.0690 (17)	0.0495 (14)	−0.0132 (13)	−0.0138 (11)	−0.0063 (13)
N35	0.0460 (16)	0.0512 (18)	0.0505 (18)	−0.0110 (13)	0.0022 (13)	0.0012 (15)
O36	0.0604 (14)	0.092 (2)	0.0616 (16)	−0.0425 (13)	0.0006 (12)	−0.0031 (14)
O37	0.0643 (15)	0.095 (2)	0.0474 (15)	−0.0258 (13)	0.0132 (11)	−0.0123 (15)

### 4-(4-Methoxyphenyl)piperazin-1-ium 2-hydroxy-3,5-dinitrobenzoate (VII) Geometric parameters (Å, º)

**Table:**

N1—C6	1.478 (4)	C25—H25	0.9300
N1—C2	1.481 (4)	C26—H26	0.9300
N1—H11	0.93 (3)	O24—C27	1.417 (3)
N1—H12	0.93 (3)	C27—H27A	0.9600
C2—C3	1.509 (4)	C27—H27B	0.9600
C2—H2A	0.9700	C27—H27C	0.9600
C2—H2B	0.9700	C31—C36	1.378 (4)
C3—N4	1.458 (3)	C31—C32	1.437 (4)
C3—H3A	0.9700	C31—C37	1.485 (4)
C3—H3B	0.9700	C32—O33	1.280 (3)
N4—C21	1.431 (3)	C32—C33	1.430 (4)
N4—C5	1.469 (3)	C33—C34	1.370 (3)
C5—C6	1.509 (4)	C33—N33	1.461 (3)
C5—H5A	0.9700	C34—C35	1.378 (4)
C5—H5B	0.9700	C34—H34	0.9300
C6—H6A	0.9700	C35—C36	1.385 (4)
C6—H6B	0.9700	C35—N35	1.455 (3)
C21—C22	1.389 (4)	C36—H36	0.9300
C21—C26	1.391 (4)	C37—O31	1.220 (3)
C22—C23	1.393 (4)	C37—O32	1.309 (3)
C22—H22	0.9300	O32—H32	1.04 (4)
C23—C24	1.371 (4)	N33—O34	1.222 (3)
C23—H23	0.9300	N33—O35	1.223 (3)
C24—C25	1.376 (4)	N35—O36	1.218 (3)
C24—O24	1.378 (3)	N35—O37	1.229 (3)
C25—C26	1.375 (4)		
			
C6—N1—C2	109.4 (2)	C25—C24—O24	116.1 (3)
C6—N1—H11	112.1 (18)	C26—C25—C24	121.5 (3)
C2—N1—H11	107.5 (18)	C26—C25—H25	119.2
C6—N1—H12	109.6 (18)	C24—C25—H25	119.2
C2—N1—H12	107.2 (18)	C25—C26—C21	121.3 (3)
H11—N1—H12	111 (3)	C25—C26—H26	119.3
N1—C2—C3	110.8 (2)	C21—C26—H26	119.3
N1—C2—H2A	109.5	C24—O24—C27	117.6 (3)
C3—C2—H2A	109.5	O24—C27—H27A	109.5
N1—C2—H2B	109.5	O24—C27—H27B	109.5
C3—C2—H2B	109.5	H27A—C27—H27B	109.5
H2A—C2—H2B	108.1	O24—C27—H27C	109.5
N4—C3—C2	112.4 (2)	H27A—C27—H27C	109.5
N4—C3—H3A	109.1	H27B—C27—H27C	109.5
C2—C3—H3A	109.1	C36—C31—C32	121.6 (2)
N4—C3—H3B	109.1	C36—C31—C37	118.6 (3)
C2—C3—H3B	109.1	C32—C31—C37	119.8 (2)
H3A—C3—H3B	107.8	O33—C32—C33	124.0 (3)
C21—N4—C3	114.7 (2)	O33—C32—C31	121.0 (2)
C21—N4—C5	114.4 (2)	C33—C32—C31	115.0 (2)
C3—N4—C5	112.2 (2)	C34—C33—C32	123.1 (3)
N4—C5—C6	111.8 (3)	C34—C33—N33	116.7 (2)
N4—C5—H5A	109.3	C32—C33—N33	120.3 (2)
C6—C5—H5A	109.3	C33—C34—C35	119.0 (2)
N4—C5—H5B	109.3	C33—C34—H34	120.5
C6—C5—H5B	109.3	C35—C34—H34	120.5
H5A—C5—H5B	107.9	C34—C35—C36	121.5 (2)
N1—C6—C5	109.6 (2)	C34—C35—N35	119.2 (2)
N1—C6—H6A	109.7	C36—C35—N35	119.4 (3)
C5—C6—H6A	109.7	C31—C36—C35	119.8 (3)
N1—C6—H6B	109.7	C31—C36—H36	120.1
C5—C6—H6B	109.7	C35—C36—H36	120.1
H6A—C6—H6B	108.2	O31—C37—O32	120.1 (3)
C22—C21—C26	116.6 (3)	O31—C37—C31	123.2 (3)
C22—C21—N4	122.6 (3)	O32—C37—C31	116.7 (3)
C26—C21—N4	120.8 (2)	C37—O32—H32	104.6 (18)
C21—C22—C23	121.8 (3)	C32—O33—H32	99.0 (13)
C21—C22—H22	119.1	O34—N33—O35	122.3 (3)
C23—C22—H22	119.1	O34—N33—C33	119.5 (3)
C24—C23—C22	120.4 (3)	O35—N33—C33	118.2 (3)
C24—C23—H23	119.8	O36—N35—O37	122.8 (2)
C22—C23—H23	119.8	O36—N35—C35	118.8 (3)
C23—C24—C25	118.4 (3)	O37—N35—C35	118.5 (2)
C23—C24—O24	125.6 (3)		
			
C6—N1—C2—C3	−58.5 (3)	C36—C31—C32—C33	2.6 (4)
N1—C2—C3—N4	54.3 (3)	C37—C31—C32—C33	−176.6 (2)
C2—C3—N4—C21	176.0 (2)	O33—C32—C33—C34	−179.8 (3)
C2—C3—N4—C5	−51.2 (3)	C31—C32—C33—C34	−1.3 (4)
C21—N4—C5—C6	−174.0 (2)	O33—C32—C33—N33	−1.6 (4)
C3—N4—C5—C6	53.0 (3)	C31—C32—C33—N33	176.9 (3)
C2—N1—C6—C5	60.0 (3)	C32—C33—C34—C35	−0.9 (4)
N4—C5—C6—N1	−57.5 (3)	N33—C33—C34—C35	−179.2 (2)
C3—N4—C21—C22	−11.2 (4)	C33—C34—C35—C36	2.0 (4)
C5—N4—C21—C22	−142.9 (3)	C33—C34—C35—N35	−179.4 (2)
C3—N4—C21—C26	170.9 (3)	C32—C31—C36—C35	−1.7 (4)
C5—N4—C21—C26	39.2 (4)	C37—C31—C36—C35	177.5 (3)
C26—C21—C22—C23	0.4 (5)	C34—C35—C36—C31	−0.6 (4)
N4—C21—C22—C23	−177.6 (3)	N35—C35—C36—C31	−179.3 (2)
C21—C22—C23—C24	−0.8 (5)	C36—C31—C37—O31	−0.3 (5)
C22—C23—C24—C25	0.9 (5)	C32—C31—C37—O31	179.0 (3)
C22—C23—C24—O24	−179.3 (3)	C36—C31—C37—O32	179.6 (3)
C23—C24—C25—C26	−0.6 (5)	C32—C31—C37—O32	−1.1 (4)
O24—C24—C25—C26	179.5 (3)	C34—C33—N33—O34	−158.4 (3)
C24—C25—C26—C21	0.3 (5)	C32—C33—N33—O34	23.3 (4)
C22—C21—C26—C25	−0.2 (5)	C34—C33—N33—O35	19.6 (4)
N4—C21—C26—C25	177.9 (3)	C32—C33—N33—O35	−158.7 (3)
C23—C24—O24—C27	0.6 (5)	C34—C35—N35—O36	−7.6 (4)
C25—C24—O24—C27	−179.6 (3)	C36—C35—N35—O36	171.1 (3)
C36—C31—C32—O33	−178.8 (3)	C34—C35—N35—O37	172.5 (3)
C37—C31—C32—O33	2.0 (4)	C36—C35—N35—O37	−8.8 (4)

### 4-(4-Methoxyphenyl)piperazin-1-ium 2-hydroxy-3,5-dinitrobenzoate (VII) Hydrogen-bond geometry (Å, º)

**Table:**

*D*—H···*A*	*D*—H	H···*A*	*D*···*A*	*D*—H···*A*
O32—H32···O33	1.04 (4)	1.47 (4)	2.472 (3)	158 (3)
N1—H11···O33	0.93 (3)	1.98 (3)	2.820 (3)	150 (3)
N1—H11···O34	0.93 (3)	2.27 (3)	2.910 (3)	126 (2)
N1—H12···O31^i^	0.93 (3)	2.04 (3)	2.931 (3)	160 (3)
N1—H12···O32^i^	0.93 (3)	2.58 (3)	3.250 (3)	129 (2)
C34—H34···O36^ii^	0.93	2.53	3.449 (3)	171
C5—H5*B*···*Cg*2^iii^	0.97	2.84	3.639 (3)	140

Symmetry codes: (i) −*x*+1, −*y*+1, −*z*+1; (ii) −*x*+3, −*y*+2, −*z*+1; (iii) −*x*+1, *y*−1/2, −*z*+3/2.

### 4-(4-Methoxyphenyl)piperazin-1-ium hydrogensuccinate (VIII) Crystal data

**Table:**

C_11_H_17_N_2_O^+^·C_4_H_5_O_4_^−^	*D*_x_ = 1.344 Mg m^−^^3^
*M**_r_* = 310.35	Mo *K*α radiation, λ = 0.71073 Å
Orthorhombic, *P**n**a*2_1_	Cell parameters from 2423 reflections
*a* = 9.3225 (9) Å	θ = 2.6–27.8°
*b* = 28.261 (3) Å	µ = 0.10 mm^−^^1^
*c* = 5.8228 (8) Å	*T* = 296 K
*V* = 1534.1 (3) Å^3^	Block, colourless
*Z* = 4	0.44 × 0.42 × 0.24 mm
*F*(000) = 664	

### 4-(4-Methoxyphenyl)piperazin-1-ium hydrogensuccinate (VIII) Data collection

**Table:**

Oxford Diffraction Xcalibur with Sapphire CCD diffractometer	2419 independent reflections
Radiation source: Enhance (Mo) X-ray Source	2053 reflections with *I* > 2σ(*I*)
Graphite monochromator	*R*_int_ = 0.018
ω scans	θ_max_ = 27.5°, θ_min_ = 2.6°
Absorption correction: multi-scan (CrysAlis RED; Oxford Diffraction, 2009)	*h* = −6→11
*T*_min_ = 0.816, *T*_max_ = 0.976	*k* = −36→27
5828 measured reflections	*l* = −7→5

### 4-(4-Methoxyphenyl)piperazin-1-ium hydrogensuccinate (VIII) Refinement

**Table:**

Refinement on *F*^2^	Primary atom site location: difference Fourier map
Least-squares matrix: full	Hydrogen site location: mixed
*R*[*F*^2^ > 2σ(*F*^2^)] = 0.043	H atoms treated by a mixture of independent and constrained refinement
*wR*(*F*^2^) = 0.104	*w* = 1/[σ^2^(*F*_o_^2^) + (0.0459*P*)^2^ + 0.356*P*] where *P* = (*F*_o_^2^ + 2*F*_c_^2^)/3
*S* = 1.14	(Δ/σ)_max_ < 0.001
2419 reflections	Δρ_max_ = 0.16 e Å^−^^3^
233 parameters	Δρ_min_ = −0.24 e Å^−^^3^
16 restraints	Absolute structure: Flack *x* determined using 460 quotients [(*I*^+^)-(*I*^-^)]/[(*I*^+^)+(*I*^-^)] (Parsons *et al.*, 2013)

### 4-(4-Methoxyphenyl)piperazin-1-ium hydrogensuccinate (VIII) Special details

**Table:**

Experimental. Compound (VIII). IR (KBr , cm^-1^) 3135 (NH_2_), 2836 (OCH_3_), 1562 (COO). NMR (CDCl_3_) δ(^1^H) ) 2.66 (s, 4H, succinate), 3.32 (m, 4H, piperazine), 3.35 (m, 4H, piperazine), 3.77 (s, 3H, OCH_3_), 6.90 (m, 4H, methoxyphenyl).
Geometry. All esds (except the esd in the dihedral angle between two l.s. planes) are estimated using the full covariance matrix. The cell esds are taken into account individually in the estimation of esds in distances, angles and torsion angles; correlations between esds in cell parameters are only used when they are defined by crystal symmetry. An approximate (isotropic) treatment of cell esds is used for estimating esds involving l.s. planes.

### 4-(4-Methoxyphenyl)piperazin-1-ium hydrogensuccinate (VIII) Fractional atomic coordinates and isotropic or equivalent isotropic displacement parameters (Å^2^)

**Table:**

	*x*	*y*	*z*	*U*_iso_*/*U*_eq_	Occ. (<1)
N1	0.5210 (3)	0.66867 (8)	0.5428 (5)	0.0395 (6)	
H11	0.580 (3)	0.6921 (11)	0.556 (7)	0.047*	
H12	0.435 (3)	0.6801 (10)	0.623 (7)	0.047*	
C2	0.5853 (3)	0.62541 (9)	0.6443 (7)	0.0435 (7)	
H2A	0.6135	0.6317	0.8018	0.052*	
H2B	0.6704	0.6167	0.5586	0.052*	
C3	0.4786 (3)	0.58515 (9)	0.6388 (6)	0.0414 (7)	
H3A	0.5238	0.5568	0.6992	0.050*	
H3B	0.3981	0.5929	0.7374	0.050*	
N4	0.4259 (2)	0.57556 (7)	0.4075 (5)	0.0347 (5)	
C5	0.3676 (3)	0.61875 (9)	0.3026 (6)	0.0439 (7)	
H5A	0.2824	0.6284	0.3859	0.053*	
H5B	0.3397	0.6120	0.1454	0.053*	
C6	0.4745 (3)	0.65878 (10)	0.3038 (6)	0.0479 (8)	
H6A	0.5570	0.6503	0.2110	0.058*	
H6B	0.4312	0.6869	0.2380	0.058*	
C21	0.3353 (3)	0.53502 (9)	0.3862 (5)	0.0331 (6)	
C22	0.3336 (3)	0.49886 (9)	0.5496 (6)	0.0394 (7)	
H22	0.3926	0.5011	0.6779	0.047*	
C23	0.2451 (3)	0.45967 (10)	0.5241 (6)	0.0440 (7)	
H23	0.2456	0.4361	0.6354	0.053*	
C24	0.1567 (3)	0.45534 (9)	0.3356 (6)	0.0407 (7)	
C25	0.1589 (3)	0.48988 (10)	0.1703 (7)	0.0484 (8)	
H25	0.1013	0.4870	0.0406	0.058*	
C26	0.2469 (3)	0.52924 (10)	0.1961 (6)	0.0464 (8)	
H26	0.2466	0.5524	0.0826	0.056*	
O24	0.0728 (2)	0.41532 (8)	0.3280 (5)	0.0620 (8)	
C27	−0.0230 (4)	0.41098 (13)	0.1399 (9)	0.0729 (12)	
H27A	−0.0790	0.3827	0.1572	0.109*	
H27B	0.0307	0.4094	−0.0005	0.109*	
H27C	−0.0856	0.4379	0.1358	0.109*	
C31	0.7061 (15)	0.7714 (3)	0.797 (3)	0.0296 (18)	0.660 (15)
O31	0.7173 (15)	0.7375 (5)	0.655 (3)	0.0463 (18)	0.660 (15)
O32	0.7811 (18)	0.8079 (5)	0.790 (4)	0.0443 (15)	0.660 (15)
C32	0.5909 (9)	0.7663 (3)	0.9790 (16)	0.0375 (16)	0.660 (15)
H32A	0.6331	0.7517	1.1138	0.045*	0.660 (15)
H32B	0.5175	0.7450	0.9216	0.045*	0.660 (15)
C33	0.5200 (11)	0.8123 (3)	1.0509 (19)	0.056 (3)	0.660 (15)
H33A	0.5933	0.8339	1.1050	0.067*	0.660 (15)
H33B	0.4747	0.8265	0.9178	0.067*	0.660 (15)
C34	0.4093 (16)	0.8060 (6)	1.237 (3)	0.0468 (19)	0.660 (15)
O33	0.3172 (11)	0.8361 (3)	1.279 (2)	0.086 (3)	0.660 (15)
O34	0.4253 (19)	0.7688 (5)	1.359 (2)	0.065 (3)	0.660 (15)
H34	0.3533	0.7647	1.4378	0.098*	0.660 (15)
C41	0.702 (3)	0.7808 (8)	0.792 (6)	0.0296 (18)	0.340 (15)
O41	0.736 (3)	0.7440 (11)	0.678 (7)	0.0463 (18)	0.340 (15)
O42	0.782 (4)	0.8158 (10)	0.816 (8)	0.0443 (15)	0.340 (15)
C42	0.567 (2)	0.7768 (6)	0.934 (3)	0.0375 (16)	0.340 (15)
H42A	0.4853	0.7819	0.8332	0.045*	0.340 (15)
H42B	0.5601	0.7447	0.9916	0.045*	0.340 (15)
C43	0.555 (2)	0.8103 (7)	1.134 (3)	0.056 (3)	0.340 (15)
H43A	0.5570	0.8425	1.0770	0.067*	0.340 (15)
H43B	0.6369	0.8060	1.2337	0.067*	0.340 (15)
C44	0.419 (3)	0.8032 (11)	1.272 (5)	0.0468 (19)	0.340 (15)
O43	0.304 (2)	0.8221 (6)	1.225 (5)	0.086 (3)	0.340 (15)
O44	0.425 (4)	0.7694 (12)	1.418 (5)	0.065 (3)	0.340 (15)
H44	0.3490	0.7681	1.4892	0.098*	0.340 (15)

### 4-(4-Methoxyphenyl)piperazin-1-ium hydrogensuccinate (VIII) Atomic displacement parameters (Å^2^)

**Table:**

	*U*^11^	*U*^22^	*U*^33^	*U*^12^	*U*^13^	*U*^23^
N1	0.0367 (12)	0.0305 (11)	0.0512 (17)	−0.0052 (10)	0.0144 (13)	−0.0049 (11)
C2	0.0389 (14)	0.0366 (13)	0.055 (2)	−0.0012 (11)	−0.0002 (16)	−0.0023 (14)
C3	0.0440 (15)	0.0361 (13)	0.0440 (18)	−0.0024 (11)	−0.0032 (15)	0.0033 (13)
N4	0.0388 (12)	0.0296 (10)	0.0357 (13)	−0.0015 (9)	0.0050 (11)	−0.0003 (10)
C5	0.0592 (17)	0.0331 (12)	0.0395 (17)	−0.0042 (12)	−0.0014 (16)	0.0044 (13)
C6	0.0606 (18)	0.0386 (14)	0.0446 (19)	−0.0070 (13)	0.0126 (18)	0.0056 (14)
C21	0.0345 (13)	0.0314 (12)	0.0333 (16)	0.0023 (10)	0.0055 (12)	−0.0008 (11)
C22	0.0421 (15)	0.0354 (13)	0.0406 (17)	0.0004 (11)	−0.0064 (14)	0.0037 (13)
C23	0.0483 (16)	0.0366 (14)	0.0472 (19)	−0.0025 (12)	−0.0064 (16)	0.0123 (13)
C24	0.0370 (13)	0.0332 (12)	0.052 (2)	−0.0019 (11)	−0.0022 (15)	0.0026 (13)
C25	0.0533 (17)	0.0456 (15)	0.046 (2)	−0.0059 (13)	−0.0156 (17)	0.0056 (15)
C26	0.0587 (18)	0.0414 (14)	0.0391 (19)	−0.0073 (13)	−0.0055 (16)	0.0104 (14)
O24	0.0604 (13)	0.0480 (11)	0.078 (2)	−0.0194 (10)	−0.0266 (15)	0.0143 (12)
C27	0.067 (2)	0.061 (2)	0.090 (3)	−0.0233 (17)	−0.036 (2)	0.011 (2)
C31	0.0307 (14)	0.020 (4)	0.0377 (18)	0.002 (3)	0.0121 (15)	0.002 (4)
O31	0.039 (4)	0.043 (4)	0.056 (4)	−0.018 (2)	0.025 (4)	−0.025 (2)
O32	0.0467 (11)	0.029 (4)	0.057 (5)	−0.011 (3)	0.027 (2)	−0.014 (3)
C32	0.043 (3)	0.025 (3)	0.045 (4)	0.002 (2)	0.019 (3)	0.004 (2)
C33	0.069 (5)	0.0348 (18)	0.064 (7)	0.008 (3)	0.043 (5)	0.009 (4)
C34	0.053 (3)	0.032 (2)	0.055 (5)	0.0033 (16)	0.031 (3)	−0.001 (2)
O33	0.097 (3)	0.054 (5)	0.107 (7)	0.039 (4)	0.067 (4)	0.016 (4)
O34	0.0654 (15)	0.0655 (15)	0.065 (8)	0.0228 (12)	0.054 (5)	0.026 (4)
C41	0.0307 (14)	0.020 (4)	0.0377 (18)	0.002 (3)	0.0121 (15)	0.002 (4)
O41	0.039 (4)	0.043 (4)	0.056 (4)	−0.018 (2)	0.025 (4)	−0.025 (2)
O42	0.0467 (11)	0.029 (4)	0.057 (5)	−0.011 (3)	0.027 (2)	−0.014 (3)
C42	0.043 (3)	0.025 (3)	0.045 (4)	0.002 (2)	0.019 (3)	0.004 (2)
C43	0.069 (5)	0.0348 (18)	0.064 (7)	0.008 (3)	0.043 (5)	0.009 (4)
C44	0.053 (3)	0.032 (2)	0.055 (5)	0.0033 (16)	0.031 (3)	−0.001 (2)
O43	0.097 (3)	0.054 (5)	0.107 (7)	0.039 (4)	0.067 (4)	0.016 (4)
O44	0.0654 (15)	0.0655 (15)	0.065 (8)	0.0228 (12)	0.054 (5)	0.026 (4)

### 4-(4-Methoxyphenyl)piperazin-1-ium hydrogensuccinate (VIII) Geometric parameters (Å, º)

**Table:**

N1—C6	1.484 (5)	O24—C27	1.419 (5)
N1—C2	1.484 (4)	C27—H27A	0.9600
N1—H11	0.86 (3)	C27—H27B	0.9600
N1—H12	0.98 (3)	C27—H27C	0.9600
C2—C3	1.512 (4)	C31—O32	1.247 (5)
C2—H2A	0.9700	C31—O31	1.270 (7)
C2—H2B	0.9700	C31—C32	1.513 (6)
C3—N4	1.459 (4)	C32—C33	1.517 (7)
C3—H3A	0.9700	C32—H32A	0.9700
C3—H3B	0.9700	C32—H32B	0.9700
N4—C21	1.429 (3)	C33—C34	1.505 (6)
N4—C5	1.469 (3)	C33—H33A	0.9700
C5—C6	1.508 (4)	C33—H33B	0.9700
C5—H5A	0.9700	C34—O33	1.233 (9)
C5—H5B	0.9700	C34—O34	1.280 (6)
C6—H6A	0.9700	O34—H34	0.8200
C6—H6B	0.9700	C41—O42	1.247 (9)
C21—C26	1.390 (4)	C41—O41	1.271 (10)
C21—C22	1.396 (4)	C41—C42	1.514 (9)
C22—C23	1.388 (4)	C42—C43	1.507 (11)
C22—H22	0.9300	C42—H42A	0.9700
C23—C24	1.378 (5)	C42—H42B	0.9700
C23—H23	0.9300	C43—C44	1.508 (10)
C24—C25	1.371 (4)	C43—H43A	0.9700
C24—O24	1.376 (3)	C43—H43B	0.9700
C25—C26	1.390 (4)	C44—O43	1.235 (13)
C25—H25	0.9300	C44—O44	1.282 (9)
C26—H26	0.9300	O44—H44	0.8200
			
C6—N1—C2	109.6 (2)	C21—C26—C25	122.0 (3)
C6—N1—H11	114 (3)	C21—C26—H26	119.0
C2—N1—H11	110 (2)	C25—C26—H26	119.0
C6—N1—H12	106 (2)	C24—O24—C27	117.0 (3)
C2—N1—H12	114 (2)	O24—C27—H27A	109.5
H11—N1—H12	103 (3)	O24—C27—H27B	109.5
N1—C2—C3	110.2 (2)	H27A—C27—H27B	109.5
N1—C2—H2A	109.6	O24—C27—H27C	109.5
C3—C2—H2A	109.6	H27A—C27—H27C	109.5
N1—C2—H2B	109.6	H27B—C27—H27C	109.5
C3—C2—H2B	109.6	O32—C31—O31	123.7 (8)
H2A—C2—H2B	108.1	O32—C31—C32	120.0 (6)
N4—C3—C2	112.4 (3)	O31—C31—C32	116.3 (6)
N4—C3—H3A	109.1	C31—C32—C33	114.8 (6)
C2—C3—H3A	109.1	C31—C32—H32A	108.6
N4—C3—H3B	109.1	C33—C32—H32A	108.6
C2—C3—H3B	109.1	C31—C32—H32B	108.6
H3A—C3—H3B	107.9	C33—C32—H32B	108.6
C21—N4—C3	115.3 (2)	H32A—C32—H32B	107.5
C21—N4—C5	114.3 (2)	C34—C33—C32	113.3 (6)
C3—N4—C5	110.7 (2)	C34—C33—H33A	108.9
N4—C5—C6	112.1 (2)	C32—C33—H33A	108.9
N4—C5—H5A	109.2	C34—C33—H33B	108.9
C6—C5—H5A	109.2	C32—C33—H33B	108.9
N4—C5—H5B	109.2	H33A—C33—H33B	107.7
C6—C5—H5B	109.2	O33—C34—O34	122.3 (7)
H5A—C5—H5B	107.9	O33—C34—C33	122.8 (7)
N1—C6—C5	109.8 (3)	O34—C34—C33	114.7 (6)
N1—C6—H6A	109.7	C34—O34—H34	109.5
C5—C6—H6A	109.7	O42—C41—O41	124.3 (15)
N1—C6—H6B	109.7	O42—C41—C42	119.7 (12)
C5—C6—H6B	109.7	O41—C41—C42	115.3 (12)
H6A—C6—H6B	108.2	C43—C42—C41	116.0 (12)
C26—C21—C22	116.7 (2)	C43—C42—H42A	108.3
C26—C21—N4	120.9 (3)	C41—C42—H42A	108.3
C22—C21—N4	122.3 (3)	C43—C42—H42B	108.3
C23—C22—C21	121.2 (3)	C41—C42—H42B	108.3
C23—C22—H22	119.4	H42A—C42—H42B	107.4
C21—C22—H22	119.4	C42—C43—C44	113.1 (15)
C24—C23—C22	120.8 (3)	C42—C43—H43A	109.0
C24—C23—H23	119.6	C44—C43—H43A	109.0
C22—C23—H23	119.6	C42—C43—H43B	109.0
C25—C24—O24	124.8 (3)	C44—C43—H43B	109.0
C25—C24—C23	119.1 (3)	H43A—C43—H43B	107.8
O24—C24—C23	116.0 (3)	O43—C44—O44	120.3 (16)
C24—C25—C26	120.2 (3)	O43—C44—C43	123.7 (14)
C24—C25—H25	119.9	O44—C44—C43	114.8 (12)
C26—C25—H25	119.9	C44—O44—H44	109.5
			
C6—N1—C2—C3	−57.9 (3)	O24—C24—C25—C26	179.3 (3)
N1—C2—C3—N4	56.2 (3)	C23—C24—C25—C26	−1.5 (5)
C2—C3—N4—C21	174.2 (2)	C22—C21—C26—C25	1.0 (4)
C2—C3—N4—C5	−54.0 (3)	N4—C21—C26—C25	179.3 (3)
C21—N4—C5—C6	−172.9 (3)	C24—C25—C26—C21	0.4 (5)
C3—N4—C5—C6	54.8 (3)	C25—C24—O24—C27	−3.1 (5)
C2—N1—C6—C5	58.5 (3)	C23—C24—O24—C27	177.7 (3)
N4—C5—C6—N1	−57.4 (3)	O32—C31—C32—C33	32 (2)
C3—N4—C21—C26	161.9 (3)	O31—C31—C32—C33	−146 (2)
C5—N4—C21—C26	31.8 (4)	C31—C32—C33—C34	−178.3 (12)
C3—N4—C21—C22	−19.9 (4)	C32—C33—C34—O33	−162 (2)
C5—N4—C21—C22	−150.0 (3)	C32—C33—C34—O34	23 (2)
C26—C21—C22—C23	−1.3 (4)	O42—C41—C42—C43	−13 (5)
N4—C21—C22—C23	−179.6 (3)	O41—C41—C42—C43	157 (4)
C21—C22—C23—C24	0.2 (5)	C41—C42—C43—C44	−178 (2)
C22—C23—C24—C25	1.2 (5)	C42—C43—C44—O43	−86 (4)
C22—C23—C24—O24	−179.5 (3)	C42—C43—C44—O44	82 (4)

### 4-(4-Methoxyphenyl)piperazin-1-ium hydrogensuccinate (VIII) Hydrogen-bond geometry (Å, º)

**Table:**

*D*—H···*A*	*D*—H	H···*A*	*D*···*A*	*D*—H···*A*
N1—H11···O31	0.86 (3)	1.90 (3)	2.750 (15)	167 (4)
N1—H12···O32^i^	0.98 (3)	1.77 (4)	2.741 (19)	171 (3)
O34—H34···O31^ii^	0.82	1.79	2.60 (2)	168
N1—H11···O41	0.86 (3)	2.18 (4)	3.03 (3)	165 (4)
N1—H12···O42^i^	0.98 (3)	1.82 (5)	2.77 (4)	163 (3)
O44—H44···O41^ii^	0.82	1.56	2.35 (2)	161
C3—H3*A*···*Cg*2^iii^	0.97	2.76	3.652 (3)	154

Symmetry codes: (i) *x*−1/2, −*y*+3/2, *z*; (ii) *x*−1/2, −*y*+3/2, *z*+1; (iii) −*x*+1, −*y*+1, *z*+1/2.

### 4-(4-Methoxyphenyl)piperazin-1-ium hydrogenfumarate (IX) Crystal data

**Table:**

C_11_H_17_N_2_O^+^·C_4_H_3_O_4_^−^	*D*_x_ = 1.356 Mg m^−^^3^
*M**_r_* = 308.33	Mo *K*α radiation, λ = 0.71073 Å
Orthorhombic, *P**n**a*2_1_	Cell parameters from 2829 reflections
*a* = 9.069 (1) Å	θ = 2.7–27.7°
*b* = 28.528 (3) Å	µ = 0.10 mm^−^^1^
*c* = 5.8375 (9) Å	*T* = 296 K
*V* = 1510.3 (3) Å^3^	Plate, colourless
*Z* = 4	0.48 × 0.48 × 0.08 mm
*F*(000) = 656	

### 4-(4-Methoxyphenyl)piperazin-1-ium hydrogenfumarate (IX) Data collection

**Table:**

Oxford Diffraction Xcalibur with Sapphire CCD diffractometer	2827 independent reflections
Radiation source: Enhance (Mo) X-ray Source	2316 reflections with *I* > 2σ(*I*)
Graphite monochromator	*R*_int_ = 0.015
ω scans	θ_max_ = 27.5°, θ_min_ = 2.7°
Absorption correction: multi-scan (CrysAlis RED; Oxford Diffraction, 2009)	*h* = −8→11
*T*_min_ = 0.888, *T*_max_ = 0.992	*k* = −32→35
5834 measured reflections	*l* = −7→6

### 4-(4-Methoxyphenyl)piperazin-1-ium hydrogenfumarate (IX) Refinement

**Table:**

Refinement on *F*^2^	Primary atom site location: difference Fourier map
Least-squares matrix: full	Hydrogen site location: mixed
*R*[*F*^2^ > 2σ(*F*^2^)] = 0.041	H atoms treated by a mixture of independent and constrained refinement
*wR*(*F*^2^) = 0.101	*w* = 1/[σ^2^(*F*_o_^2^) + (0.0429*P*)^2^ + 0.4265*P*] where *P* = (*F*_o_^2^ + 2*F*_c_^2^)/3
*S* = 1.05	(Δ/σ)_max_ < 0.001
2827 reflections	Δρ_max_ = 0.15 e Å^−^^3^
221 parameters	Δρ_min_ = −0.14 e Å^−^^3^
11 restraints	Absolute structure: Flack *x* determined using 769 quotients [(*I*^+^)-(*I*^-^)]/[(*I*^+^)+(*I*^-^)] (Parsons *et al.*, 2013)

### 4-(4-Methoxyphenyl)piperazin-1-ium hydrogenfumarate (IX) Special details

**Table:**

Experimental. Compound (IX). IR (KBr , cm^-1^) 3001 (NH_2_), 2839 (OCH_3_), 1562 (COO). NMR (CDCl_3_) δ(^1^H) ) 3.09 (m, 4H, piperazine), 3.35 (m, 4H, piperazine), 3.77 (s, 3H, OCH_3_), 6.26 (s, 2H, fumarate), 6.90 (m, 4H, methoxyphenyl).
Geometry. All esds (except the esd in the dihedral angle between two l.s. planes) are estimated using the full covariance matrix. The cell esds are taken into account individually in the estimation of esds in distances, angles and torsion angles; correlations between esds in cell parameters are only used when they are defined by crystal symmetry. An approximate (isotropic) treatment of cell esds is used for estimating esds involving l.s. planes.

### 4-(4-Methoxyphenyl)piperazin-1-ium hydrogenfumarate (IX) Fractional atomic coordinates and isotropic or equivalent isotropic displacement parameters (Å^2^)

**Table:**

	*x*	*y*	*z*	*U*_iso_*/*U*_eq_	Occ. (<1)
N1	0.5253 (3)	0.66688 (8)	0.5154 (5)	0.0448 (6)	
H11	0.588 (4)	0.6872 (12)	0.518 (6)	0.054*	
H12	0.440 (4)	0.6776 (11)	0.596 (6)	0.054*	
C2	0.5885 (3)	0.62425 (10)	0.6244 (6)	0.0490 (8)	
H2A	0.6182	0.6315	0.7801	0.059*	
H2B	0.6753	0.6144	0.5403	0.059*	
C3	0.4771 (3)	0.58500 (9)	0.6274 (6)	0.0449 (7)	
H3A	0.5225	0.5572	0.6916	0.054*	
H3B	0.3952	0.5937	0.7254	0.054*	
N4	0.4215 (3)	0.57432 (7)	0.3988 (4)	0.0395 (6)	
C5	0.3637 (4)	0.61653 (10)	0.2869 (6)	0.0505 (8)	
H5A	0.2767	0.6272	0.3685	0.061*	
H5B	0.3344	0.6089	0.1315	0.061*	
C6	0.4751 (4)	0.65530 (10)	0.2808 (6)	0.0540 (9)	
H6A	0.5589	0.6458	0.1886	0.065*	
H6B	0.4317	0.6829	0.2106	0.065*	
C21	0.3300 (3)	0.53374 (9)	0.3824 (5)	0.0372 (6)	
C22	0.3313 (3)	0.49854 (9)	0.5482 (6)	0.0441 (7)	
H22	0.3919	0.5017	0.6758	0.053*	
C23	0.2439 (3)	0.45907 (10)	0.5261 (7)	0.0485 (8)	
H23	0.2468	0.4361	0.6393	0.058*	
C24	0.1529 (3)	0.45301 (10)	0.3401 (6)	0.0456 (8)	
C25	0.1521 (4)	0.48719 (10)	0.1732 (6)	0.0518 (8)	
H25	0.0924	0.4836	0.0448	0.062*	
C26	0.2391 (4)	0.52673 (11)	0.1945 (6)	0.0509 (8)	
H26	0.2366	0.5493	0.0796	0.061*	
O24	0.0698 (3)	0.41291 (8)	0.3349 (5)	0.0668 (8)	
C27	−0.0259 (4)	0.40672 (14)	0.1473 (9)	0.0804 (13)	
H27A	−0.0819	0.3785	0.1680	0.121*	
H27B	0.0307	0.4045	0.0088	0.121*	
H27C	−0.0918	0.4330	0.1373	0.121*	
C31	0.7171 (3)	0.77494 (10)	0.8023 (6)	0.0452 (7)	
O31	0.7323 (3)	0.73835 (7)	0.6832 (5)	0.0673 (8)	
O32	0.7875 (3)	0.81180 (7)	0.7776 (5)	0.0595 (7)	
C32	0.6095 (4)	0.77182 (10)	0.9941 (6)	0.0526 (9)	0.906 (9)
H32	0.5993	0.7427	1.0643	0.063*	0.906 (9)
C33	0.5305 (7)	0.80489 (15)	1.0714 (12)	0.0607 (11)	0.906 (9)
H33	0.5456	0.8345	1.0089	0.073*	0.906 (9)
C34	0.4163 (5)	0.80084 (15)	1.2518 (8)	0.0518 (9)	0.906 (9)
O33	0.3241 (5)	0.83097 (16)	1.2845 (8)	0.0992 (18)	0.906 (9)
O34	0.4218 (4)	0.76290 (13)	1.3671 (8)	0.0734 (13)	0.906 (9)
H34	0.3526	0.7621	1.4574	0.110*	0.906 (9)
C42	0.6095 (4)	0.77182 (10)	0.9941 (6)	0.0526 (9)	0.094 (9)
H42	0.5982	0.7421	1.0566	0.063*	0.094 (9)
C43	0.531 (6)	0.8035 (9)	1.086 (10)	0.0607 (11)	0.094 (9)
H43	0.5533	0.8343	1.0477	0.073*	0.094 (9)
C44	0.407 (4)	0.7961 (12)	1.247 (6)	0.0518 (9)	0.094 (9)
O43	0.280 (4)	0.8058 (15)	1.203 (7)	0.0992 (18)	0.094 (9)
O44	0.444 (4)	0.7801 (13)	1.442 (6)	0.0734 (13)	0.094 (9)
H44	0.3697	0.7743	1.5169	0.110*	0.094 (9)

### 4-(4-Methoxyphenyl)piperazin-1-ium hydrogenfumarate (IX) Atomic displacement parameters (Å^2^)

**Table:**

	*U*^11^	*U*^22^	*U*^33^	*U*^12^	*U*^13^	*U*^23^
N1	0.0436 (14)	0.0378 (12)	0.0531 (18)	−0.0049 (10)	0.0167 (13)	−0.0020 (12)
C2	0.0444 (16)	0.0431 (15)	0.060 (2)	0.0018 (12)	0.0054 (16)	−0.0042 (15)
C3	0.0482 (17)	0.0398 (14)	0.047 (2)	−0.0019 (13)	−0.0013 (15)	0.0052 (13)
N4	0.0466 (13)	0.0348 (11)	0.0371 (14)	0.0016 (10)	0.0048 (12)	−0.0005 (10)
C5	0.072 (2)	0.0403 (14)	0.0396 (19)	−0.0043 (14)	−0.0015 (17)	0.0079 (14)
C6	0.077 (2)	0.0423 (14)	0.043 (2)	−0.0044 (15)	0.0172 (18)	0.0048 (15)
C21	0.0398 (15)	0.0355 (13)	0.0363 (17)	0.0060 (11)	0.0060 (14)	0.0009 (12)
C22	0.0484 (17)	0.0402 (14)	0.0437 (19)	0.0029 (12)	−0.0071 (15)	0.0040 (14)
C23	0.0532 (18)	0.0413 (15)	0.0510 (19)	0.0005 (13)	−0.0054 (17)	0.0123 (15)
C24	0.0391 (15)	0.0409 (14)	0.057 (2)	0.0015 (12)	−0.0036 (15)	0.0009 (14)
C25	0.0560 (19)	0.0519 (17)	0.048 (2)	−0.0013 (15)	−0.0160 (17)	0.0035 (16)
C26	0.065 (2)	0.0455 (15)	0.042 (2)	−0.0041 (14)	−0.0061 (16)	0.0115 (14)
O24	0.0633 (15)	0.0532 (12)	0.084 (2)	−0.0163 (11)	−0.0224 (15)	0.0125 (13)
C27	0.064 (2)	0.070 (2)	0.107 (4)	−0.0205 (19)	−0.034 (3)	0.012 (2)
C31	0.0431 (16)	0.0435 (15)	0.049 (2)	0.0003 (12)	0.0227 (15)	−0.0016 (14)
O31	0.0655 (15)	0.0577 (13)	0.079 (2)	−0.0153 (11)	0.0428 (14)	−0.0234 (13)
O32	0.0632 (14)	0.0469 (11)	0.0684 (17)	−0.0123 (10)	0.0369 (13)	−0.0050 (11)
C32	0.0564 (19)	0.0380 (14)	0.063 (2)	−0.0018 (14)	0.0321 (18)	−0.0001 (15)
C33	0.067 (2)	0.0474 (16)	0.067 (3)	0.0048 (16)	0.034 (2)	0.0065 (17)
C34	0.0558 (19)	0.0416 (18)	0.058 (2)	0.0052 (15)	0.0280 (18)	−0.0037 (15)
O33	0.115 (3)	0.064 (2)	0.119 (3)	0.045 (2)	0.081 (3)	0.028 (2)
O34	0.081 (2)	0.0502 (19)	0.089 (3)	0.0215 (17)	0.061 (2)	0.0185 (17)
C42	0.0564 (19)	0.0380 (14)	0.063 (2)	−0.0018 (14)	0.0321 (18)	−0.0001 (15)
C43	0.067 (2)	0.0474 (16)	0.067 (3)	0.0048 (16)	0.034 (2)	0.0065 (17)
C44	0.0558 (19)	0.0416 (18)	0.058 (2)	0.0052 (15)	0.0280 (18)	−0.0037 (15)
O43	0.115 (3)	0.064 (2)	0.119 (3)	0.045 (2)	0.081 (3)	0.028 (2)
O44	0.081 (2)	0.0502 (19)	0.089 (3)	0.0215 (17)	0.061 (2)	0.0185 (17)

### 4-(4-Methoxyphenyl)piperazin-1-ium hydrogenfumarate (IX) Geometric parameters (Å, º)

**Table:**

N1—C6	1.481 (4)	C24—C25	1.378 (4)
N1—C2	1.487 (4)	C25—C26	1.382 (4)
N1—H11	0.81 (3)	C25—H25	0.9300
N1—H12	0.96 (4)	C26—H26	0.9300
C2—C3	1.508 (4)	O24—C27	1.409 (5)
C2—H2A	0.9700	C27—H27A	0.9600
C2—H2B	0.9700	C27—H27B	0.9600
C3—N4	1.459 (4)	C27—H27C	0.9600
C3—H3A	0.9700	C31—O32	1.239 (3)
C3—H3B	0.9700	C31—O31	1.262 (4)
N4—C21	1.428 (3)	C31—C32	1.488 (4)
N4—C5	1.467 (4)	C32—C33	1.268 (4)
C5—C6	1.499 (4)	C32—H32	0.9300
C5—H5A	0.9700	C33—C34	1.481 (5)
C5—H5B	0.9700	C33—H33	0.9300
C6—H6A	0.9700	C34—O33	1.214 (4)
C6—H6B	0.9700	C34—O34	1.276 (5)
C21—C26	1.387 (4)	O34—H34	0.8200
C21—C22	1.395 (4)	C43—C44	1.479 (12)
C22—C23	1.383 (4)	C43—H43	0.9300
C22—H22	0.9300	C44—O43	1.212 (12)
C23—C24	1.375 (5)	C44—O44	1.274 (12)
C23—H23	0.9300	O44—H44	0.8200
C24—O24	1.370 (3)		
			
C6—N1—C2	109.4 (2)	C21—C22—H22	119.4
C6—N1—H11	113 (3)	C24—C23—C22	121.3 (3)
C2—N1—H11	108 (2)	C24—C23—H23	119.3
C6—N1—H12	106 (2)	C22—C23—H23	119.3
C2—N1—H12	111 (2)	O24—C24—C23	116.9 (3)
H11—N1—H12	109 (3)	O24—C24—C25	124.9 (3)
N1—C2—C3	110.7 (3)	C23—C24—C25	118.2 (3)
N1—C2—H2A	109.5	C24—C25—C26	120.7 (3)
C3—C2—H2A	109.5	C24—C25—H25	119.7
N1—C2—H2B	109.5	C26—C25—H25	119.7
C3—C2—H2B	109.5	C25—C26—C21	121.9 (3)
H2A—C2—H2B	108.1	C25—C26—H26	119.0
N4—C3—C2	112.1 (3)	C21—C26—H26	119.0
N4—C3—H3A	109.2	C24—O24—C27	117.4 (3)
C2—C3—H3A	109.2	O24—C27—H27A	109.5
N4—C3—H3B	109.2	O24—C27—H27B	109.5
C2—C3—H3B	109.2	H27A—C27—H27B	109.5
H3A—C3—H3B	107.9	O24—C27—H27C	109.5
C21—N4—C3	115.6 (2)	H27A—C27—H27C	109.5
C21—N4—C5	115.3 (2)	H27B—C27—H27C	109.5
C3—N4—C5	111.1 (2)	O32—C31—O31	125.6 (3)
N4—C5—C6	112.0 (3)	O32—C31—C32	118.5 (3)
N4—C5—H5A	109.2	O31—C31—C32	115.9 (2)
C6—C5—H5A	109.2	C33—C32—C31	126.4 (3)
N4—C5—H5B	109.2	C33—C32—H32	116.8
C6—C5—H5B	109.2	C31—C32—H32	116.8
H5A—C5—H5B	107.9	C32—C33—C34	126.2 (4)
N1—C6—C5	110.5 (3)	C32—C33—H33	116.9
N1—C6—H6A	109.6	C34—C33—H33	116.9
C5—C6—H6A	109.6	O33—C34—O34	123.0 (4)
N1—C6—H6B	109.6	O33—C34—C33	122.5 (4)
C5—C6—H6B	109.6	O34—C34—C33	114.5 (3)
H6A—C6—H6B	108.1	C34—O34—H34	109.5
C26—C21—C22	116.7 (3)	C44—C43—H43	116.8
C26—C21—N4	121.0 (3)	O43—C44—O44	121.3 (18)
C22—C21—N4	122.2 (3)	O43—C44—C43	124 (2)
C23—C22—C21	121.1 (3)	O44—C44—C43	114.9 (18)
C23—C22—H22	119.4	C44—O44—H44	109.5
			
C6—N1—C2—C3	−57.2 (3)	C22—C23—C24—O24	−179.4 (3)
N1—C2—C3—N4	55.8 (3)	C22—C23—C24—C25	0.8 (5)
C2—C3—N4—C21	172.3 (2)	O24—C24—C25—C26	179.4 (3)
C2—C3—N4—C5	−53.8 (3)	C23—C24—C25—C26	−0.9 (5)
C21—N4—C5—C6	−171.4 (3)	C24—C25—C26—C21	0.0 (5)
C3—N4—C5—C6	54.7 (3)	C22—C21—C26—C25	0.9 (5)
C2—N1—C6—C5	57.9 (3)	N4—C21—C26—C25	178.6 (3)
N4—C5—C6—N1	−57.2 (4)	C23—C24—O24—C27	178.8 (3)
C3—N4—C21—C26	162.4 (3)	C25—C24—O24—C27	−1.4 (5)
C5—N4—C21—C26	30.5 (4)	O32—C31—C32—C33	34.7 (7)
C3—N4—C21—C22	−20.1 (4)	O31—C31—C32—C33	−148.0 (6)
C5—N4—C21—C22	−151.9 (3)	C31—C32—C33—C34	175.5 (5)
C26—C21—C22—C23	−0.9 (4)	C32—C33—C34—O33	−164.1 (8)
N4—C21—C22—C23	−178.6 (3)	C32—C33—C34—O34	14.9 (10)
C21—C22—C23—C24	0.1 (5)		

### 4-(4-Methoxyphenyl)piperazin-1-ium hydrogenfumarate (IX) Hydrogen-bond geometry (Å, º)

**Table:**

*D*—H···*A*	*D*—H	H···*A*	*D*···*A*	*D*—H···*A*
N1—H11···O31	0.81 (4)	2.18 (3)	2.940 (4)	155 (3)
N1—H12···O32^i^	0.96 (4)	1.77 (4)	2.714 (4)	169 (3)
O34—H34···O31^ii^	0.82	1.71	2.522 (5)	170
O44—H44···O31^ii^	0.82	1.62	2.44 (2)	175
C3—H3*A*···*Cg*2^iii^	0.97	2.76	3.650 (3)	153

Symmetry codes: (i) *x*−1/2, −*y*+3/2, *z*; (ii) *x*−1/2, −*y*+3/2, *z*+1; (iii) −*x*+1, −*y*+1, *z*+1/2.

### 4-(4-Methoxyphenyl)piperazin-1-ium hydrogenmaleate (X) Crystal data

**Table:**

C_11_H_17_N_2_O^+^·C_4_H_3_O_4_^−^	*F*(000) = 656
*M**_r_* = 308.33	*D*_x_ = 1.335 Mg m^−^^3^
Monoclinic, *P*2_1_/*c*	Mo *K*α radiation, λ = 0.71073 Å
*a* = 9.063 (1) Å	Cell parameters from 3315 reflections
*b* = 6.4956 (9) Å	θ = 2.7–27.8°
*c* = 26.093 (3) Å	µ = 0.10 mm^−^^1^
β = 93.18 (1)°	*T* = 296 K
*V* = 1533.7 (3) Å^3^	Block, colourless
*Z* = 4	0.48 × 0.44 × 0.32 mm

### 4-(4-Methoxyphenyl)piperazin-1-ium hydrogenmaleate (X) Data collection

**Table:**

Oxford Diffraction Xcalibur with Sapphire CCD diffractometer	3311 independent reflections
Radiation source: Enhance (Mo) X-ray Source	2459 reflections with *I* > 2σ(*I*)
Graphite monochromator	*R*_int_ = 0.014
ω scans	θ_max_ = 27.6°, θ_min_ = 2.7°
Absorption correction: multi-scan (CrysAlis RED; Oxford Diffraction, 2009)	*h* = −11→11
*T*_min_ = 0.871, *T*_max_ = 0.968	*k* = −6→8
6112 measured reflections	*l* = −26→33

### 4-(4-Methoxyphenyl)piperazin-1-ium hydrogenmaleate (X) Refinement

**Table:**

Refinement on *F*^2^	Hydrogen site location: mixed
Least-squares matrix: full	H atoms treated by a mixture of independent and constrained refinement
*R*[*F*^2^ > 2σ(*F*^2^)] = 0.040	*w* = 1/[σ^2^(*F*_o_^2^) + (0.0521*P*)^2^ + 0.3046*P*] where *P* = (*F*_o_^2^ + 2*F*_c_^2^)/3
*wR*(*F*^2^) = 0.111	(Δ/σ)_max_ = 0.001
*S* = 1.05	Δρ_max_ = 0.21 e Å^−^^3^
3311 reflections	Δρ_min_ = −0.13 e Å^−^^3^
210 parameters	Extinction correction: SHELXL, Fc^*^=kFc[1+0.001xFc^2^λ^3^/sin(2θ)]^-1/4^
0 restraints	Extinction coefficient: 0.0192 (18)
Primary atom site location: difference Fourier map	

### 4-(4-Methoxyphenyl)piperazin-1-ium hydrogenmaleate (X) Special details

**Table:**

Experimental. Compound (X). IR (KBr , cm^-1^) 3073 (NH_2_), 2836 (OCH_3_), 1565 (COO). NMR (CDCl_3_) δ(^1^H) ) 3.34 (m, 4H, piperazine), 3.41 (m, 4H, piperazine), 3.77 (s, 3H, OCH_3_), 6.29 (s, 2H, maleate), 6.90 (m, 4H, methoxyphenyl).
Geometry. All esds (except the esd in the dihedral angle between two l.s. planes) are estimated using the full covariance matrix. The cell esds are taken into account individually in the estimation of esds in distances, angles and torsion angles; correlations between esds in cell parameters are only used when they are defined by crystal symmetry. An approximate (isotropic) treatment of cell esds is used for estimating esds involving l.s. planes.

### 4-(4-Methoxyphenyl)piperazin-1-ium hydrogenmaleate (X) Fractional atomic coordinates and isotropic or equivalent isotropic displacement parameters (Å^2^)

**Table:**

	*x*	*y*	*z*	*U*_iso_*/*U*_eq_	
N1	0.54059 (15)	0.1806 (2)	0.56144 (5)	0.0431 (3)	
H11	0.6147 (19)	0.166 (3)	0.5393 (6)	0.052*	
H12	0.452 (2)	0.174 (3)	0.5426 (7)	0.052*	
C2	0.5537 (2)	0.3928 (3)	0.58165 (6)	0.0531 (4)	
H2A	0.5428	0.4903	0.5535	0.064*	
H2B	0.6506	0.4121	0.5986	0.064*	
C3	0.43600 (18)	0.4321 (2)	0.61930 (6)	0.0468 (4)	
H3A	0.4472	0.5706	0.6329	0.056*	
H3B	0.3392	0.4222	0.6016	0.056*	
N4	0.44597 (13)	0.28484 (17)	0.66144 (4)	0.0362 (3)	
C5	0.43395 (19)	0.0748 (2)	0.64123 (6)	0.0481 (4)	
H5A	0.3370	0.0559	0.6243	0.058*	
H5B	0.4438	−0.0222	0.6695	0.058*	
C6	0.55084 (18)	0.0302 (2)	0.60367 (6)	0.0468 (4)	
H6A	0.6480	0.0377	0.6211	0.056*	
H6B	0.5373	−0.1077	0.5900	0.056*	
C21	0.35362 (15)	0.3268 (2)	0.70263 (5)	0.0354 (3)	
C22	0.26577 (18)	0.5006 (2)	0.70426 (6)	0.0481 (4)	
H22	0.2632	0.5920	0.6768	0.058*	
C23	0.18120 (18)	0.5421 (3)	0.74593 (6)	0.0530 (4)	
H23	0.1247	0.6616	0.7463	0.064*	
C24	0.18071 (17)	0.4076 (3)	0.78649 (5)	0.0489 (4)	
C25	0.2673 (2)	0.2332 (3)	0.78540 (6)	0.0537 (4)	
H25	0.2679	0.1410	0.8127	0.064*	
C26	0.35276 (18)	0.1936 (3)	0.74451 (6)	0.0464 (4)	
H326	0.4110	0.0757	0.7448	0.056*	
O24	0.10103 (14)	0.4323 (2)	0.82944 (4)	0.0706 (4)	
C27	0.0207 (2)	0.6185 (4)	0.83401 (7)	0.0844 (7)	
H27A	−0.0231	0.6219	0.8667	0.127*	
H27B	−0.0556	0.6260	0.8070	0.127*	
H27C	0.0864	0.7334	0.8314	0.127*	
C31	0.81864 (16)	0.2456 (2)	0.48041 (7)	0.0439 (4)	
C32	0.93279 (17)	0.2644 (2)	0.44164 (6)	0.0460 (4)	
H32	0.8951	0.2815	0.4080	0.055*	
C33	1.07926 (16)	0.2605 (2)	0.44719 (6)	0.0429 (4)	
H33	1.1274	0.2743	0.4168	0.052*	
C34	1.17846 (15)	0.2380 (2)	0.49393 (6)	0.0370 (3)	
O31	0.68826 (12)	0.23449 (19)	0.46522 (5)	0.0627 (4)	
O32	0.85878 (12)	0.23939 (18)	0.52832 (4)	0.0520 (3)	
O33	1.12495 (12)	0.23595 (18)	0.53839 (4)	0.0488 (3)	
H33A	0.996 (2)	0.240 (3)	0.5354 (8)	0.073*	
O34	1.31216 (11)	0.22095 (17)	0.48789 (4)	0.0495 (3)	

### 4-(4-Methoxyphenyl)piperazin-1-ium hydrogenmaleate (X) Atomic displacement parameters (Å^2^)

**Table:**

	*U*^11^	*U*^22^	*U*^33^	*U*^12^	*U*^13^	*U*^23^
N1	0.0357 (6)	0.0568 (8)	0.0375 (7)	0.0007 (6)	0.0084 (5)	−0.0023 (6)
C2	0.0645 (10)	0.0474 (9)	0.0493 (9)	−0.0055 (8)	0.0214 (8)	0.0037 (7)
C3	0.0590 (10)	0.0388 (8)	0.0440 (8)	0.0018 (7)	0.0146 (7)	0.0034 (7)
N4	0.0396 (6)	0.0349 (6)	0.0346 (6)	0.0002 (5)	0.0054 (5)	0.0009 (5)
C5	0.0591 (10)	0.0385 (8)	0.0484 (9)	−0.0012 (7)	0.0186 (7)	0.0000 (7)
C6	0.0507 (9)	0.0450 (9)	0.0454 (8)	0.0080 (7)	0.0087 (7)	−0.0013 (7)
C21	0.0340 (7)	0.0391 (7)	0.0329 (7)	−0.0023 (6)	0.0007 (5)	−0.0016 (6)
C22	0.0550 (9)	0.0482 (9)	0.0419 (8)	0.0101 (7)	0.0106 (7)	0.0080 (7)
C23	0.0539 (10)	0.0584 (10)	0.0472 (9)	0.0176 (8)	0.0085 (7)	0.0009 (8)
C24	0.0435 (8)	0.0715 (11)	0.0316 (7)	0.0060 (8)	0.0023 (6)	−0.0037 (7)
C25	0.0645 (10)	0.0662 (11)	0.0305 (7)	0.0109 (9)	0.0042 (7)	0.0111 (7)
C26	0.0525 (9)	0.0508 (9)	0.0358 (8)	0.0120 (7)	0.0001 (6)	0.0044 (6)
O24	0.0722 (8)	0.1024 (11)	0.0386 (6)	0.0251 (7)	0.0175 (6)	0.0033 (6)
C27	0.0759 (13)	0.126 (2)	0.0528 (11)	0.0418 (14)	0.0177 (10)	−0.0042 (12)
C31	0.0357 (8)	0.0337 (8)	0.0631 (10)	0.0035 (6)	0.0104 (7)	0.0045 (7)
C32	0.0425 (8)	0.0529 (9)	0.0427 (8)	0.0026 (7)	0.0035 (6)	0.0069 (7)
C33	0.0411 (8)	0.0493 (9)	0.0395 (8)	0.0003 (7)	0.0118 (6)	0.0033 (7)
C34	0.0352 (7)	0.0300 (7)	0.0463 (8)	−0.0020 (6)	0.0071 (6)	−0.0005 (6)
O31	0.0334 (6)	0.0690 (8)	0.0858 (9)	0.0036 (5)	0.0040 (6)	0.0110 (7)
O32	0.0418 (6)	0.0641 (8)	0.0517 (7)	0.0009 (5)	0.0172 (5)	0.0003 (5)
O33	0.0427 (6)	0.0647 (8)	0.0393 (6)	0.0007 (5)	0.0056 (4)	−0.0020 (5)
O34	0.0337 (5)	0.0572 (7)	0.0580 (7)	−0.0010 (5)	0.0069 (5)	0.0012 (5)

### 4-(4-Methoxyphenyl)piperazin-1-ium hydrogenmaleate (X) Geometric parameters (Å, º)

**Table:**

N1—C6	1.472 (2)	C23—H23	0.9300
N1—C2	1.478 (2)	C24—O24	1.3759 (17)
N1—H11	0.915 (17)	C24—C25	1.379 (2)
N1—H12	0.917 (18)	C25—C26	1.377 (2)
C2—C3	1.511 (2)	C25—H25	0.9300
C2—H2A	0.9700	C26—H326	0.9300
C2—H2B	0.9700	O24—C27	1.420 (3)
C3—N4	1.4567 (18)	C27—H27A	0.9600
C3—H3A	0.9700	C27—H27B	0.9600
C3—H3B	0.9700	C27—H27C	0.9600
N4—C21	1.4248 (17)	C31—O31	1.2274 (19)
N4—C5	1.4645 (19)	C31—O32	1.283 (2)
C5—C6	1.511 (2)	C31—C32	1.492 (2)
C5—H5A	0.9700	C32—C33	1.328 (2)
C5—H5B	0.9700	C32—H32	0.9300
C6—H6A	0.9700	C33—C34	1.482 (2)
C6—H6B	0.9700	C33—H33	0.9300
C21—C22	1.383 (2)	C34—O34	1.2355 (17)
C21—C26	1.394 (2)	C34—O33	1.2820 (17)
C22—C23	1.391 (2)	O32—H33A	1.25 (2)
C22—H22	0.9300	O33—H33A	1.17 (2)
C23—C24	1.373 (2)		
			
C6—N1—C2	110.57 (12)	C21—C22—C23	121.70 (14)
C6—N1—H11	112.8 (11)	C21—C22—H22	119.2
C2—N1—H11	105.8 (11)	C23—C22—H22	119.2
C6—N1—H12	112.6 (11)	C24—C23—C22	120.31 (15)
C2—N1—H12	106.8 (11)	C24—C23—H23	119.8
H11—N1—H12	107.8 (15)	C22—C23—H23	119.8
N1—C2—C3	110.13 (13)	C23—C24—O24	125.33 (15)
N1—C2—H2A	109.6	C23—C24—C25	118.74 (14)
C3—C2—H2A	109.6	O24—C24—C25	115.93 (14)
N1—C2—H2B	109.6	C26—C25—C24	120.98 (15)
C3—C2—H2B	109.6	C26—C25—H25	119.5
H2A—C2—H2B	108.1	C24—C25—H25	119.5
N4—C3—C2	111.29 (13)	C25—C26—C21	121.25 (15)
N4—C3—H3A	109.4	C25—C26—H326	119.4
C2—C3—H3A	109.4	C21—C26—H326	119.4
N4—C3—H3B	109.4	C24—O24—C27	117.53 (15)
C2—C3—H3B	109.4	O24—C27—H27A	109.5
H3A—C3—H3B	108.0	O24—C27—H27B	109.5
C21—N4—C3	115.42 (11)	H27A—C27—H27B	109.5
C21—N4—C5	114.50 (11)	O24—C27—H27C	109.5
C3—N4—C5	109.87 (12)	H27A—C27—H27C	109.5
N4—C5—C6	111.79 (12)	H27B—C27—H27C	109.5
N4—C5—H5A	109.3	O31—C31—O32	121.90 (14)
C6—C5—H5A	109.3	O31—C31—C32	118.52 (15)
N4—C5—H5B	109.3	O32—C31—C32	119.57 (14)
C6—C5—H5B	109.3	C33—C32—C31	130.65 (15)
H5A—C5—H5B	107.9	C33—C32—H32	114.7
N1—C6—C5	109.95 (13)	C31—C32—H32	114.7
N1—C6—H6A	109.7	C32—C33—C34	130.48 (13)
C5—C6—H6A	109.7	C32—C33—H33	114.8
N1—C6—H6B	109.7	C34—C33—H33	114.8
C5—C6—H6B	109.7	O34—C34—O33	122.51 (14)
H6A—C6—H6B	108.2	O34—C34—C33	117.29 (13)
C22—C21—C26	117.01 (13)	O33—C34—C33	120.20 (12)
C22—C21—N4	122.86 (13)	C31—O32—H33A	111.7 (9)
C26—C21—N4	120.09 (13)	C34—O33—H33A	111.5 (10)
			
C6—N1—C2—C3	−57.07 (18)	C22—C23—C24—O24	−179.65 (16)
N1—C2—C3—N4	57.68 (18)	C22—C23—C24—C25	1.0 (3)
C2—C3—N4—C21	171.43 (13)	C23—C24—C25—C26	−0.1 (3)
C2—C3—N4—C5	−57.26 (17)	O24—C24—C25—C26	−179.42 (16)
C21—N4—C5—C6	−171.06 (12)	C24—C25—C26—C21	−0.7 (3)
C3—N4—C5—C6	57.15 (17)	C22—C21—C26—C25	0.4 (2)
C2—N1—C6—C5	56.57 (17)	N4—C21—C26—C25	178.39 (14)
N4—C5—C6—N1	−57.02 (18)	C23—C24—O24—C27	−4.6 (3)
C3—N4—C21—C22	−3.3 (2)	C25—C24—O24—C27	174.67 (18)
C5—N4—C21—C22	−132.32 (16)	O31—C31—C32—C33	−173.24 (17)
C3—N4—C21—C26	178.91 (14)	O32—C31—C32—C33	6.0 (2)
C5—N4—C21—C26	49.85 (18)	C31—C32—C33—C34	−0.5 (3)
C26—C21—C22—C23	0.6 (2)	C32—C33—C34—O34	173.69 (16)
N4—C21—C22—C23	−177.33 (14)	C32—C33—C34—O33	−6.1 (2)
C21—C22—C23—C24	−1.3 (3)		

### 4-(4-Methoxyphenyl)piperazin-1-ium hydrogenmaleate (X) Hydrogen-bond geometry (Å, º)

**Table:**

*D*—H···*A*	*D*—H	H···*A*	*D*···*A*	*D*—H···*A*
O33—H33*A*···O32	1.167 (18)	1.247 (18)	2.4121 (16)	175 (2)
N1—H11···O31	0.915 (17)	2.126 (16)	2.9309 (19)	146.2 (15)
N1—H11···O32	0.915 (17)	2.296 (17)	3.0798 (18)	143.5 (14)
N1—H12···O34^i^	0.919 (18)	1.881 (18)	2.7563 (17)	158.5 (17)
C2—H2*A*···O34^ii^	0.97	2.56	3.363 (2)	140

Symmetry codes: (i) *x*−1, *y*, *z*; (ii) −*x*+2, −*y*+1, −*z*+1.

### 4-(4-Methoxyphenyl)piperazin-1-ium trichloroacetate (XI) Crystal data

**Table:**

C_11_H_17_N_2_O^+^·C_2_Cl_3_O_2_^−^	*D*_x_ = 1.477 Mg m^−^^3^
*M**_r_* = 355.64	Mo *K*α radiation, λ = 0.71073 Å
Orthorhombic, *P**c**a*2_1_	Cell parameters from 2428 reflections
*a* = 10.6117 (11) Å	θ = 3.0–27.7°
*b* = 13.808 (1) Å	µ = 0.58 mm^−^^1^
*c* = 10.9137 (8) Å	*T* = 296 K
*V* = 1599.1 (2) Å^3^	Block, colourless
*Z* = 4	0.48 × 0.48 × 0.20 mm
*F*(000) = 736	

### 4-(4-Methoxyphenyl)piperazin-1-ium trichloroacetate (XI) Data collection

**Table:**

Oxford Diffraction Xcalibur with Sapphire CCD diffractometer	2428 independent reflections
Radiation source: Enhance (Mo) X-ray Source	2278 reflections with *I* > 2σ(*I*)
Graphite monochromator	*R*_int_ = 0.027
ω scans	θ_max_ = 27.7°, θ_min_ = 3.0°
Absorption correction: multi-scan (CrysAlis RED; Oxford Diffraction, 2009)	*h* = −5→13
*T*_min_ = 0.476, *T*_max_ = 0.892	*k* = −16→17
6173 measured reflections	*l* = −14→5

### 4-(4-Methoxyphenyl)piperazin-1-ium trichloroacetate (XI) Refinement

**Table:**

Refinement on *F*^2^	H atoms treated by a mixture of independent and constrained refinement
Least-squares matrix: full	*w* = 1/[σ^2^(*F*_o_^2^) + (0.0532*P*)^2^ + 0.3843*P*] where *P* = (*F*_o_^2^ + 2*F*_c_^2^)/3
*R*[*F*^2^ > 2σ(*F*^2^)] = 0.032	(Δ/σ)_max_ < 0.001
*wR*(*F*^2^) = 0.086	Δρ_max_ = 0.25 e Å^−^^3^
*S* = 1.08	Δρ_min_ = −0.30 e Å^−^^3^
2428 reflections	Extinction correction: SHELXL, Fc^*^=kFc[1+0.001xFc^2^λ^3^/sin(2θ)]^-1/4^
198 parameters	Extinction coefficient: 0.023 (2)
1 restraint	Absolute structure: Classical Flack method preferred over Parsons because s.u. lower
Primary atom site location: difference Fourier map	Absolute structure parameter: 0.11 (7)
Hydrogen site location: mixed	

### 4-(4-Methoxyphenyl)piperazin-1-ium trichloroacetate (XI) Special details

**Table:**

Experimental. Compound (XI). IR (KBr , cm^-1^) 3073 (NH_2_), 2829 (OCH_3_), 1561 (COO). NMR (CDCl_3_) δ(^1^H) ) 3.07 (m, 4H, piperazine), 3.19 (m, 4H, piperazine), 3.77 (s, 3H, OCH_3_), 6.89 (m, 4H, methoxyphenyl).
Geometry. All esds (except the esd in the dihedral angle between two l.s. planes) are estimated using the full covariance matrix. The cell esds are taken into account individually in the estimation of esds in distances, angles and torsion angles; correlations between esds in cell parameters are only used when they are defined by crystal symmetry. An approximate (isotropic) treatment of cell esds is used for estimating esds involving l.s. planes.

### 4-(4-Methoxyphenyl)piperazin-1-ium trichloroacetate (XI) Fractional atomic coordinates and isotropic or equivalent isotropic displacement parameters (Å^2^)

**Table:**

	*x*	*y*	*z*	*U*_iso_*/*U*_eq_	
N1	0.1394 (2)	0.44188 (17)	0.2473 (3)	0.0301 (5)	
H11	0.168 (3)	0.495 (3)	0.204 (3)	0.036*	
H12	0.061 (3)	0.426 (3)	0.206 (4)	0.036*	
C2	0.1158 (3)	0.4667 (2)	0.3775 (3)	0.0343 (7)	
H2A	0.0542	0.5185	0.3822	0.041*	
H2B	0.1933	0.4895	0.4148	0.041*	
C3	0.0680 (3)	0.37961 (19)	0.4468 (3)	0.0320 (6)	
H3A	0.0546	0.3967	0.5320	0.038*	
H3B	−0.0121	0.3591	0.4126	0.038*	
N4	0.1586 (2)	0.30039 (15)	0.4389 (2)	0.0261 (5)	
C5	0.1815 (3)	0.2750 (2)	0.3106 (3)	0.0311 (6)	
H5A	0.1037	0.2523	0.2738	0.037*	
H5B	0.2424	0.2227	0.3067	0.037*	
C6	0.2304 (2)	0.3607 (2)	0.2392 (3)	0.0324 (6)	
H6A	0.3112	0.3808	0.2721	0.039*	
H6B	0.2423	0.3426	0.1541	0.039*	
C21	0.1350 (2)	0.22071 (19)	0.5194 (3)	0.0269 (6)	
C22	0.0345 (3)	0.2170 (2)	0.5993 (3)	0.0347 (6)	
H22	−0.0265	0.2653	0.5959	0.042*	
C23	0.0216 (3)	0.1433 (2)	0.6846 (3)	0.0396 (7)	
H23	−0.0468	0.1430	0.7379	0.048*	
C24	0.1103 (3)	0.0700 (2)	0.6906 (3)	0.0398 (7)	
C25	0.2088 (3)	0.0707 (2)	0.6074 (4)	0.0388 (7)	
H25	0.2671	0.0205	0.6083	0.047*	
C26	0.2220 (3)	0.14432 (19)	0.5235 (3)	0.0324 (6)	
H26	0.2892	0.1434	0.4689	0.039*	
O24	0.1108 (3)	−0.00368 (18)	0.7747 (3)	0.0587 (8)	
C27	0.0095 (6)	−0.0086 (4)	0.8579 (5)	0.0733 (13)	
H27A	0.0234	−0.0608	0.9145	0.110*	
H27B	−0.0675	−0.0197	0.8138	0.110*	
H27C	0.0035	0.0513	0.9022	0.110*	
C31	0.3163 (2)	0.61757 (18)	0.1132 (3)	0.0266 (5)	
O31	0.20314 (18)	0.60081 (16)	0.1045 (3)	0.0444 (6)	
O32	0.39463 (19)	0.57402 (18)	0.1749 (3)	0.0464 (6)	
C32	0.3653 (3)	0.7076 (2)	0.0383 (3)	0.0304 (6)	
Cl1	0.52532 (7)	0.69160 (6)	−0.00662 (9)	0.0426 (2)	
Cl2	0.35730 (10)	0.80958 (5)	0.13523 (10)	0.0505 (3)	
Cl3	0.27618 (8)	0.72790 (7)	−0.09480 (9)	0.0540 (3)	

### 4-(4-Methoxyphenyl)piperazin-1-ium trichloroacetate (XI) Atomic displacement parameters (Å^2^)

**Table:**

	*U*^11^	*U*^22^	*U*^33^	*U*^12^	*U*^13^	*U*^23^
N1	0.0286 (11)	0.0228 (11)	0.0389 (15)	−0.0020 (8)	−0.0003 (11)	0.0038 (11)
C2	0.0433 (14)	0.0199 (13)	0.0398 (18)	0.0022 (11)	−0.0004 (14)	−0.0032 (12)
C3	0.0354 (13)	0.0242 (13)	0.0364 (16)	0.0059 (11)	0.0060 (12)	−0.0015 (12)
N4	0.0304 (11)	0.0178 (10)	0.0300 (13)	0.0008 (8)	0.0049 (9)	−0.0026 (9)
C5	0.0370 (14)	0.0234 (13)	0.0331 (15)	0.0034 (11)	0.0027 (13)	−0.0025 (12)
C6	0.0308 (14)	0.0307 (14)	0.0356 (16)	0.0041 (10)	0.0064 (13)	0.0002 (13)
C21	0.0295 (11)	0.0196 (11)	0.0316 (16)	−0.0051 (9)	0.0001 (11)	−0.0022 (11)
C22	0.0335 (13)	0.0295 (13)	0.0411 (18)	−0.0016 (11)	0.0036 (13)	−0.0032 (13)
C23	0.0455 (15)	0.0342 (15)	0.0392 (19)	−0.0102 (13)	0.0117 (14)	−0.0011 (14)
C24	0.0628 (19)	0.0206 (12)	0.0361 (18)	−0.0128 (13)	−0.0005 (15)	−0.0005 (13)
C25	0.0487 (15)	0.0198 (12)	0.048 (2)	0.0005 (11)	−0.0002 (16)	0.0004 (13)
C26	0.0336 (13)	0.0229 (13)	0.0408 (18)	−0.0009 (10)	0.0034 (12)	−0.0015 (13)
O24	0.0943 (19)	0.0332 (12)	0.0485 (17)	−0.0085 (13)	0.0079 (15)	0.0129 (13)
C27	0.095 (3)	0.064 (3)	0.061 (3)	−0.028 (2)	0.008 (3)	0.023 (2)
C31	0.0303 (12)	0.0190 (11)	0.0305 (15)	−0.0017 (9)	0.0033 (12)	0.0005 (11)
O31	0.0301 (9)	0.0391 (11)	0.0640 (17)	−0.0070 (8)	−0.0039 (11)	0.0214 (12)
O32	0.0328 (11)	0.0421 (12)	0.0643 (18)	−0.0046 (9)	−0.0072 (11)	0.0244 (12)
C32	0.0320 (12)	0.0260 (13)	0.0332 (15)	−0.0049 (10)	−0.0009 (12)	0.0029 (12)
Cl1	0.0343 (3)	0.0457 (4)	0.0478 (5)	−0.0125 (3)	0.0085 (3)	−0.0007 (4)
Cl2	0.0714 (6)	0.0236 (3)	0.0565 (6)	−0.0017 (3)	0.0089 (5)	−0.0065 (4)
Cl3	0.0514 (5)	0.0652 (6)	0.0455 (5)	−0.0134 (4)	−0.0124 (4)	0.0233 (5)

### 4-(4-Methoxyphenyl)piperazin-1-ium trichloroacetate (XI) Geometric parameters (Å, º)

**Table:**

N1—C6	1.482 (3)	C22—C23	1.386 (5)
N1—C2	1.484 (4)	C22—H22	0.9300
N1—H11	0.92 (4)	C23—C24	1.383 (5)
N1—H12	0.97 (3)	C23—H23	0.9300
C2—C3	1.509 (4)	C24—O24	1.370 (4)
C2—H2A	0.9700	C24—C25	1.385 (5)
C2—H2B	0.9700	C25—C26	1.375 (4)
C3—N4	1.459 (3)	C25—H25	0.9300
C3—H3A	0.9700	C26—H26	0.9300
C3—H3B	0.9700	O24—C27	1.410 (6)
N4—C21	1.430 (4)	C27—H27A	0.9600
N4—C5	1.464 (4)	C27—H27B	0.9600
C5—C6	1.509 (4)	C27—H27C	0.9600
C5—H5A	0.9700	C31—O31	1.227 (3)
C5—H5B	0.9700	C31—O32	1.227 (4)
C6—H6A	0.9700	C31—C32	1.576 (4)
C6—H6B	0.9700	C32—Cl3	1.756 (3)
C21—C22	1.379 (4)	C32—Cl2	1.763 (3)
C21—C26	1.402 (4)	C32—Cl1	1.781 (3)
			
C6—N1—C2	110.0 (2)	C22—C21—N4	123.5 (3)
C6—N1—H11	111 (2)	C26—C21—N4	118.9 (2)
C2—N1—H11	111 (2)	C21—C22—C23	121.9 (3)
C6—N1—H12	111 (2)	C21—C22—H22	119.0
C2—N1—H12	111 (2)	C23—C22—H22	119.0
H11—N1—H12	103 (3)	C24—C23—C22	120.1 (3)
N1—C2—C3	110.6 (2)	C24—C23—H23	119.9
N1—C2—H2A	109.5	C22—C23—H23	119.9
C3—C2—H2A	109.5	O24—C24—C23	125.3 (3)
N1—C2—H2B	109.5	O24—C24—C25	116.2 (3)
C3—C2—H2B	109.5	C23—C24—C25	118.5 (3)
H2A—C2—H2B	108.1	C26—C25—C24	121.3 (3)
N4—C3—C2	110.3 (2)	C26—C25—H25	119.4
N4—C3—H3A	109.6	C24—C25—H25	119.4
C2—C3—H3A	109.6	C25—C26—C21	120.7 (3)
N4—C3—H3B	109.6	C25—C26—H26	119.7
C2—C3—H3B	109.6	C21—C26—H26	119.7
H3A—C3—H3B	108.1	C24—O24—C27	117.7 (3)
C21—N4—C3	115.2 (2)	O24—C27—H27A	109.5
C21—N4—C5	115.6 (2)	O24—C27—H27B	109.5
C3—N4—C5	110.2 (2)	H27A—C27—H27B	109.5
N4—C5—C6	111.3 (2)	O24—C27—H27C	109.5
N4—C5—H5A	109.4	H27A—C27—H27C	109.5
C6—C5—H5A	109.4	H27B—C27—H27C	109.5
N4—C5—H5B	109.4	O31—C31—O32	127.8 (3)
C6—C5—H5B	109.4	O31—C31—C32	115.6 (2)
H5A—C5—H5B	108.0	O32—C31—C32	116.6 (2)
N1—C6—C5	109.8 (2)	C31—C32—Cl3	112.15 (19)
N1—C6—H6A	109.7	C31—C32—Cl2	107.6 (2)
C5—C6—H6A	109.7	Cl3—C32—Cl2	110.05 (16)
N1—C6—H6B	109.7	C31—C32—Cl1	111.06 (19)
C5—C6—H6B	109.7	Cl3—C32—Cl1	107.82 (18)
H6A—C6—H6B	108.2	Cl2—C32—Cl1	108.08 (15)
C22—C21—C26	117.4 (3)		
			
C6—N1—C2—C3	−57.4 (3)	C22—C23—C24—O24	176.6 (3)
N1—C2—C3—N4	58.2 (3)	C22—C23—C24—C25	−2.1 (5)
C2—C3—N4—C21	168.8 (3)	O24—C24—C25—C26	−176.2 (3)
C2—C3—N4—C5	−58.2 (3)	C23—C24—C25—C26	2.6 (5)
C21—N4—C5—C6	−168.7 (2)	C24—C25—C26—C21	−0.4 (5)
C3—N4—C5—C6	58.5 (3)	C22—C21—C26—C25	−2.3 (4)
C2—N1—C6—C5	56.6 (3)	N4—C21—C26—C25	174.1 (3)
N4—C5—C6—N1	−57.6 (3)	C23—C24—O24—C27	3.5 (5)
C3—N4—C21—C22	0.2 (4)	C25—C24—O24—C27	−177.7 (4)
C5—N4—C21—C22	−130.3 (3)	O31—C31—C32—Cl3	−29.9 (3)
C3—N4—C21—C26	−175.9 (3)	O32—C31—C32—Cl3	151.5 (3)
C5—N4—C21—C26	53.6 (3)	O31—C31—C32—Cl2	91.3 (3)
C26—C21—C22—C23	2.8 (5)	O32—C31—C32—Cl2	−87.3 (3)
N4—C21—C22—C23	−173.3 (3)	O31—C31—C32—Cl1	−150.6 (2)
C21—C22—C23—C24	−0.7 (5)	O32—C31—C32—Cl1	30.8 (4)

### 4-(4-Methoxyphenyl)piperazin-1-ium trichloroacetate (XI) Hydrogen-bond geometry (Å, º)

**Table:**

*D*—H···*A*	*D*—H	H···*A*	*D*···*A*	*D*—H···*A*
N1—H11···O31	0.92 (4)	1.86 (4)	2.775 (4)	172 (3)
N1—H12···O32^i^	0.97 (3)	1.80 (3)	2.724 (3)	158 (3)

Symmetry code: (i) *x*−1/2, −*y*+1, *z*.

### Bis(4-(4-methoxyphenyl)piperazin-1-ium) chloranilate(2-) dihydrate (XII) Crystal data

**Table:**

C_11_H_17_N_2_O^+^·0.5C_6_Cl_2_O_4_^2^^−^·H_2_O	*F*(000) = 664
*M**_r_* = 314.76	*D*_x_ = 1.417 Mg m^−^^3^
Monoclinic, *P*2_1_/*n*	Mo *K*α radiation, λ = 0.71073 Å
*a* = 9.1597 (5) Å	Cell parameters from 3253 reflections
*b* = 15.1434 (8) Å	θ = 2.6–28.0°
*c* = 10.8742 (6) Å	µ = 0.28 mm^−^^1^
β = 102.067 (5)°	*T* = 296 K
*V* = 1475.02 (14) Å^3^	Block, colourless
*Z* = 4	0.44 × 0.24 × 0.20 mm

### Bis(4-(4-methoxyphenyl)piperazin-1-ium) chloranilate(2-) dihydrate (XII) Data collection

**Table:**

Oxford Diffraction Xcalibur with Sapphire CCD diffractometer	9650 independent reflections
Radiation source: Enhance (Mo) X-ray Source	7444 reflections with *I* > 2σ(*I*)
Graphite monochromator	θ_max_ = 27.6°, θ_min_ = 2.6°
ω scans	*h* = −11→11
Absorption correction: multi-scan (CrysAlis RED; Oxford Diffraction, 2009)	*k* = −19→19
*T*_min_ = 0.892, *T*_max_ = 0.947	*l* = −13→14
9650 measured reflections	

### Bis(4-(4-methoxyphenyl)piperazin-1-ium) chloranilate(2-) dihydrate (XII) Refinement

**Table:**

Refinement on *F*^2^	0 restraints
Least-squares matrix: full	Hydrogen site location: mixed
*R*[*F*^2^ > 2σ(*F*^2^)] = 0.039	H atoms treated by a mixture of independent and constrained refinement
*wR*(*F*^2^) = 0.105	*w* = 1/[σ^2^(*F*_o_^2^) + (0.0573*P*)^2^ + 0.263*P*] where *P* = (*F*_o_^2^ + 2*F*_c_^2^)/3
*S* = 1.02	(Δ/σ)_max_ < 0.001
9650 reflections	Δρ_max_ = 0.23 e Å^−^^3^
204 parameters	Δρ_min_ = −0.32 e Å^−^^3^

### Bis(4-(4-methoxyphenyl)piperazin-1-ium) chloranilate(2-) dihydrate (XII) Special details

**Table:**

Experimental. Compound (XII). IR (KBr , cm^-1^) 3311 (OH), 3073 (NH_2_), 2825 (OCH_3_), 1561 (COO), 793 and 741 (CCl) . NMR (CDCl_3_) δ(^1^H) ) 3.11 (m, 4H, piperazine), 3.40 (m, 4H, piperazine), 3.77 (s, 3H, OCH_3_), 6.88 (m, 4H, methoxyphenyl).
Geometry. All esds (except the esd in the dihedral angle between two l.s. planes) are estimated using the full covariance matrix. The cell esds are taken into account individually in the estimation of esds in distances, angles and torsion angles; correlations between esds in cell parameters are only used when they are defined by crystal symmetry. An approximate (isotropic) treatment of cell esds is used for estimating esds involving l.s. planes.
Refinement. Refined as a 2-component twin.

### Bis(4-(4-methoxyphenyl)piperazin-1-ium) chloranilate(2-) dihydrate (XII) Fractional atomic coordinates and isotropic or equivalent isotropic displacement parameters (Å^2^)

**Table:**

	*x*	*y*	*z*	*U*_iso_*/*U*_eq_	
N1	0.4381 (2)	0.32824 (14)	0.35576 (19)	0.0420 (5)	
H11	0.437 (2)	0.3477 (15)	0.278 (3)	0.050*	
H12	0.420 (3)	0.3725 (16)	0.405 (2)	0.050*	
C2	0.5890 (2)	0.29024 (17)	0.4015 (2)	0.0472 (6)	
H2A	0.6629	0.3370	0.4125	0.057*	
H2B	0.6115	0.2490	0.3397	0.057*	
C3	0.5965 (2)	0.24346 (16)	0.5241 (2)	0.0427 (6)	
H3A	0.6939	0.2164	0.5509	0.051*	
H3B	0.5827	0.2856	0.5878	0.051*	
N4	0.48097 (19)	0.17569 (13)	0.51047 (18)	0.0398 (5)	
C5	0.3334 (2)	0.21513 (17)	0.4745 (2)	0.0455 (6)	
H5A	0.3186	0.2572	0.5381	0.055*	
H5B	0.2576	0.1696	0.4681	0.055*	
C6	0.3188 (2)	0.26139 (16)	0.3494 (2)	0.0454 (6)	
H6A	0.3256	0.2184	0.2847	0.054*	
H6B	0.2221	0.2899	0.3271	0.054*	
C21	0.5025 (2)	0.10929 (15)	0.6041 (2)	0.0357 (5)	
C22	0.6331 (2)	0.05976 (16)	0.6228 (2)	0.0432 (6)	
H22	0.7033	0.0719	0.5744	0.052*	
C23	0.6610 (2)	−0.00626 (16)	0.7103 (2)	0.0457 (6)	
H23	0.7503	−0.0375	0.7222	0.055*	
C24	0.5565 (2)	−0.02672 (15)	0.7812 (2)	0.0418 (6)	
C25	0.4243 (2)	0.01896 (16)	0.7614 (2)	0.0452 (6)	
H25	0.3516	0.0040	0.8059	0.054*	
C26	0.3991 (2)	0.08760 (16)	0.6747 (2)	0.0419 (6)	
H26	0.3106	0.1195	0.6641	0.050*	
O24	0.59700 (19)	−0.09318 (12)	0.86827 (19)	0.0615 (5)	
C27	0.4842 (3)	−0.12833 (17)	0.9257 (3)	0.0577 (7)	
H27A	0.5208	−0.1806	0.9720	0.086*	
H27B	0.3980	−0.1429	0.8621	0.086*	
H27C	0.4573	−0.0853	0.9820	0.086*	
C31	0.4846 (2)	0.41334 (14)	0.0515 (2)	0.0301 (5)	
O31	0.46952 (17)	0.34242 (10)	0.10569 (14)	0.0429 (4)	
C32	0.4674 (2)	0.42353 (14)	−0.0785 (2)	0.0313 (5)	
Cl32	0.42518 (7)	0.33070 (4)	−0.17371 (6)	0.04725 (19)	
O33	0.54043 (16)	0.48217 (10)	0.25106 (14)	0.0419 (4)	
C33	0.5224 (2)	0.49523 (14)	0.13592 (19)	0.0297 (5)	
O41	0.3458 (2)	0.45297 (16)	0.5134 (2)	0.0698 (6)	
H41	0.383 (4)	0.464 (2)	0.589 (4)	0.105*	
H42	0.295 (4)	0.497 (2)	0.492 (3)	0.105*	

### Bis(4-(4-methoxyphenyl)piperazin-1-ium) chloranilate(2-) dihydrate (XII) Atomic displacement parameters (Å^2^)

**Table:**

	*U*^11^	*U*^22^	*U*^33^	*U*^12^	*U*^13^	*U*^23^
N1	0.0531 (12)	0.0465 (13)	0.0271 (11)	0.0008 (11)	0.0101 (9)	0.0103 (10)
C2	0.0458 (13)	0.0526 (15)	0.0447 (15)	−0.0023 (12)	0.0132 (11)	0.0145 (13)
C3	0.0397 (12)	0.0497 (14)	0.0362 (14)	−0.0084 (12)	0.0026 (10)	0.0099 (12)
N4	0.0358 (10)	0.0461 (11)	0.0344 (11)	−0.0073 (9)	0.0003 (8)	0.0123 (9)
C5	0.0372 (12)	0.0559 (16)	0.0411 (14)	−0.0077 (12)	0.0028 (10)	0.0152 (13)
C6	0.0462 (13)	0.0509 (15)	0.0343 (14)	−0.0037 (12)	−0.0026 (10)	0.0082 (12)
C21	0.0368 (12)	0.0404 (13)	0.0280 (12)	−0.0096 (11)	0.0027 (9)	0.0032 (10)
C22	0.0374 (13)	0.0471 (15)	0.0472 (15)	−0.0058 (11)	0.0139 (11)	0.0057 (12)
C23	0.0358 (12)	0.0451 (14)	0.0566 (17)	0.0018 (11)	0.0103 (11)	0.0088 (13)
C24	0.0462 (13)	0.0399 (14)	0.0375 (14)	−0.0005 (12)	0.0043 (10)	0.0093 (11)
C25	0.0429 (13)	0.0540 (16)	0.0413 (15)	−0.0008 (12)	0.0146 (10)	0.0123 (12)
C26	0.0358 (12)	0.0498 (15)	0.0398 (14)	0.0045 (11)	0.0070 (10)	0.0125 (12)
O24	0.0567 (11)	0.0620 (12)	0.0673 (14)	0.0079 (9)	0.0164 (9)	0.0339 (11)
C27	0.0752 (18)	0.0480 (16)	0.0522 (18)	−0.0012 (15)	0.0190 (14)	0.0151 (14)
C31	0.0292 (10)	0.0331 (12)	0.0290 (12)	0.0005 (9)	0.0083 (8)	0.0032 (10)
O31	0.0612 (10)	0.0355 (9)	0.0327 (9)	−0.0024 (8)	0.0115 (7)	0.0066 (7)
C32	0.0368 (11)	0.0315 (11)	0.0256 (11)	−0.0004 (10)	0.0060 (8)	−0.0010 (9)
Cl32	0.0648 (4)	0.0407 (3)	0.0347 (3)	−0.0058 (3)	0.0068 (3)	−0.0065 (3)
O33	0.0582 (9)	0.0456 (10)	0.0215 (8)	−0.0073 (8)	0.0073 (7)	0.0036 (7)
C33	0.0282 (10)	0.0381 (12)	0.0232 (11)	0.0019 (9)	0.0065 (8)	0.0014 (9)
O41	0.0876 (16)	0.0781 (15)	0.0408 (12)	0.0001 (12)	0.0066 (10)	−0.0146 (12)

### Bis(4-(4-methoxyphenyl)piperazin-1-ium) chloranilate(2-) dihydrate (XII) Geometric parameters (Å, º)

**Table:**

N1—C6	1.481 (3)	C23—C24	1.384 (3)
N1—C2	1.484 (3)	C23—H23	0.9300
N1—H11	0.89 (3)	C24—C25	1.372 (3)
N1—H12	0.89 (2)	C24—O24	1.379 (3)
C2—C3	1.498 (3)	C25—C26	1.390 (3)
C2—H2A	0.9700	C25—H25	0.9300
C2—H2B	0.9700	C26—H26	0.9300
C3—N4	1.459 (3)	O24—C27	1.419 (3)
C3—H3A	0.9700	C27—H27A	0.9600
C3—H3B	0.9700	C27—H27B	0.9600
N4—C21	1.415 (3)	C27—H27C	0.9600
N4—C5	1.455 (3)	C31—O31	1.246 (2)
C5—C6	1.511 (3)	C31—C32	1.398 (3)
C5—H5A	0.9700	C31—C33	1.539 (3)
C5—H5B	0.9700	C32—C33^i^	1.392 (3)
C6—H6A	0.9700	C32—Cl32	1.741 (2)
C6—H6B	0.9700	O33—C33	1.244 (2)
C21—C26	1.378 (3)	C33—C32^i^	1.392 (3)
C21—C22	1.390 (3)	O41—H41	0.83 (4)
C22—C23	1.367 (3)	O41—H42	0.82 (3)
C22—H22	0.9300		
			
C6—N1—C2	112.16 (18)	C26—C21—N4	124.1 (2)
C6—N1—H11	108.7 (15)	C22—C21—N4	118.3 (2)
C2—N1—H11	105.7 (14)	C23—C22—C21	121.7 (2)
C6—N1—H12	108.2 (15)	C23—C22—H22	119.1
C2—N1—H12	111.7 (16)	C21—C22—H22	119.1
H11—N1—H12	110 (2)	C22—C23—C24	120.1 (2)
N1—C2—C3	110.34 (18)	C22—C23—H23	120.0
N1—C2—H2A	109.6	C24—C23—H23	120.0
C3—C2—H2A	109.6	C25—C24—O24	125.3 (2)
N1—C2—H2B	109.6	C25—C24—C23	119.4 (2)
C3—C2—H2B	109.6	O24—C24—C23	115.3 (2)
H2A—C2—H2B	108.1	C24—C25—C26	120.0 (2)
N4—C3—C2	110.20 (19)	C24—C25—H25	120.0
N4—C3—H3A	109.6	C26—C25—H25	120.0
C2—C3—H3A	109.6	C21—C26—C25	121.3 (2)
N4—C3—H3B	109.6	C21—C26—H26	119.4
C2—C3—H3B	109.6	C25—C26—H26	119.4
H3A—C3—H3B	108.1	C24—O24—C27	117.40 (19)
C21—N4—C5	117.85 (18)	O24—C27—H27A	109.5
C21—N4—C3	115.96 (17)	O24—C27—H27B	109.5
C5—N4—C3	110.60 (18)	H27A—C27—H27B	109.5
N4—C5—C6	109.54 (18)	O24—C27—H27C	109.5
N4—C5—H5A	109.8	H27A—C27—H27C	109.5
C6—C5—H5A	109.8	H27B—C27—H27C	109.5
N4—C5—H5B	109.8	O31—C31—C32	125.0 (2)
C6—C5—H5B	109.8	O31—C31—C33	116.47 (18)
H5A—C5—H5B	108.2	C32—C31—C33	118.55 (18)
N1—C6—C5	110.47 (18)	C31—C32—C33^i^	123.2 (2)
N1—C6—H6A	109.6	C31—C32—Cl32	118.45 (17)
C5—C6—H6A	109.6	C33^i^—C32—Cl32	118.31 (16)
N1—C6—H6B	109.6	O33—C33—C32^i^	125.8 (2)
C5—C6—H6B	109.6	O33—C33—C31	115.99 (19)
H6A—C6—H6B	108.1	C32^i^—C33—C31	118.20 (17)
C26—C21—C22	117.5 (2)	H41—O41—H42	102 (3)
			
C6—N1—C2—C3	−53.5 (3)	C22—C23—C24—O24	−178.8 (2)
N1—C2—C3—N4	56.5 (3)	O24—C24—C25—C26	176.9 (2)
C2—C3—N4—C21	161.1 (2)	C23—C24—C25—C26	−2.8 (4)
C2—C3—N4—C5	−61.3 (3)	C22—C21—C26—C25	0.1 (3)
C21—N4—C5—C6	−162.3 (2)	N4—C21—C26—C25	177.1 (2)
C3—N4—C5—C6	61.0 (3)	C24—C25—C26—C21	2.3 (4)
C2—N1—C6—C5	53.7 (3)	C25—C24—O24—C27	12.2 (4)
N4—C5—C6—N1	−56.7 (3)	C23—C24—O24—C27	−168.1 (2)
C5—N4—C21—C26	−8.7 (3)	O31—C31—C32—C33^i^	176.5 (2)
C3—N4—C21—C26	125.7 (2)	C33—C31—C32—C33^i^	−2.4 (3)
C5—N4—C21—C22	168.2 (2)	O31—C31—C32—Cl32	−1.3 (3)
C3—N4—C21—C22	−57.4 (3)	C33—C31—C32—Cl32	179.81 (14)
C26—C21—C22—C23	−2.0 (3)	O31—C31—C33—O33	2.8 (3)
N4—C21—C22—C23	−179.1 (2)	C32—C31—C33—O33	−178.16 (18)
C21—C22—C23—C24	1.5 (4)	O31—C31—C33—C32^i^	−176.76 (18)
C22—C23—C24—C25	0.9 (4)	C32—C31—C33—C32^i^	2.2 (3)

Symmetry code: (i) −*x*+1, −*y*+1, −*z*.

### Bis(4-(4-methoxyphenyl)piperazin-1-ium) chloranilate(2-) dihydrate (XII) Hydrogen-bond geometry (Å, º)

**Table:**

*D*—H···*A*	*D*—H	H···*A*	*D*···*A*	*D*—H···*A*
N1—H11···O31	0.89 (3)	1.96 (3)	2.802 (3)	157 (2)
N1—H11···O33	0.89 (3)	2.29 (2)	2.838 (3)	119 (2)
N1—H12···O41	0.90 (2)	1.92 (2)	2.798 (3)	168 (3)
O41—H41···O33^ii^	0.84 (4)	1.92 (4)	2.738 (3)	166 (3)
O41—H42···O24^iii^	0.82 (3)	2.49 (3)	3.269 (3)	160 (3)

Symmetry codes: (ii) −*x*+1, −*y*+1, −*z*+1; (iii) *x*−1/2, −*y*+1/2, *z*−1/2.

## References

[bb1] Acosta, L. M., Bahsas, A., Palma, A., Cobo, J., Hursthouse, M. B. & Glidewell, C. (2009). *Acta Cryst.* C**65**, o92–o96. 10.1107/S0108270109003370 19265230

[bb2] Allen, F. H., Kennard, O., Watson, D. G., Brammer, L., Orpen, A. G. & Taylor, R. (1987). *J. Chem. Soc. Perkin Trans. 2*, pp. S1–S19.

[bb3] Al-Omary, F. A. M., Ghabbour, H. A., El-Emam, A. A., Chidan Kumar, C. S. & Fun, H.-K. (2014). *Acta Cryst.* E**70**, o245–o246. 10.1107/S1600536814002256 PMC3998442 24764966

[bb4] Arbo, M. D., Bastos, M. L. & Carmo, H. F. (2012). *Drug Alcohol Depend.* **122**, 174–185. 10.1016/j.drugalcdep.2011.10.007 22071119

[bb5] Asif, M. (2015). *Int. J. Adv. Sci. Res.* **1**, 5–11.

[bb6] Bernstein, J., Davis, R. E., Shimoni, L. & Chang, N.-L. (1995). *Angew. Chem. Int. Ed. Engl.* **34**, 1555–1573.

[bb7] Brito, A., Moreira, L. K. S., Menegatti, R. & Costa, E. A. (2019). *Fundam. Clin. Pharmacol.* **33**, 13–24. 10.1111/fcp.12408 30151922

[bb8] Emsley, J. (1980). *Chem. Soc. Rev.* **9**, 91–124.

[bb9] Etter, M. C. (1990). *Acc. Chem. Res.* **23**, 120–126.

[bb10] Etter, M. C., MacDonald, J. C. & Bernstein, J. (1990). *Acta Cryst.* B**46**, 256–262. 10.1107/s0108768189012929 2344397

[bb11] Ferguson, G., Glidewell, C., Gregson, R. M. & Meehan, P. R. (1998*a*). *Acta Cryst.* B**54**, 129–138.

[bb12] Ferguson, G., Glidewell, C., Gregson, R. M. & Meehan, P. R. (1998*b*). *Acta Cryst.* B**54**, 139–150.

[bb13] Flack, H. D. (1983). *Acta Cryst.* A**39**, 876–881.

[bb14] Flack, H. D. & Bernardinelli, G. (2000). *J. Appl. Cryst.* **33**, 1143–1148.

[bb15] Glidewell, C., Low, J. N., Skakle, J. M. S. & Wardell, J. L. (2005). *Acta Cryst.* C**61**, o276–o280. 10.1107/S0108270105007286 15876714

[bb16] Gregson, R. M., Glidewell, C., Ferguson, G. & Lough, A. J. (2000). *Acta Cryst.* B**56**, 39–57. 10.1107/s0108768199006072 10735443

[bb17] Herschlag, D. & Pinney, M. M. (2018). *Biochemistry*, **57**, 3338–3352. 10.1021/acs.biochem.8b00217 29678112

[bb18] Ishida, H. (2004*a*). *Acta Cryst.* E**60**, o974–o976.

[bb19] Ishida, H. (2004*b*). *Acta Cryst.* E**60**, o1900–o1901.

[bb20] Ishida, H. (2004*c*). *Acta Cryst.* E**60**, o2005–o2006.

[bb21] Ishida, H. (2004*d*). *Acta Cryst.* E**60**, o2506–o2508.

[bb22] Kaur, M., Jasinski, J. P., Yathirajan, H. S., Kavitha, C. N. & Glidewell, C. (2015). *Acta Cryst.* E**71**, 406–413. 10.1107/S205698901500554X PMC4438793 26029402

[bb23] Kavitha, C. N., Jasinski, J. P., Anderson, B. J., Yathirajan, H. S. & Kaur, M. (2013). *Acta Cryst.* E**69**, o1671. 10.1107/S1600536813028031 PMC3884329 24454105

[bb24] Kavitha, C. N., Yathirajan, H. S., Kaur, M., Hosten, E. C., Betz, R. & Glidewell, C. (2014). *Acta Cryst.* C**70**, 805–811. 10.1107/S2053229614016532 25093364

[bb25] Kavitha, S. J., Panchanatheswaran, K., Low, J. N., Ferguson, G. & Glidewell, C. (2006). *Acta Cryst.* C**62**, o165–o169. 10.1107/S0108270106005002 16598134

[bb26] Kiran Kumar, H., Yathirajan, H. S., Sagar, B. K., Foro, S. & Glidewell, C. (2019). *Acta Cryst.* E**75**, 1253–1260. 10.1107/S2056989019010491 PMC6690458 31417802

[bb27] Lu, Y.-X. (2007). *Acta Cryst.* E**63**, o3611.

[bb28] Nagai, F., Nonaka, R. & Kamimura, K. S. H. (2007). *Eur. J. Pharmacol.* **559**, 132–137. 10.1016/j.ejphar.2006.11.075 17223101

[bb29] Oxford Diffraction (2009). *CrysAlis CCD* and *CrysAlis RED*. Oxford Diffraction Ltd, Abingdon, England.

[bb30] Parsons, S., Flack, H. D. & Wagner, T. (2013). *Acta Cryst.* B**69**, 249–259. 10.1107/S2052519213010014 PMC3661305 23719469

[bb31] Sagar, B. K., Girisha, M., Yathirajan, H. S., Rathore, R. S. & Glidewell, C. (2017). *Acta Cryst.* E**73**, 1320–1325. 10.1107/S2056989017011446 PMC5588571 28932465

[bb32] Shaibah, M. A. E., Sagar, B. K., Yathirajan, H. S., Kumar, S. M. & Glidewell, C. (2017*a*). *Acta Cryst.* E**73**, 1513–1516. 10.1107/S205698901701324X PMC5730306 29250369

[bb33] Shaibah, M. A. E., Yathirajan, H. S., Kumar, S. M., Byrappa, K. & Glidewell, C. (2017*b*). *Acta Cryst.* E**73**, 1488–1492. 10.1107/S2056989017012968 PMC5730301 29250364

[bb34] Shaibah, M. A. E., Yathirajan, H. S., Rathore, R. S., Furuya, T., Haraguchi, T., Akitsu, T. & Glidewell, C. (2019). *Acta Cryst.* E**75**, 292–298. 10.1107/S2056989019001385 PMC6362656 30800470

[bb35] Sheldrick, G. M. (2015*a*). *Acta Cryst.* A**71**, 3–8.

[bb36] Sheldrick, G. M. (2015*b*). *Acta Cryst.* C**71**, 3–8.

[bb37] Sovago, I., Thomas, L. H., Adam, M. S., Capelli, S. C., Wilson, C. C. & Farrugia, L. J. (2016). *CrystEngComm*, **18**, 5697–5709.

[bb38] Spek, A. L. (2009). *Acta Cryst.* D**65**, 148–155. 10.1107/S090744490804362X PMC2631630 19171970

[bb39] Staack, R. F. & Maurer, H. H. (2003). *J. Chromatogr. B*, **798**, 333–342. 10.1016/j.jchromb.2003.10.004 14643514

[bb40] Staack, R. F. & Maurer, H. H. (2005). *Curr. Drug Metab.* **6**, 259–274. 10.2174/1389200054021825 15975043

[bb41] Staack, R. F., Theobald, D. S., Paul, D., Springer, D., Kraemer, T. & Maurer, H. H. (2004). *Xenobiotica*, **34**, 179–192. 10.1080/00498250310001644544 14985146

[bb42] Verdonk, M. L., Voogd, J. W., Kanters, J. A., Kroon, J., den Besten, R., Brandsma, L., Leysen, D. & Kelder, J. (1997). *Acta Cryst.* B**53**, 976–983. 10.1107/s0108768197009142 9436302

[bb43] Wohlfarth, A., Weinmann, W. & Dresen, S. (2010). *Anal. Bioanal. Chem.* **396**, 2403–2414. 10.1007/s00216-009-3394-4 20069283

[bb44] Wood, P. A., Allen, F. H. & Pidcock, E. (2009). *CrystEngComm*, **11**, 1563–1571.

[bb45] Zia-ur-Rehman, Tahir, M. N., Danish, M., Muhammad, N. & Ali, S. (2009). *Acta Cryst.* E**65**, o503. 10.1107/S1600536809004280 PMC2968517 21582167

